# Inference of dynamic interaction networks: A comparison between Lotka-Volterra and multivariate autoregressive models

**DOI:** 10.3389/fbinf.2022.1021838

**Published:** 2022-12-22

**Authors:** Daniel V. Olivença, Jacob D. Davis, Eberhard O. Voit

**Affiliations:** The Wallace H. Coulter Department of Biomedical Engineering, Georgia Institute of Technology and Emory University, Atlanta, GA, United States

**Keywords:** community dynamics, Lotka-Volterra model, multivariate autoregressive (MAR) model, network structure inference, parameter estimation, population dynamics, systems biology

## Abstract

Networks are ubiquitous throughout biology, spanning the entire range from molecules to food webs and global environmental systems. Yet, despite substantial efforts by the scientific community, the inference of these networks from data still presents a problem that is unsolved in general. One frequent strategy of addressing the structure of networks is the assumption that the interactions among molecular or organismal populations are static and correlative. While often successful, these static methods are no panacea. They usually ignore the asymmetry of relationships between two species and inferences become more challenging if the network nodes represent dynamically changing quantities. Overcoming these challenges, two very different network inference approaches have been proposed in the literature: Lotka-Volterra (LV) models and Multivariate Autoregressive (MAR) models. These models are computational frameworks with different mathematical structures which, nevertheless, have both been proposed for the same purpose of inferring the interactions within coexisting population networks from observed time-series data. Here, we assess these dynamic network inference methods for the first time in a side-by-side comparison, using both synthetically generated and ecological datasets. Multivariate Autoregressive and Lotka-Volterra models are mathematically equivalent at the steady state, but the results of our comparison suggest that Lotka-Volterra models are generally superior in capturing the dynamics of networks with non-linear dynamics, whereas Multivariate Autoregressive models are better suited for analyses of networks of populations with process noise and close-to linear behavior. To the best of our knowledge, this is the first study comparing LV and MAR approaches. Both frameworks are valuable tools that address slightly different aspects of dynamic networks.

## 1 Introduction

Living systems are notoriously complex due to the large number and variety of their components and the dynamic interactions among them. The overriding task of biology is to decipher how these components and interactions lead to functioning organisms and communities. Network science has proven instrumental for this task by developing methods for the extraction of information characterizing the structure and dynamics of these systems, not only in biology (Matchado et al., 2021) but in all fields of science that deal with complexity. As (Barabási, 2015) stated, “The exploding interest in network science during the first decade of the 21st century is rooted in the discovery that despite the obvious diversity of complex systems, the structure and the evolution of the networks behind each system is driven by a common set of fundamental laws and principles. Therefore, notwithstanding the amazing differences in form, size, nature, age, and scope of real networks, most networks are driven by common organizing principles. Once we disregard the nature of the components and the precise nature of the interactions between them, the obtained networks are more similar than different from each other.”

In biology, network inference has been an important endeavor in numerous diverse applications. In molecular biology, the characterization of gene regulatory and protein interaction networks has been a hallmark of progress over the past decades [e.g., (Bansal et al., 2007; Penfold and Wild, 2011)]. This characterization is often achieved with Bayesian methods (Kim, 2003; Friedman and Alm, 2012) or by computing mutual information between network components (Kim, 2003; Olsen et al., 2009; Friedman and Alm, 2012; Villaverde et al., 2014), but many other methods have been proposed [e.g., (Saint-Antoine and Singh, 2020)]. In ecology, and more recently in microbiome investigations, networks are at the core of assessing coexisting populations, and a variety of methodologies of analysis exists. Nonetheless, the inference of network structure from data is still an open problem (Matchado et al., 2021). In particular, no clear guidelines or gold standards exist, and none of the existing tools successfully addresses all issues of network inference. For instance, many methods have problems with identifying spurious relations within microbial communities. Consequently, the selection of the most appropriate technique is often made in an *ad hoc* manner, based on the characteristics of the available data and features like computational scalability.

The relationships among the species of microbial communities are traditionally assessed with network analyses of graph theory. The vertices in these networks represent the different species or operational taxonomic units (OTUs), while edges represent pairwise or complex relationships. The typical method of analysis of these types of data is the establishment of correlation networks based on the presence, absence, or abundance of the species across multiple locations or time points. Specifically, pairwise interactions are characterized with a similarity index or a modified Pearson Correlation Coefficient (Ruan et al., 2006; Barberán et al., 2012; Faust et al., 2012; Friedman and Alm, 2012; Gilbert et al., 2012), while more complex relationships are derived from regression or rule-based networks (Chaffron et al., 2010; Faust and Raes, 2012). Other methods include local similarity analysis, probabilistic graphical models, and matrix factorization techniques (Matchado et al., 2021).

While static correlation networks can address complex communities of thousands of species across multiple environments (Chaffron et al., 2010; Barberán et al., 2012), they do not capture potentially important dynamic trends and often ignore the asymmetry of relationships between species. Namely, the interactions in ecological networks are usually represented as undirected edges, although the effect of A on B is often qualitatively different from the effect of B on A. This issue can be remedied to some degree by the use of directed graphs (Matchado et al., 2021).

Network analysis faces three types of biases: compositionality, sparsity, and spurious associations. Data may be compositional if they only offer information about the relative abundance of populations. In sparse data, a zero may indicate either the absence of a population or insufficient reading depth, and these two explanations are indistinguishable. Finally, the association of two observed populations to an unobserved third can be wrongfully interpreted as a spurious association between the two observed populations. For more details regarding the inference of networks from data, see the review (Matchado et al., 2021).

The inference becomes even more challenging if the network nodes represent dynamically changing quantities, such as protein abundances during an immune response or different populations in a mixed community (Oates and Mukherjee, 2012). Many methods exist to study these dynamic changes, including local similarity analysis (Ruan et al., 2006) and dynamic Bayesian network analysis (De Smet and Marchal, 2010), but most of the existing microbial network tools emphasize nodes while giving interactions lower priority (Matchado et al., 2021).

A generic alternative to these approaches is a system of differential equations (Kirschner and Blaser, 1995; Liu et al., 2006; Balagaddé et al., 2008; Mounier et al., 2008; Faith et al., 2011; Hanly et al., 2012; Stein et al., 2013; Berry and Widder, 2014; Fisher and Mehta, 2014; Fujikawa and Sakha, 2014; Marino et al., 2014). These equations are naturally dynamic and typically include terms that describe growth and decay, pairwise interactions between species, and the effects of nutrients or other environmental factors (Stein et al., 2013). Among these approaches, Lotka-Volterra (LV) models have been used extensively since the mid-1920s to assess different types of interactions in dynamically changing populations; a small sample is (Liu et al., 2006; Balagaddé et al., 2008; Mounier et al., 2008; Stein et al., 2013; Berry and Widder, 2014; Fisher and Mehta, 2014; Fujikawa and Sakha, 2014; Marino et al., 2014). LV models were independently proposed by (Lotka, 1925), who studied periodic increases and decreases in the populations of lynx and hare in Canada, and (Volterra, 1926), who analyzed fish catches and the competition among marine populations in the Adriatic Sea. Since these early days, LV models have become a mainstay—and typical default—in theoretical ecology [e.g., (May, 2001)].

With the discovery of complex microbiomes and their surprisingly strong effects on human health and the environment, quantitative assessments of interactions among different species have received renewed attention (Gavin et al., 2006; Stein et al., 2013; Shenhav et al., 2019). As an example, we recently inferred the temporally changing interactions among bacterial communities in different lake environments with over 12,000 operational taxonomic units (OTUs) (Dam et al., 2020; Dam et al., 2016). We chose as our computational framework an LV model, which we augmented with LV equations for environmental variables that affect the OTUs. Our rationale for this choice was a combination of 1) the track record of successful applications of LV models, 2) their mathematical simplicity and tractability, 3) the straightforward option of incorporating time-dependent external perturbations (Stein et al., 2013), and 4) the important fact that parameter values—and thus signs and strengths of interactions—can be obtained from time series data of OTU abundances with methods of linear regression (Voit and Chou, 2010).

Multivariate Autoregressive (MAR) models were first proposed a few decades ago as a viable alternative to LV models. Originally proposed for problems in economics (Sims, 1980), Ives suggested their use for predicting responses of populations to environmental changes (Ives, 1995). His specific motivation was to establish techniques for studying how population abundances change in response to long-term environmental trends and for partitioning different factors that drive key changes in population densities in response to these trends. Since this early work, MAR models have been chosen to represent the interaction dynamics between biotic and abiotic drivers, infer the intra- and interspecific effects of species abundances on population growth rates, identify environmental drivers of community dynamics, predict the fate of communities exposed to environmental changes and extract measures of community stability and resilience; the latter was initially applied to lake and marine systems and later in terrestrial ecology (Certain et al., 2018).

LV systems are ODE models, whereas MAR systems are statistical models. The former were designed to elucidate the long-term dynamics of interacting populations, whereas the latter were conceived not only to study interacting population but also the stochastic structure of the supporting data. Thus, two modeling frameworks with different mathematical structures have been proposed for essentially the same purpose of extracting key features of dynamic interactions among coexisting species from observed time series data. Both methods have had successes, but a direct comparison of the two approaches has never been reported. Such a comparison is the subject of this article. Our focus for their comparison is the ability of each model framework to produce an acceptable fit to observation data, capture the process dynamics underlying the observed trends in population abundances and infer correct parameter sets as well as possible.

We use four versions of MAR: MAR without data transformation; MAR with log transformation, as often proposed by MAR users (Dennis and Taper, 1994; Ives, 1995; Certain et al., 2018); MAR upon data smoothing; and MAR with log transformation upon data smoothing. A log transformation is necessary for comparing the general mathematical interpretation of a MAR model with a widely used ecological interpretation, namely, as a multispecies competition model with Gompertz density dependence (Ives, 1995; Certain et al., 2018) (see also Supplementary Section S1.3).

Because noise is ubiquitous in real-world data, we explore for both frameworks the effects of data smoothing on the parameter inference results. We recognize that this smoothing step impedes the ability of MAR models to describe stochastic structures in the data, but this aspect is not the focus of the present study. Smoothing is often used to reduce stochastic features affecting the data, and while it can be very helpful, one must be aware that it might also obscure deterministic features, thereby yielding misleading results (Supplementary Figure S11).

We begin with a description and comparison of the main features of LV and MAR models, subsequently analyze small synthetic systems, which offer the advantage of simplicity and full knowledge of all model features, and then assess several real-world systems. It is quite evident that it is impossible to compare distinct mathematical approaches with absolute objectivity and without bias (Rykiel, 1996), and it sometimes happens that inferior choices of models in specific cases outperform otherwise superior alternatives. We will attempt to counteract these vagaries by selecting case studies we consider representative and by stating positive and negative facts and features as objectively as possible.

## 2 Models and methods of analysis

### 2.1 Lotka-Volterra models

Lotka-Volterra (LV) models (Lotka, 1925; Volterra, 1926) are systems of first-order ordinary differential equations (ODEs) with the format

$$\frac{dX_{i}}{dt}=a_{i}X_{i}+\overset{n}{\underset{j=1}{\sum }}b_{ij}X_{i}X_{j},i=1,2,\ldots ,n$$ (1)

The left side of Eq.1 represents the change in species $X_{i}$ with respect to time. With only the first term on the right side, $a_{i}X_{i}$ , the equation yields exponential growth, while the sum captures interactions between pairs of populations. Most of these terms represent interactions between different species, such as predation or competition for the same resources or cooperation, but one term in each equation, $b_{ii}X_{i}X_{i}$ , accounts for interactions among the members of the same species and is sometimes interpreted as a *crowding effect*.

If time-dependent environmental inputs are to be considered, one may add one or more terms $\gamma_{ik}X_{i}U_{k}$ , where $U_{k}$ is the *k*th element of a vector of these inputs and the coefficients $\gamma_{ik}$ are weights that quantify the effects of the factors on species *X*_*i*_ (Stein et al., 2013; Dam et al., 2016, 2020). This addition does not fundamentally alter the format of Eq. 1:

$$\frac{dX_{i}}{dt}=a_{i}X_{i}+\overset{n}{\underset{j=1}{\sum }}b_{ij}X_{i}X_{j}+\overset{m}{\underset{k=1}{\sum }}\gamma_{ik}X_{i}U_{k},i=1,2,\ldots ,n$$ (2)

In an effort to simplify the comparisons in this study, these environmental factors will be omitted henceforth, both in LV and MAR.

Background and further details regarding these models are presented in Supplementary Section S1.1. Because ODEs are natural representations of dynamic processes, explicit mention of time, *t*, is omitted.

### 2.2 Estimation of LV parameters based on slopes of time courses

Any of the numerous generic parameter estimation approaches for systems of non-linear ODEs may be used to estimate the parameter values of LV systems; reviews include (Mendes and Kell, 1998; Wedelin and Gennemark, 2007; Chou and Voit, 2009). Here, we use a combination of smoothing, slope estimation, and parameter inference, for which we use the recently introduced, very effective Algebraic Lotka-Volterra Inference (ALVI) method (Voit et al., 2021). We begin by smoothing the raw time series to reduce noise in the data as well as in their slopes, where the effects of noise are usually exacerbated (Knowles and Renka, 2014). Many options are available, but smoothing splines and local regression methods are particularly useful (Cleveland, 1981); they are reviewed in Supplementary Section S1.2.1. Splines have degrees of freedom and we will refer to a spline with, say, 8 degrees of freedom as “8DF-spline”.

The estimation of slopes is a preliminary step for converting the inference problem from one involving ODEs into one exclusively using algebraic functions (Varah, 1982; Voit and Savageau, 1982; Voit and Almeida, 2004); see also Supplementary Sections S1.2.2, 1.2.3). For this task, we have two options: we may estimate slope values either at time points corresponding to the measured data points or for a sample of many points of the smoothing function, which yields a larger set of numerical values for variables and slopes (Voit and Almeida, 2004). The next step of this conversion is accomplished by substituting the left side of Eq.1 for each variable *X*_*i*_ at *K* time points with the estimated slopes. These slope values are equated to the right-hand side of the equation with values of the dependent variables at the same *K* time points. This conversion of one ODE into *K* algebraic equations leaves the parameters as the only unknowns that are to be estimated.

After the differentials are replaced with estimated slopes, two options permit the inference of the parameter values of LV-models. We can apply simple multivariate linear regression (ALVI-LR), where we either use all data points or iterate the regression several times with subsets of points, which is a natural means of creating ensembles of solutions. As an alternative, if *n* is the number of variables, one may use *n*+1 of the data points and slopes, which results in a system of linear equations that can be solved with simple algebraic matrix inversion (ALVI-MI). For a thorough description of these algebraic methods see (Voit et al., 2021) and an example in Supplementary Section S1.2.5.

### 2.3 Multivariate Autoregressive (MAR) models

In contrast to the ODEs of the LV format, Multivariate Autoregressive (MAR) models are discrete recursive linear models (Ives et al., 2003; Holmes et al., 2012). They have the general format

$$X_{i,t+1}=\alpha_{i}+\overset{n}{\underset{j=1}{\sum }}\beta_{ij}X_{j,t}+\overset{m}{\underset{g=1}{\sum }}\gamma_{ig}u_{g,t}+w_{i,t};\;i=1,2,\ldots ,n;w_{i,t}∼N\left(0,\delta_{i}\right)$$ (3)

In this formulation, the quantities *u*_*g*,t_ represent environmental variables and the noise *w*_*i*,t_ is normally distributed. Expressed in words, the “state” of the system at time *t*+1, represented by the vector *X*_*t*+1_, depends exclusively on the state of system one time unit earlier, *X*_*t*_, as well as on external inputs and stochastic effects.

This set of equations, for all *i*, is usual represented in the matrix form as

$$X_{t+1}=\alpha +\beta X_{t}+\gamma u_{t}+w_{t},w_{t}∼MVN\left(0,\delta \right)$$ (4)

*α* is the vector of intersects and *β* is the population interaction matrix. The term $\gamma u_{t}$ describes how cofactors affect the dependent variables. Specifically, *u*_*t*_ is a vector of external variables and *γ* is the matrix of weights associated with these external variables. Finally, the term $w_{t}$ is a vector representing stochastic noise affecting the dependent variables.

Eq. 4 conveys that the state of the system at time *t*+1 depends on the state at *t* and possibly on temporary environmental and/or other stochastic input. As an alternative to this modeling structure with “memory 1,” it is possible to extend MAR models to depend also on states farther in the past, such as *X*_*t*-1_, *X*_*t*-2_, and *X*_*t*-3_, in addition to *X*_*t*_. However, this strategy greatly increases the number of parameters to be estimated, and the commonly used models depend only on the immediately prior state; they are sometimes called MAR(1). Here, we only consider MAR(1) models and refer to them simply as MAR models.

MAR models can be interpreted in two distinct ways. In generic mathematical terms, MAR models are stochastic, linear approximations of non-linear dynamic systems that evolve over time in the vicinity of a fixed point (steady state). According to this interpretation (Holmes et al., 2020), *x*_*t*_ is a vector of the realization of random variables at time *t*. The “noise” actually captures natural variations in environmental conditions, as well as measurement inaccuracies, and is modeled by a multivariate normal distribution with mean zero and variance-covariance matrix *δ*. Obviously, other distributions could be employed, but the multivariate normal is the one typically chosen by practitioners in the field (Ives, 1995; Certain et al., 2018). If stochasticity is omitted, MAR models exhibit quite a bit of similarities with LV models, as long as they operate close to the steady state or are only mildly non-linear (see Supplementary Section S1.3; Section 2.5 below).

One may also interpret MAR models using ecological arguments. Specifically, they can be viewed as multispecies competition models with Gompertz density dependence, where instantaneous growth rates decrease linearly over time as the population sizes increase (Ives, 1995; Certain et al., 2018). In this view, *x*_*t*_ is a vector of the log-abundances of dependent variables at time *t*. Further details regarding these models are presented in Supplementary Section S1.3.

One should note that the incorporation of environmental variations in a deterministic model may change the interaction structure of a community (Chesson, 2020; Hawlena et al., 2022), both in the short and the long term. For example, the number of species able to coexist can increase if temporal environmental variations cause fluctuations in resource uptake, as it can be the case for nocturnal and diurnal species that live in the same habitat and consume similar resources. Another example is a mixed bacterial community, whose interaction structure can significantly change if it is exposed to an antibiotic (Varga et al., 2022). From a biological point of view, these effects may not be surprising, but it is difficult to propose a general mathematical solution, unless the nature and quantitative details of the alterations can be converted into fully characterized functions affecting the parameters. Non-etheless, even if a precise mathematical formulation is not feasible, these considerations should not be ignored.

### 2.4 Parameter estimation for MAR models

The software package MARSS, using an expectation maximization algorithm, greatly facilitates the estimation of MAR model parameters (Holmes et al., 2020, 2012). Some details of MARSS usage, and especially the setup we used, are discussed in Supplementary Section S1.5.

### 2.5 Structural similarities between the two modeling formats

Both LV and MAR models have been proposed as effective tools for characterizing the interactions among populations within dynamically changing mixed communities. At first glance, the two formats appear to be distinctly different and, in a strict sense, incomparable. However, they do exhibit fundamental mathematical similarities, which are sketched below and analyzed in more detailed in Supplementary Section S1.4.

To assess these similarities, we focus on MAR models without environmental factors and noise, i.e.,

$$X_{i,t+1}=\alpha_{i}+\overset{n}{\underset{j=1}{\sum }}\beta_{ij}X_{j,t};i=1,2,\ldots ,n;$$ (5)

(Dennis and Taper, 1994; Ives, 1995; Certain et al., 2018). Borrowing the principles of solving ODEs with Euler’s method, we discretize the LV model (Eq. 1), which yields

$$X_{i,t+h}\approx X_{i,t}+h\cdot {\frac{dX_{i}}{dt}}_{X_{i}=X_{i,t}}=X_{i,t}+h\cdot X_{i,t}\left(a_{i}+\overset{n}{\underset{j=1}{\sum }}b_{ij}X_{j,t}\right),i=1,2,\ldots ,n$$ (6)

The left quasi-equality in this formulation can be interpreted as a linearization of the time evolution of the LV dynamics in Euler’s sense, while the right equality still exhibits the genuine non-linearity of the LV model. Generically, linearization is a common tool for developing a better understanding of population fluctuations in ecology (Ripa and Ives, 2003). It is usually performed at the steady state for mathematical analyses of non-linear ODE system, such as stability and sensitivity assessments (Voit, 2017). Here, we employ Euler’s stepwise-linearized formulation of the system dynamics solely as a means of discretizing the ODE format of LV in order to compare it more directly with the MAR structure.

For simplicity of discussion, suppose *h* = 1. If the dynamics remains close to the steady state, then $X_{i,t+1}-X_{i,t}\approx 0$ for any given *t*. Furthermore, division of both sides of (6) by $X_{i,t}$ , as long as it is greater than 0, yields approximately one on the left-hand side and a linear expression on the right-hand side. Thus, the results corresponding to Eqs 5, 6, respectively, close to the steady state are

$$\alpha_{i}+\overset{n}{\underset{j=1}{\sum }}\beta_{ij}X_{j,t}-X_{i,t}\approx 0$$ (7)

and

$$a_{i}+\overset{n}{\underset{j=1}{\sum }}{\tilde{b}}_{ij}X_{j,t}\approx 0$$ (8)

respectively. The two sets of near-steady-state conditions 7) and 8) are the same if *α*_*i*_ *= a*_*i*_, *β*_*ij*_ *= b*_*ij*_ for all *i ≠ j* and *β*_*ij*_ *=* ${\tilde{b}}_{ii}=$ *b*_*ij*_ + 1 for *i* = *j*. Thus, in spite of their apparent differences, the basic MAR and LV models have the same steady-state equations. Their dynamics is not addressed here but it is usually expected to be similar if it stays close to this steady state. Comparing the two models in this manner is useful because the different formats highlight the strengths and weaknesses of the two methods. In MAR, noise is an aspect of the model that can be estimated and taken into consideration for the estimation of the interaction parameters (Certain et al., 2018). Noise is not considered in LV models. Furthermore, MAR is linear and uses a log transformation to deal with some non-linearities. In LV, multiplying $X_{i,t}$ by the growth rate and sum of interaction terms allows for non-linearities and does not require any further remediation to handle them.

## 3 Results

The comparison between LV and MAR models may be executed in two ways. A purely mathematical approach was sketched in Section 2.5 and expanded in Supplementary Section S1.4. An alternative approach focuses on practical considerations and actual results of inferences from data. It is described in this section.

For simplicity, we omit environmental inputs (*γ*_*ik*_ *X*_*i*_ *U*_*k*_ and *γ*_*ig*_ *u*_*g,t*_, respectively) and begin by testing several synthetic datasets with different types of representative dynamics. We design these data as moderately sparse and noisy, to mimic reality. In particular, we test whether the LV inference from synthetic LV data returns the correct interaction parameters and whether the MAR inference from synthetic MAR data does the same. Subsequently, we test to what degree LV inferences from MAR data yield reasonable results and *vice versa*. Finally, we apply the inferences to several real datasets from the literature. As the main metric, we compare the sums of squared errors (SSEs) and use a Wilcoxon rank test to assess the significance of the differences.

### 3.1 Case study 1: Synthetic LV data

The first case study addresses data that were generated with a synthetic four-variable LV model with what is called process noise (de Valpine and Hastings, 2002; Fiasconaro et al., 2004). In contrast to observational noise, which is due to uncertainties during the data acquisition, process noise is not truly “noise” in ecological systems, and the terminology is therefore misguided. Instead, it is the manifestation of temporary environmental variations that are natural and can be very influential for the functioning of ecological systems. The nature of process noise mandates that we do not solve the ODE system and then superimpose all points of the solution with observational noise, as it is usually done, but allow noise to affect the system in a repeated fashion during sequential steps of the temporal evolution of the system. This gradually accumulating type of noise, that is, the overall effect of temporary environmental variations, appears to be closer to reality and actually aligns better with MAR models (for details, see Supplementary Section S2 and *Discussion*). The specific question we address here is whether and with what degree of accuracy the LV and MAR inference methods return the true dynamics and parameter values, which are all known by design. Because it is difficult to embed repeated stochastic inputs in ODE models, we discretize the LV model, which does not compromise its richness; as an analogous conversion of ODEs in a biochemical context, see (Voit and Olivença, 2022).

For a representative illustration of the parameter inference process in the presence of stochastic environmental variations, we begin with the four-variable LV system

$$\frac{dX_{i}}{dt}=X_{i,t}\left(a_{i}+\overset{4}{\underset{j=1}{\sum }}b_{ij}X_{j,t}\right),i=1,\ldots ,4$$ (9)

Here, the variables *X*_*i*_ and *X*_*j*_ represent the abundances of the different species, *a*_*i*_ the rate constants and *b*_*ij*_ the intra- and interspecies interaction parameters. To account for stochastic environmental variations, we use a discretized LV system, which yields

$$X_{i,t+h}=\left(X_{i,t}+h\cdot X_{i,t}\left(a_{i}+\overset{4}{\underset{j=1}{\sum }}b_{ij}X_{j,t}\right)\right)*gamma\left(k_{i},1/\left(k_{i}-1\right)\right),i=1,\ldots ,4$$ (10)

Here, *X*_*i*_, *X*_*j,*_ *a*_*i*_ and *b*_*ij*_ have the same meaning as in Eq. 9 and the *k*_*i*_ represent the shape parameter for the gamma-distributed influences affecting the four variables. The scale parameter is set to $1/\left(k_{i}-1\right)$ for the mode of the gamma to equal 1.

**FIGURE 1:**
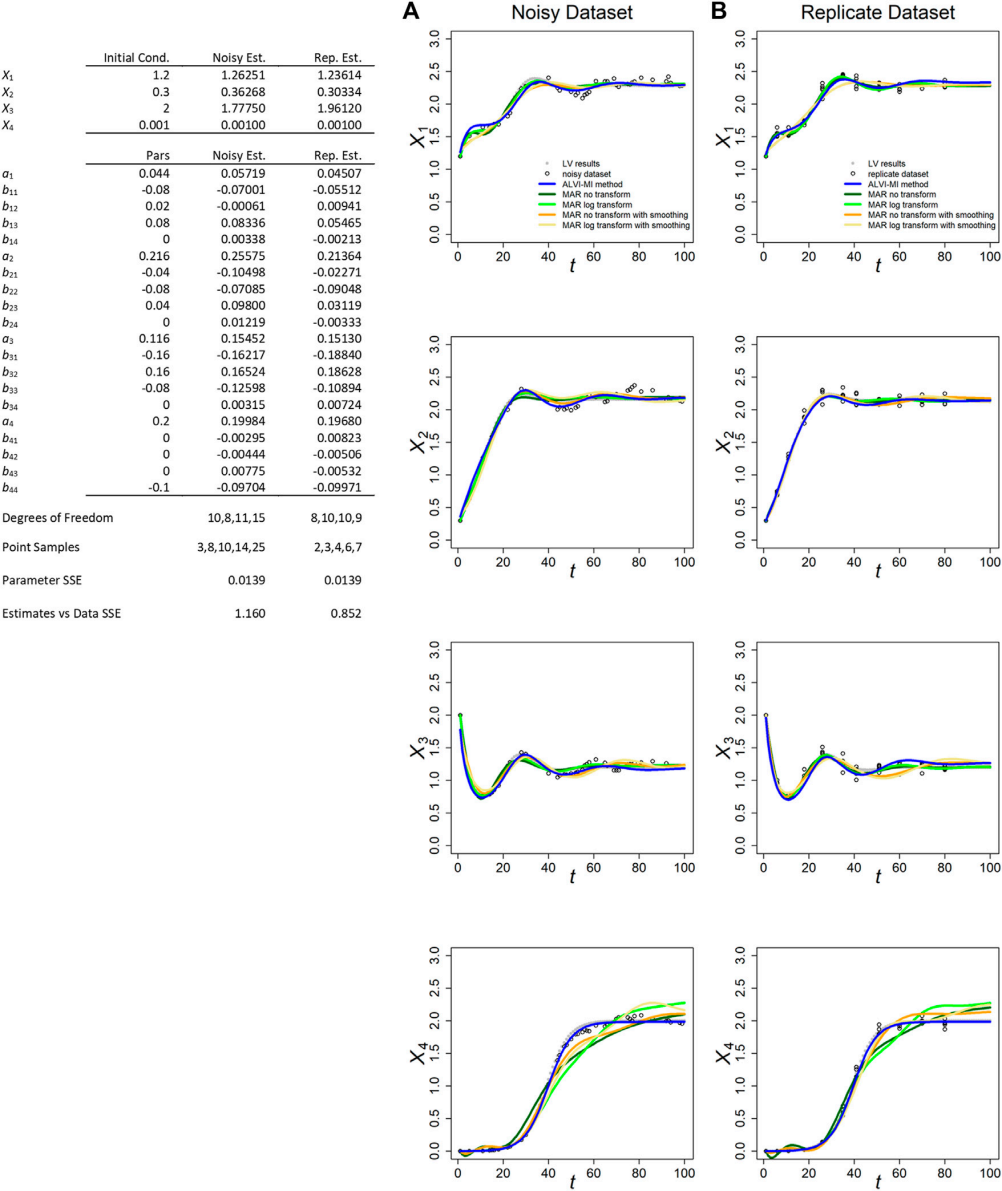
ALVI-MI and MARSS methods applied to noisy **(A)** and replicate **(B)** LV datasets with process noise. Original synthetic data are shown as gray dots and data with added noise as black circles. LV results are presented in blue. True parameters and LV estimates are presented in the Table. MAR estimates are presented in green, orange and yellow. Data and parameter estimates for MAR can be seen in Supplementary Table S1. SSEs for all fits are presented in Table 1.

The parameters for the illustration are presented in Supplementary Figure S1, and a time series of the dynamics of this system is shown in Supplementary Table S1.1 and as circles in Figure 1; Supplementary Figure S1. For the special case of noise-free data, the inferences are close to perfect with respect to the trajectories and parameter values (Supplementary Figure S2). To mimic a more realistic scenario, we created a noisy dataset, visualized in Supplementary Figure S1A, which was constructed by permitting stochastic variations to the system and randomly choosing forty points. The “process noise” was set as a gamma random variable parametrized as explained before with the shape parameter *k* equal to 10,000. For all practical purposes, the high value for *k* makes the gamma almost identical to a normal with mean one and standard deviation of 0.005, but it generates only positive values. This noise appears to be small but quickly accumulates, as it affects every step of the solution. This noisy dataset is shown in Supplementary Table S1.2.

As a second realistic situation, we constructed a dataset with replicated measurements (Supplementary Figure S1B), which was accomplished by first choosing fifteen time points from the data that characterize the dynamic (including extremes values). Next, we generated time series of the system with environmental variations and recorded the values of the variables at the previously chosen time points. This process was iterated to create five replicates per chosen point. This replicate dataset is shown in Supplementary Table S1.3.

Variable *X*_4_ was intentionally designed as a (decoupled) logistic function. It is unaffected by the other variables and does not affect them either. It was included to explore to what degree the methods to be tested can detect this detachment.

The fits for the noisy and replicate LV datasets, obtained per LV matrix inversion, are presented in Figures 1A,B, along with the inferred parameter estimates (Table in Figure 1). These generally possess the correct sign and could, if deemed beneficial, serve as the starting point for an additional, refining optimization, for instance with a steepest-descent method. The inferred and true values are quite similar for both datasets. Because we usually obtain better results through matrix inversion, we display those results here and present linear LV regression results in the Supplements.

Figure 1 also displays the MARSS estimates with and without log-transformation of the data and with or without data smoothing. With respect to the smoothed trends of the noisy LV dataset, the MAR estimates without smoothing consistently yield slightly lower SSEs for *X*_1_, *X*_2,_ and *X*_3_ than the LV inferences while, surprisingly, MAR with smoothing yields similar results. For the replicate LV dataset, the SSEs for all methods are similar, although MAR with smoothing produces slightly worse results (Supplementary Table S1.6). The parameters for *X*_4_ are consistently better represented by LV. Overall, the results for *X*_1_, *X*_2,_ and *X*_3_ are quite similar, and all LV and MAR fits capture the dynamics of these three variables very well. In fact, given how similar the SSEs are, it is quite possible that other simulated data with noise would result in smaller SSEs for the LV inference (see later example of inferences of parameter values to noisy data yielding better fits than the correct parameter values). A possible reason for MAR yielding smaller SSEs for *X*_1_, *X*_2,_ and *X*_3_ seems to be that the addition of process noise tends to introduce spurious oscillations that are captured by the smoothing splines used for the LV inferences, as can be seen in Supplementary Figure S6. Of course, we could use splines that further smooth the data but doing so would risk the loss of true dynamic features in the data, as can be seen in Supplementary Figure S3.

MARSS did not perform well for the “decoupled” variable *X*_4_, in either of the two datasets and for all variations of the MAR method. Indeed, the poor performance for *X*_4_ rendered the overall final SSE score for all MAR variants worse than for LV (Supplementary Table S1.6). The most likely reason is probably the fact that this variable starts far away from the steady state and is highly non-linear, with is at odds with the MAR structure. It could also be that the algorithm used in MARSS has problems estimating parameters with a true value of zero. Holmes et al. (2020) reported this issue for the diagonals of R and Q matrices, although not for other parameters. LV outperformed MAR in the case of data if observational noise was analyzed (Supplementary Table S2.5).

Because MARSS yields parameter values for a discrete recursive system, they are not directly comparable to the true parameters of a LV system; nonetheless, their numerical values are recorded for completeness in Supplementary Tables S1.4, S1.5. For MARSS inferences from the replicate LV dataset, we had to average points with the same time value. Additional details are presented in Supplementary Section S2.

In both LV datasets, the matrix inversion method produced parameters estimates closer to the true parameters (Supplementary Table S8).

We also studied an example similar to the one discussed in this section and presented in Figure 1, but with a high standard deviation for the process noise (0.03 instead of 0.005). With the higher standard deviation, the dynamic deviated considerably from the original and all methods showed decrease in accuracy in capturing it. In the noisy dataset, MAR without any transformation performed better followed by ALVI-MI (Supplementary Table S1.7). Of note, all methods performed considerably better in the replicate dataset, suggesting that this type of sampling may be an appropriate method to capture the true dynamics in situations affected by high process noise. This can be seen in Supplementary Figure S10 and discussed in the last paragraphs of Supplementary Section S2.

### 3.2 Case study 2: Synthetic MAR data

Here we reverse the set-up of Case Study one by creating synthetic data with an MAR model and test whether inferences with either model can achieve results corresponding to the original system. One could argue that data in the real world very seldom result from truly linear processes, but it is nevertheless important to analyze linear MAR models because practitioners within the ecological community have been using them.

**FIGURE 2:**
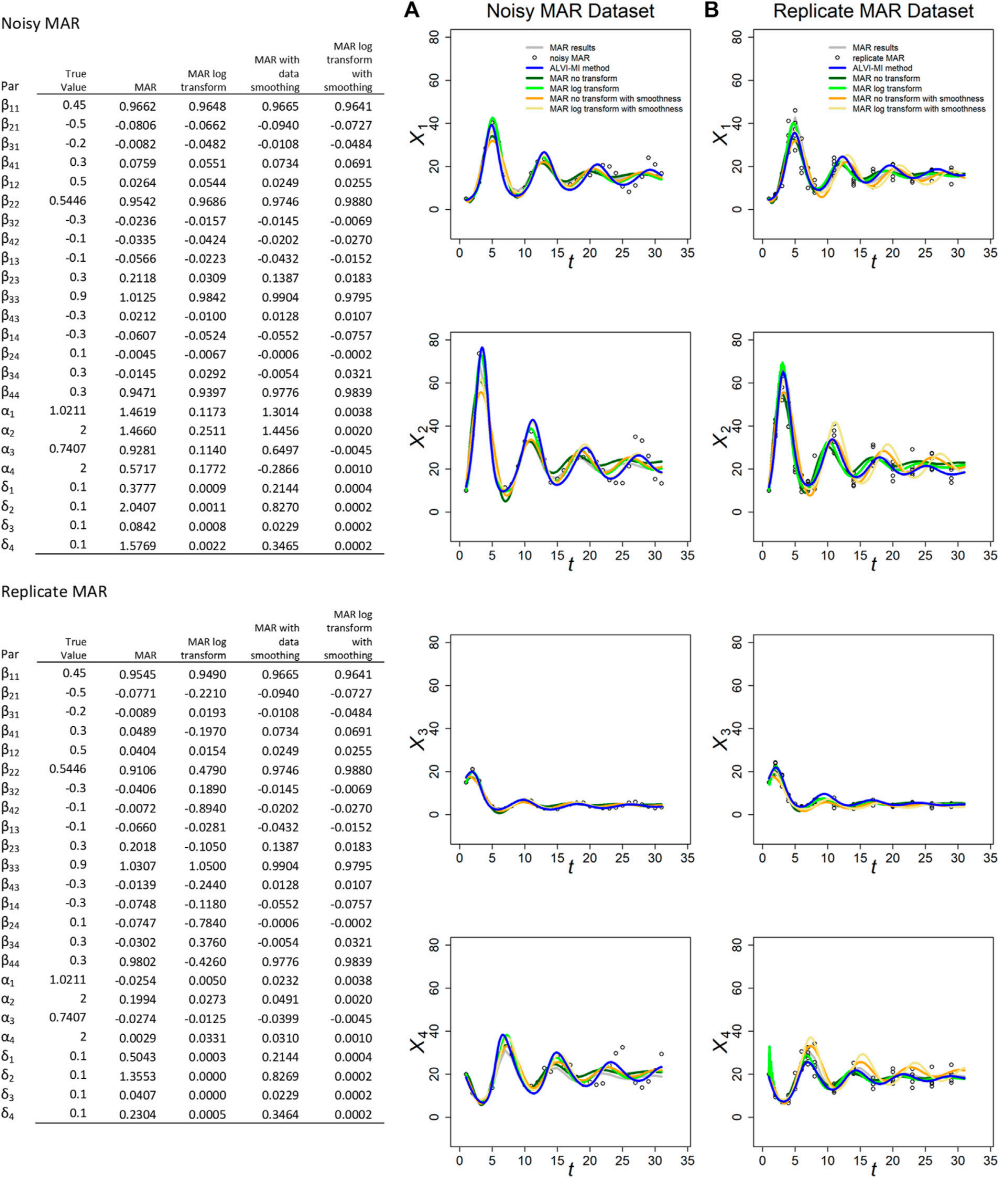
MARSS and ALVI-MI methods applied to noisy **(A)** and replicate **(B)** MAR datasets with process noise. Original synthetic data are shown as gray dots, data with added noise as black circles. MAR estimates are presented in green, orange and yellow. LV estimates are in blue. The variables of the noisy dataset were smoothed with 15DF-splines and the LV solution was calculated with spline points at times 2, 4, 7, 22, and 27. The variables of the replicate dataset were smoothed with 11DF-splines and the LV solution was calculated with spline points corresponding to times 2, 3, 4, 5 and 12. Data and parameter estimates for LV can be seen in Supplementary Table S5. SSEs for these fits are presented in Table 1.

As a representative example, we use a four-variable MAR system to generate 31 synthetic datapoints. We create a noisy *MAR* dataset with process noise by using the logarithm of the data and consider the MAR model again as a multispecies Gompertz competition model (Ives, 1995; Certain et al., 2018). We also create a replicate *MAR* dataset by choosing 15 time points and harvesting them in five time series of the process. The initial conditions and parameters are presented in Supplementary Table S5, dynamics, LV and MAR fits are presented in Figure 2. All fits to the synthetic MAR data, with either method, are satisfactory. Due to the different nature of the two modeling formats, the LV parameters are not directly comparable to the MAR parameters.

MAR fits and SSEs are clearly superior to LV for the noisy MAR dataset, but that is not the case for the replicate MAR dataset, where ALVI-MI produces the lowest SSE (Table 1). For the MAR variants, MAR with log transformation and without smoothing yields the lower SSEs. This result highlights the importance of the log-transformation to accommodate non-linearities with MAR. In most cases, the different MAR models had difficulties retrieving the true parameters of the system, and sometimes even the correct sign (Figure 2). This finding is probably due to the small number of datapoints: Certain et al. (2018) suggest that the length of the time series should be at least 5 times greater than the number of *a priori* non-zero elements in the matrix *β* in order to recover interaction signs correctly. Our sample has 31 observations but, according to this criterium, should have at least 80. For practical inference purposes in biology, this requirement regarding the density of data can be a genuine concern.

**TABLE 1:** Sum of squared errors (SSE) of data fits for all experiments with ALVI-LR (linear regression), ALVI-MI (matrix inversion) and four variants of the MAR methods. We also include SSEs for the estimates obtained by Mühlbauer et al. (2020) for LV data presented in Figure 4. Bold values identify the lowest SSE score for each example. Examples used in the Wilcoxon rank test are marked with asterisks.

	Shown in	ALVI-LR	ALVI-MI	MAR	MAR logTrans	MAR with smoothing	MAR with log and smoothing	Mühlbauer et al	Test
Noisy LV data	Figure 1A	**1.076**	1.160	3.674	5.335	2.638	5.017		*
Replicate LV data	Figure 1B	0.349	**0.226**	1.513	1.395	1.394	1.481		*
Noisy MAR dataset	Figure 2A	1360.438	1221.929	636.272	**381.430**	830.475	1141.293		*
Replicate MAR dataset	Figure 2B	754.249	**385.279**	1144.325	454.437	1070.461	583.539		*
Synthetic Data 1	Supplementary Figure S5A	**3.29E-07**	9.04E-07	10.493	4.626				
Synthetic Data 2	Supplementary Figure S5B	8.68E-06	**8.57E-06**	3.029	5.876				
Synthetic Data 3	Supplementary Figure S5C	**0.002**	0.018	5.111	4.378				
Synthetic Data 4	Supplementary Figure S5D	**5.65E-04**	2.10E-02	2.47E+04	3.99E+35				
Synthetic Data 5	Supplementary Figure S5E	**7.048**	18.939	16.588	15.695				
Synthetic Data 6	Supplementary Figure S5F	71.271	**28.357**	7379825.00	174.247				
Mühlbauer et al. 1	Figure 3A	2588.491	**2515.934**	18159.950	51738.263	17876.615	16398.792	3271.035	*
Mühlbauer et al. 2	Figure 3B	6203.687	**4781.476**	13016.262	22162.242	9292.995	12446.589	40603.67	*
Mühlbauer et al. 3	Figure 3C	24182.900	**2190.5862**	4618.7096	10146.0397	4779.6096	8795.03	**578.092**	*
Mühlbauer et al. 4	Figure 3D	17,700,169	**1,118,350**	2,199,174	1,777,559	9,904,281	3,973,876	7,934,136	*
Mühlbauer et al. 5	Figure 3E	12,114,811	**2,251,804**	4,245,588	4,450,871	5,596,187	4,554,089	2,893,764	*
Holmes et al. 1	Figure 3A	16,8134,153	153,703,251	217,478,452	166,186,993	**143,728,190**	212,424,910		*
Holmes et al. 2	Figure 3B	32,326,913	**5,556,585**	10,267,681	10,947,294	11,136,306	14,342,449		*

We used data without noise to calculate the final SSEs and determine which method captures the process best. For everything else including selecting the best parameter sets, we used the MAR data with noise. One should note that ALVI fits may be judged differently in quality if the SSE is computed either with respect the smoothed trends or the raw data. In fact, ALVI-MI could be set with different configurations, which might give a lower final SSE against the smoothed data but would have a higher SSE against the noisy data. As an example, using again the noisy MAR dataset, consider splines for four variables with 20, 15, 15, 12 degrees of freedom, respectively, and a subsample consisting of the second, fourth, seventh, 22nd, and 28th datapoints. With these settings, we obtain an SSE of 1,530 against the noisy data themselves but an SSE of 600, when measured against the smoothed trend. With a different setting, the SSE against the noisy data is about 1,360 (Table 1).

For the noisy MAR dataset, the MAR parameter estimates without transformation or smoothing worked best and yielded the closest parameter estimates to the true parameters (Figure 2).

### 3.3 Inference of complex dynamics

**FIGURE 3:**
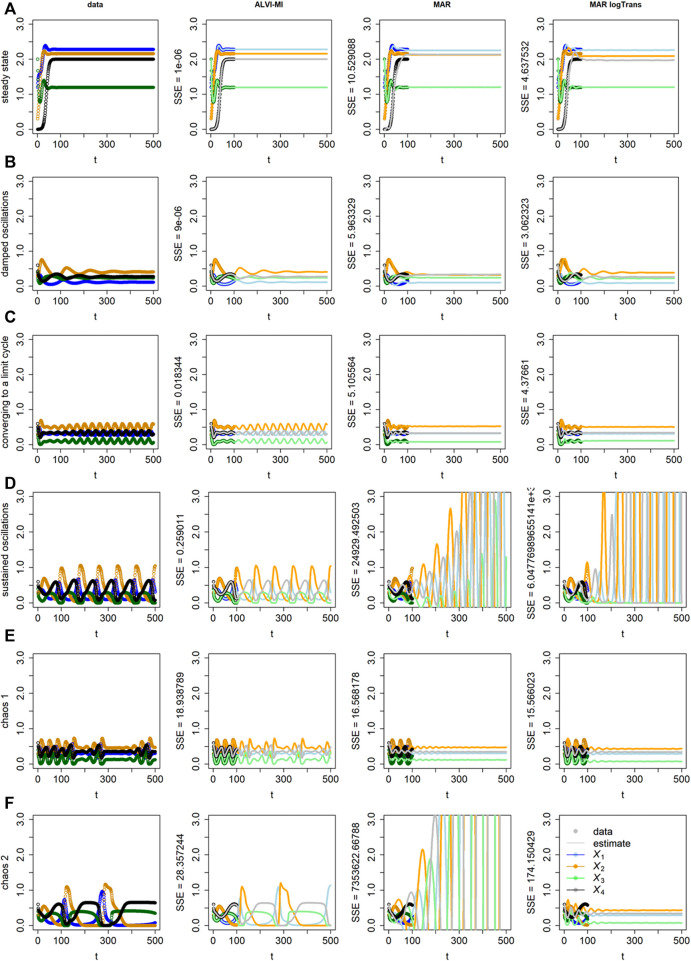
“Data” (Column 1) and results of inferences with ALVI-MI (Column 2) and MAR methods (Columns 3 and 4) for LV systems exhibiting different types of increasingly complex dynamics. Thick lines in Columns 2–4 correspond to the time period from which the data were sampled, while thin lines are extrapolations in time. Row **(A)** Data converging to a stable steady state; Row **(B)** Damped oscillations; Row **(C)** Initially erratic oscillations converging to a limit cycle; Row **(D)** Sustained oscillations; Row **(E)** Deterministic chaos, example 1; Row **(F)** Deterministic chaos, example 2. Data, ALVI-MI and MARSS estimates are presented in Supplementary Table S3. The SSEs concerning the differences between the data and estimates for *t* ϵ [1, 500] are presented as labels to the *Y*-axis. No smoothing was needed because the data were noise free.

ALVI also works for more complicated dynamics than analyzed so far, as can be seen in Figure 3. Here we are interested in determining if the methods can recover the dynamics, which in some cases turns out to be challenging for sparse data even without the introduction of noise. Thus, we used the synthetic data unaltered. Specifically, data for early time points (*t* ∈ [1, 100]) were fitted and then extrapolated for a much longer time horizon of *t* ∈ [1, 500]. In these examples, ALVI-MI is used with 100DF-splines. It uses data samples with points corresponding to timepoints *t* = 5, 10, 20, 30, and 50 for all cases except for the chaotic oscillations where we used *t* = 4, 6, 10, 15, and 35. For each case, we also present the MAR estimates. In all fairness, one must recall that the original data were produced with LV models. While the MAR model extrapolations are not always satisfactory, it is nevertheless comforting that the inference method returns good results for the short initial time interval used for data fitting.

ALVI-MI generally performed very well but did not adequately capture the deterministic chaos (chaos 1), which is understandable as chaotic systems are extremely sensitive to any type of numerical variation. For this case only, we obtained a better fit using ALVI-LR. Apart from this situation, results with ALVI-LR are very similar to ALVI-MI results and therefore not displayed.

For the data in Figure 3B, the MAR model performed well when log-abundances were used. In the remaining cases, it failed to replicate the oscillations, or these exploded by reaching amplitudes far bigger than in the dataset. One also notes early discrepancies between the initial points used to create the estimates and the MAR estimates.

### 3.4 Case study 3: Experimental data from the literature

#### 3.4.1 Published LV inferences

**FIGURE 4:**
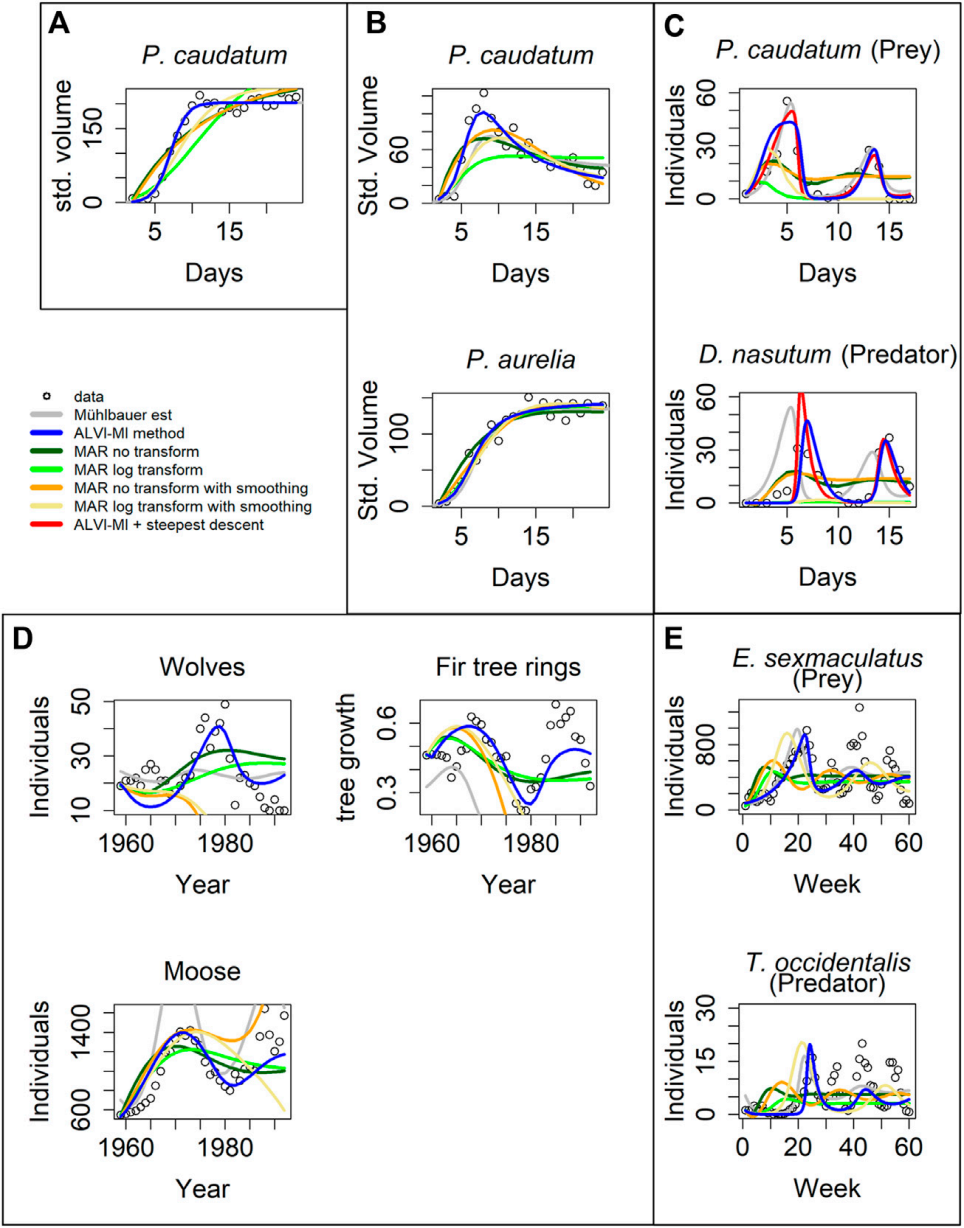
Model inferences associated with Gause’s data (Gause, 1934). **(A)** Standardized volume of Paramecium caudatum grown in monoculture. **(B)** Standardized volume of Paramecium caudatum and Paramecium aurelia grown in mixed population. **(C)** Predator-prey interactions between Didinium nasutum and Paramecium caudatum grown in mixture. **(D)** Multi-trophic dynamics for wolves, moose, and fir trees on Isle Royale from 1960 to 1994. **(E)** Predator-prey interactions between Eotetranychus sexmaculatus and Typhlodromus occidentalis. Circles show observations, gray lines are estimates from Mühlbauer et al. (2020). LV estimates are presented as blue lines and MAR estimates as green, orange and yellow lines. Red lines in **(C)** correspond to a steepest descent optimization using the solution of ALVI-MI as initial guess. See Text and Supplementary Tables S4.1, S4.3 for further details.

Data from Georgy Gause’s experiments in the 1930s and others were recently compiled in the R package gauseR (Mühlbauer et al., 2020). In the accompanying paper, the authors present five examples to test their method for estimating LV model parameters. We use the exact same examples to demonstrate to what degree LV and MAR methods are compatible with these real-world data and compare our results to those presented by Mühlbauer and colleagues*.* For more information regarding the original experimental data, see (Gause, 1934; Huffaker, 1958; McLaren and Peterson, 1994; Mühlbauer et al., 2020). The results are presented in Figure 4, with data as circles and various estimates as lines. SSEs of the different estimates for these and other test examples are presented in Table 1.

The overall result is that the inferred MAR models never outperform the results for the corresponding LV models. Details are provided below. One could argue that these examples had been used to test actual data for compatibility with the LV structure, which may explain the superior performance of the LV model. However, these are actual, real-world data of the type that both LV and MAR are supposed to capture.

For the case in Figure 4A, matrix inversion with the LV model yields the same results as found in (Mühlbauer et al., 2020). In contrast, the MAR estimates are poor, with a very high estimate for the noise (Supplementary Table S4.3), especially if one does not use log-abundances; this problem occurs for all cases presented in Figure 4. The data in Figure 4A are close to a logistic function, similar to *X*_4_ in the previous noisy dataset, where MAR also did not perform well.

Figure 4B shows data from a competition experiment between the unicellular protists *Paramecium caudatum* and *Paramecium aurelia* that were co-cultured. Estimates for *P. aurelia* are similar for all methods but LV matrix inversion exhibits clear superiority for *P. caudatum*.

The data in Figure 4C are complicated. Mühlbauer and colleagues noted that additional quantities of protists were introduced to avoid species extinction. Furthermore, many datapoints in this dataset are zero, which causes problems for the parameter estimators. As a remedy, we changed the zeros to 10^−5^, but our initial estimates still produced poor fits. However, if we use the estimated trajectories from Mühlbauer et al. (2020) as “data,” quasi as a diagnostic measure, matrix inversion captures the parameters that reproduce the fit of Mühlbauer et al. (2020) very well. This finding suggests that the initial poor fit is not a problem of LV adequacy. Instead, we hypothesize that the problem is caused by insufficient datapoints or almost-linear dependence, which affects the matrix inversion. To test this hypothesis, we used the first splines as data to create a second set of splines that has more datapoints to create the subsample to be used for the LV matrix inversion. We were able to achieve the presented fit, which is still somewhat inferior to the one by Mühlbauer et al. (2020), but constitutes a considerable improvement over our initial fits. Furthermore, using the solution from the matrix inversion as initial parameter values for a subsequent gradient-descent optimization, the resulting solution reflected the data well, with a better SSE than all other methods.

When calculating splines for this dataset, it is difficult to choose degrees of freedom that capture both maxima. High degrees of freedom capture the global maxima but overshoot the local maxima. Low degrees of freedom capture the local but undershoot the global maxima. We suspect this to be the cause for the initially poor performance of LV. Still, LV yields better fits than MAR.

The data in Figure 4D are also complicated, in this case due to two aspects. First, they show a stark difference in absolute numbers, with the abundance values for moose being several magnitudes higher than the numbers of tree rings. As a potential remedy, we normalized the fitting error for each dependent variable by dividing it by its mean to balance the SSE. The result is shown in Figure 4D. The LV models perform better than MAR, and MAR with log-abundances produces a better noise estimate than with the untransformed data.

The second issue is the fact that, around 1980, the wolves were exposed to a disease introduced by dogs, which caused a precipitous drop in the wolf population between 1981 and 1982 (Park Service, 2021). Typical mathematical models are not equipped to simulate such a black swan event, and the totality of results from the various methods suggests that neither LV nor MAR may be good models for this system, because none of the fits, by Mühlbauer et al. (2020), LV, or MAR, are entirely satisfactory. Non-etheless, our LV results present a decent fit for moose and fir tree rings. To improve the fit to the wolf data, we divided the data into two groups, from 1959 to 1980 and from 1983 to the end of the series and estimated parameters for the two intervals. The results are presented in Supplementary Figure S8 in red lines. The fit is greatly improved, although still not perfect.

Figure 4E describes yet another complicated example. According to the inference, the LV estimates fit the first peak well but the oscillations die down, in contrast to the data. Estimates from Mühlbauer et al. (2020) produce even poorer estimates, suggesting that the data may not be compliant with the LV structure. As in the previous example, MAR models do not capture the dynamics, although MAR with log-abundances produces good noise estimates. Surprisingly, MAR with smoothing yields very poor fits to these data.

We repeated the analysis using linear regression instead of matrix inversion for the LV inference. The results were by and large similar and slightly inferior; they are shown in Supplementary Figure S7; Supplementary Table S4.2.

One should note that Mühlbauer et al. (2020) used a steepest-descent method, while our method did not. Therefore, our results can be further improved by adding a refinement cycle of steepest-descent optimization. We present an example in Figure 4C where the fit of the steepest descent optimization over the algebraic LV solution is depicted with a red line. The optimization reduces the error from 2,191 to 874.

#### 3.4.2 Published MAR inferences

In this section, we use two datasets presented in the MAR inference package MARSS. The first dataset, “gray whales,” consists of 24 annual abundance estimates of eastern North Pacific gray whales during recovery from intensive commercial whaling prior to 1900 (Gerber et al., 1999). It is thus to be expected that the whales are initially relatively far from the carrying capacity of the system. The second case consists of data for wolf and moose populations on Isle Royale in Lake Superior between 1960 and 2011; this dataset was used by Holmes and colleagues (Holmes et al., 2020) to demonstrate usage of the MARSS R package.

**FIGURE 5:**
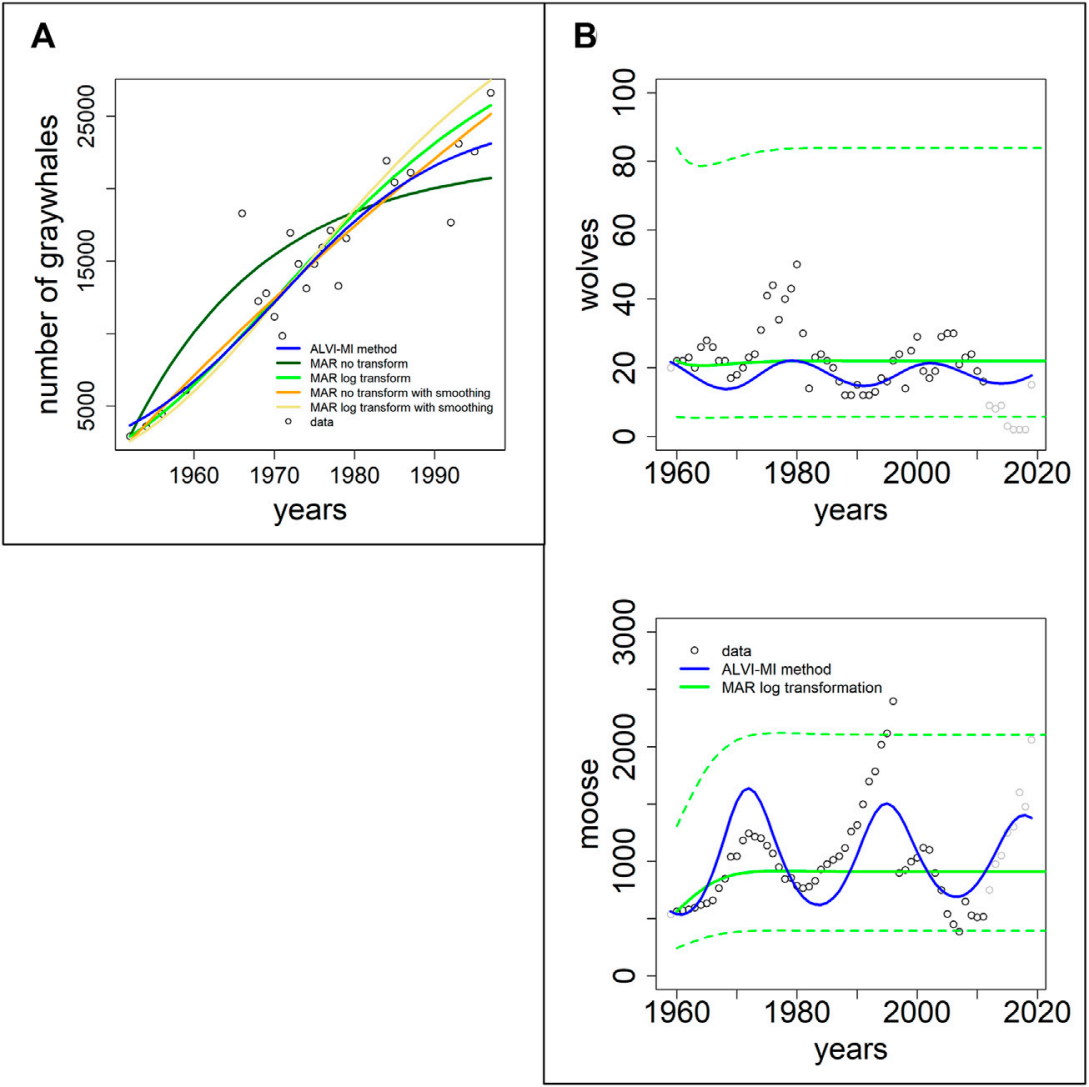
Two datasets of wildlife observations. Column **(A)** Abundance data of gray whales (Gerber et al., 1999). The plot shows results from ALVI-MI in blue; various MAR estimates are displayed with dark and light green, orange and yellow lines. Column **(B)** Wolves and moose on Isle Royale (Vucetich, 2021). The original data used for parameter estimation are displayed with black circles, data not used by the estimation processes are shown in gray, ALVI-MI results are in blue, MAR estimates using log-abundances are displayed with green lines. The dashed lines indicate confidence intervals for the MAR estimates. Values of the estimates can be seen in Supplementary Table S6.

Figure 5A shows fits to the gray whale data (Gerber et al., 1999). LV noticeably outperforms MAR, even though the data came from a MAR demonstration. In particular, the MAR results (without transformation) suggest that the whales are close to regaining their carrying capacity, which seems to contradict the trend in the data. The SSEs can be seen in Table 1. It is unclear why the MAR method without transformation does not perform better. As it stands, the estimates are inadequate (with the highest SSE) and have a very high variance for the error. An LV model with one variable is a logistic function, and the LV fit represents initial quasi-exponential growth that starts to slow down after a while. This behavior nicely reflects the fact that the whales were recovering from very small numbers due to overfishing but the population is apparently still much below the carrying capacity.

Figure 5B returns to the Isle Royale dataset from (Vucetich, 2021), which we already used in the context of examples from the collection of Mühlbauer and colleagues (Mühlbauer et al., 2020); *cf.* Figure 4. Holmes et al. (2020) used only the data of wolves and moose for a MAR analysis but extended them over a longer time horizon. Specifically, eight datapoints were added since the former usage of this dataset by Holmes and colleagues, from 2012 to 2020 (gray symbols in Figure 5B).

The results of the MAR model are identical with those published in, with the same log transformation and z-scoring of the data, and the same parameter values were inferred. The result consists of acceptable estimates, although we found a slightly better fit without the z-scoring. Still, for a direct comparison, we opted to present the example exactly as Holmes et al. (2020) did. Interestingly, these fits miss all oscillatory behavior seen in the data. The LV results do show oscillations but clearly suffer from the disruption in the wolf population in 1981 and 1982, as discussed before.

Because we used in this example only MAR with log transformation, we display the confidence intervals for the MAR model as dashed green lines. Very few datapoints are outside the confidence intervals.

#### 3.4.3 Performance of MAR and LV models with different initial conditions

We decided to test the hypothesis that MAR might perform better if the simulations were initiated near the steady state, because the model then would not be affected much by the non-linearities in dynamics, which can strongly affect a simulation starting far from the steady state. For this purpose, we again used the artificial LV and MAR artificial systems utilized in Figures 1, 2.

We started each simulation with six different initial conditions calculated as the system’s non-trivial steady-state values multiplied by 0.001, 0.01, 0.1, 1.9, 10, and 100. For each combination of artificial system type and initial conditions we again used two different sampling methods: noisy datasets were produced by sampling 40 random points from a 100-point time series, while replicate datasets were created by running five simulations for each case with sampling 15 pre-determined points. We used ALVI-MI with 8 degrees of freedom and MAR with the same configuration in almost all cases, for the sake of a fair comparison across the different initial conditions. Degrees of freedom used in the different initial conditions experiments can be seen in Supplementary Table S11.

We collected three metrics to assess the different cases. The first was the sum of squared errors (SSEs) of the fits against a noise-free time courses of the artificial systems. The objective was to evaluate which method could recover the noise-free dynamics more accurately. The results can be seen in the Supplementary Figures S12, S13; Supplementary Table S9.1 for the artificial LV system and in Supplementary Figures S14, S15; Supplementary Table S10.1 for the artificial MAR system. Both cases reveal an increase in SSEs as the simulations start further away from the steady state. For the artificial LV system, ALVI-MI performed better than MAR when the initial conditions were set further away from the steady state, which was not the case in the MAR synthetic datasets. This observation supports the claim that MAR will have difficulty obtaining a good fit to the data if extreme non-linear dynamics are present.

The second metric was the SSE for the last five points of a fit against the last five points of the noise-free time course of the systems, which allowed us to test if the methods could capture the original system steady state accurately. The results can be seen in the Supplementary Table S9.2 for the artificial LV system and Supplementary Table S10.2 for the artificial MAR system. As before, we can note an increase in SSEs as the simulations start further away from the steady state, but less pronounced than in the last metric. No method distinguishes itself for being better or worse in this metric. Finally, we tested to what degree the different methods estimated the parameters for the artificial systems correctly. Because we cannot compare MAR estimates inferred from an LV artificial system or LV estimates inferred from a MAR system, we counted the number of estimates with signs opposite of the original values. A high number of these signal flips indicates that the estimates are not capturing the true sense of the interactions. The results can be seen in the Supplementary Table S9.3 for the artificial LV system and Supplementary Table S10.3 for the artificial MAR system. These results show that the LV models capture the interactions of the artificial LV system better as the MAR models better capture the artificial MAR system. This result was expected, although it did not reveal any tendency directly associated with the initial conditions.

In conclusion, we found that MAR will have difficulty obtaining a good fit to the data if non-linear dynamics are present. The data also suggest that both methods have greater SSEs as the starting point shifts away from the system’s steady state.

## 4 Discussion

We have compared methods for inferring interaction parameters in LV and MAR models of population communities that are composed of several coexisting species. These modeling approaches are derived from slightly different philosophies and comparing them fairly and comprehensively is not straightforward, as one consists of ODEs and the other of discrete recursive equations. We therefore focused specifically on the goal of comparing the two approaches from the point of view of someone who is interested in quantifying the dynamics of a mixed community. As parameter estimation strategies, we used the published MARSS method for MAR models (Holmes et al., 2012) and a recently introduced algebraic LV inference method [ALVI; (Voit et al., 2021)].

Our comparisons required some choices. First, MAR could theoretically use information from several earlier timepoints (*t, t*-1*,* t-2*, t*-3, … ) to predict the value of a variable *X* at time *t*+1. We decided to use only information from time *t* to predict *X*_*t+1*_, which in the literature is called MAR(1), for three reasons: First, most studies in the ecological literature have used MAR(1). Second, accounting for information from earlier timepoints would lead to an explosion in the number of parameters to be estimated, and the time series are already too short for good inferences, according to Certain et al. (2018). Third, ODEs do not have memory, so that a comparison would seem unfair. If MAR models with memory were considered, one should probably compare them to delay differential equations (DDEs). In other words, the decision for MAR(1) appears to provide the fairest comparison between MAR and LV.

Another choice we had to make was the type of noise for creating our synthetic data. Most studies focusing on parameter estimation methods consider observational noise: Perfect time courses are generated, and noise is secondarily superimposed, usually with a variance proportional to the value of the investigated variable. Here we decided to use process noise, which corresponds to uncertainties incurred by the system as it progresses from one state to the next. We note again that the terminology of “noise” is somewhat misleading as it is often the result of environmental variations, which are typical and important in ecological systems. The significant difference between the two types of noise is that process noise accumulates and often tends to result in time series exhibiting erratic oscillations (Supplementary Figure S6). This type of variation is natural for MAR systems but not straightforwardly accommodated by the LV format. Non-etheless, because we considered this type of noise as potentially more appropriate than static observational noise, we mimicked it by simulating the system with a discretized version of the LV structure. This decision pertained only to test data we created to compare the different models. Actual data, as we analyzed in Section 3, most likely contain a mixture of process and observational noise, which can hardly be teased apart based on the data alone.

A smoothed representation of a dataset implicitly integrates information that is not explicit in the data. This integration step is not entirely unbiased and requires prudent judgment, because it must answer the following questions, often without true knowledge of the system: Are the deviations between the data and the smoothing function due to (random) noise or are they part of a true signal? For instance, do they belong to a trajectory exhibiting true oscillations? Also, if a few data points deviate much more than all others from the smoothing function, are they true peaks or valleys or are they statistical outliers? It is difficult to answer these questions objectively, but two features of the data are of great benefit: First, if the variation in noise amplitude is much smaller than the range of signal values (high signal-to-noise-ratio), the distinction between signal and noise is relatively straightforward. Second, if the data come in replicates, they may support or refute the potential of true oscillations or peaks at certain time points in the data. Even if only one dataset is available, the biologist familiar with the phenomenon at hand usually has developed an expectation regarding signal and noise, and if there is no biological rationale for expecting oscillations or strong deviations from some simple trend, the smoothing strategies are flexible enough to allow the integration of the biologist’s knowledge and expectations. The result of the smoothing process therefore is a synthesis of all relevant information, constrained by external knowledge and reasonable expectations. Of course, it is also feasible to create alternative models with different thresholds between signal and noise and to analyze them side by side.

Algebraic LV inference (ALVI) allows a choice between two variants (linear regression or matrix inversion). The former is simpler, because it uses all points available, and faster since no data samples need to be chosen. In most cases tested, it also produces good fits and estimates. However, the matrix inversion variant usually produces slightly better results and works well even in occasional cases where the regression solution fails (Table 1). It also offers a natural approach to inferring comprehensive ensembles of well-fitting model parameterizations. While both variants are quite effective, it is of course possible that other parameter estimation methods could outperform both in some or even all cases of LV inferences. If so, our overall conclusions still stand; in fact, the differences between LV and MAR would be even more pronounced.

Our overarching goal of inferring interaction parameters may in itself create a slight disadvantage for MAR models, because these models were designed for phenomena with random noise and, in particular, for characterizing the structure of this noise. As a possible consequence, MAR may have allocated some of the true dynamics into the noise estimates and that may have impeded the MAR parameter estimation. Also, Certain et al. (2018) prescribed the length of the data series as at least five times greater than the number of non-zero interaction elements in order to recover interaction signs correctly. This condition was clearly not satisfied in the examples we analyzed, and the same shortcoming applies to most actual observations in real-world contexts, especially in biology [e.g., (Dam et al., 2020; Dam et al., 2016)]. In the end, we considered data such as those complied by Mühlbauer et al. (2020) as a typical and representative target for inference analyses with the goal of extracting information regarding interactions among populations.

The MARSS software makes modeling with MAR models easy, although not entirely automatic, as many options must be tested to find the one that returns the best fit in a specific situation. For example, one must decide which variables should have the same noise level and whether to use the initial datapoints as initial conditions or estimate them. In contrast to MARSS, which has been vetted over close to a decade, the ALVI methods for LV models are recent (Voit et al., 2021), and while all steps are straightforward and code is available on GitHub (Olivença et al., 2022), no other public software exists that encompasses all methodological steps in a streamlined manner. This novelty of the method suggests that ALVI has the potential of being refined and made more efficient and user friendly. For example, MARSS uses a steepest-descent optimization step, which ALVI presently does not. Although ALVI already performs better than MARSS (Table 1), it might be possible to improve its results further by adding a refinement step based on steepest-descent optimization, as we illustrated with the example in Figure 3C. Generally, steepest-descent methods tend to get trapped in local minima if the initial guesses are poor, but using ALVI in the first step, one would likely not encounter this problem, as the solutions are already very good and could be used directly as initial guesses for the refinement step. A second example of potential future improvement and automation is the adequate smoothing of the raw data with splines, which requires the determination of a suitable number of degrees of freedom and may also suggest beneficial weights for different variables within a dataset.

It might be interesting in the future to study how well MARSS deals with non-normal process noise. The algorithm used by MARSS assumes the noise nature to be normal, which is a fair assumption in many cases. However, if that assumption is severely violated, it should be interesting to test what happens to the estimation of not only the noise itself, but also of the inferred parameters.

A comparison of MARSS results with or without log transformation of the dependent variable abundances did not yield clear results. If the MAR models are to be viewed as multispecies competition systems with Gompertz density dependence, as suggested in (Certain et al., 2018), a log transformation is required (Supplementary Section S1.3). We did obtain good SSEs for the artificial MAR datasets, which were essentially of this type. In addition, the log transformation helps MAR deal with non-linearities. While inferences for LV models usually benefit from smoothing, the same is apparently not true for MAR models, where smoothing leads to improved data fits in some cases, but certainly not always (Table 1).

Because the LV structure is continuous, solutions can directly be evaluated at any point or for any interval between the points in the numerical solution. MAR does not truly reveal a time resolution higher than its intrinsic interval between solution points but addressing this issue, Holmes et al. (2012) demonstrated with the MARSS R function that it is feasible to interpolate any number of missing values between the known datapoints, and that this method can be used to decrease the time unit for stepping forward. While this step does not make the MAR model as densely time-resolved as an ODE model, it mitigates the apparent granularity disadvantage considerably. It also increases the computational requirements of MARSS considerably.

Concerning the analysis of the effect of initial conditions, presented in Section 3.4.3, the result may have been influenced by the particular model structure or the sampling. Changing the initial conditions created quick but intense dynamics near the initial part of the simulation. The sampling scheme may not have been able to capture these dynamics accurately, causing the observed result that all methods yielded higher SSEs when the simulation started further away from the system’s steady state.

Estimation and inference methods typically do not scale well. The algebraic LV inference bucks this trend, at least to some degree, as both the smoothing and estimation of slopes are performed one equation at a time. Thus, instead of scaling quadratically, the inference problem scales linearly. The computing time for matrix inversion or linear regression is essentially the same for all realistically sized models. Thus, the only time-consuming step within ALVI-MI is the choice of datapoints. An exhaustive test for all combinations grows quickly in the number of analyses, but it is always possible to opt for a much faster random search, for instance, through Monte-Carlo simulation. In fact, this method is so fast that many inferences can be obtained in a short period of time and the best solutions are retained while other solutions are discarded. The result might not necessarily be the best possible solution, but it can still provide an excellent fit or, at the very least, a valuable starting point for a steepest-descent refinement optimization. Importantly, a collection of good solutions can be collated to establish an ensemble of well-fitting models, which will often yield more biologically meaningful insight than a single optimized solution.

Our overarching conclusion, with numerical results summarized in Table 1, is that LV outperforms MAR in the vast majority of analyzed cases, by yielding often substantially lower SSE values. However, one must note that while the two approaches have similar goals, they are best suited in different situations. MAR models are very useful for investigations where the quantification of noise is of importance because noise is characterized in MAR by a parameter that can be estimated together with the other parameters. Along the same lines, we noticed that MAR performed rather poorly for artificial LV datasets where the model had a fair number of zero-valued parameters. Thus, although we do not completely understand the reasons, our study suggests that MAR should not be used in cases where many parameters may have zero values. By contrast, MAR models proved to be very effective in dealing with process noise when there were no replicates. This outcome was true for both the artificial LV and MAR data. Finally, MAR appears appropriate for data that display non-linearities that align with the MAR model structure, possibly upon a log transformation of the data.

LV models are better suited to capture the dynamics in many datasets because this architecture is able to deal with complex non-linearities. In fact, the LV structure was shown to be capable of modeling very complex non-linear dynamics (Peschel and Mende, 1986; Voit and Savageau, 1986; Vano et al., 2006) and has no problems with zero-values parameters as we encountered them with MAR. Our experiments with the artificial LV and MAR data suggest that LV models should be used when replicates for the different time points are available or when the influence of process noise is moderate.

## Data Availability

The datasets presented in this study can be found in online repositories. The names of the repository/repositories and accession number(s) can be found in the article/Supplementary Material.
