# Supplementary material for: Inference of dynamic interaction networks: A comparison between Lotka-Volterra and multivariate autoregressive models
Source: Front Bioinform. 2022 Dec 22;2:1021838. doi: 10.3389/fbinf.2022.1021838 (PMC9815445; doi:10.3389/fbinf.2022.1021838)
Supplement: Supplementary file 1 [file DataSheet1.PDF]

Supplementary Material

Inference of Dynamic Interaction Networks:

A Comparison Between Lotka-Volterra and Multivariate Autoregressive Models

Daniel V. Olivença, Jacob D. Davis, Eberhard O. Voit

1 Models and Methods

1.1 Lotka-Volterra models

For a single variable, Lotka-Volterra (LV) models (Eq. (1)) in the main text) reduce to the well-known logistic growth law

$$\frac{dX_i}{dt} = a_i X_i - b_{ii} X_i^2, \qquad [\text{ S1 }]$$

where the ratio a_i/b_{ii} is called the "carrying capacity" of the system, which corresponds to the non-trivial steady state (Vogels et al., 1975). If time-dependent environmental inputs are to be considered, one may add one or more terms $\gamma_{ik} X_i U_k$, where U_k is the kth element of a vector of these inputs and the coefficients γ_{ik} are weights that quantify the effects of the factors on species X_i (Dam et al., 2020, 2016; Stein et al., 2013). The left-hand side is often written as $\dot{X}_i$.

The LV system is a *canonical* model in the sense that its mathematical structure is immutable and scalable to any dimension (Voit, 2000). Such a canonical model may serve as a template to construct models of different systems that reasonably satisfy the following assumptions (Fort, 2020):

- encounters between and within species are representable by mass action kinetics;
- the environment does not change during the process, unless environmental variables are explicitly formulated as described above;
- the parameter values do not change during a simulation experiment;
- the species respond to one another instantaneously;
- for very small population sizes, interactions are negligible and the change (growth) of each population over time is initially proportional to its size, resulting in initial exponential growth;
- adaptations of species are absent or negligible.

Although the model structure and these assumptions might appear to be unduly rigid, LV models are extremely rich in the repertoire of their possible responses. In fact, the LV structure was shown to be

capable of modeling any type of differentiable nonlinearities, including different kinds of oscillations and chaos (Vano et al., 2006), if sufficiently many auxiliary variables are permitted, which have mathematical, but often no real biological meaning (Peschel and Mende, 1986; Savageau and Voit, 1987; Voit and Savageau, 1986). At the same time, the LV structure without auxiliary variables has intrinsic limitations. For example, it is not well suited for metabolic pathway systems, because a simple conversion of a substrate X_1 into a product X_2 would require X_2 to appear in its own synthesis term, although the generation of X_2 depends in truth only on X_1 and possibly some modulators (see (Voit, 2013) for this and other limitations).

LV models were initially used to describe the dynamics of predator and prey populations or of populations that compete for the same resources, but the same equations have also been used in entirely different contexts and fields, including physics (Hacinliyan et al., 2010; Nambu, 1986), pollution assessment (Haas, 1981), economy (Gandolfo, 2008; Zhou and Chen, 2006), manufacturing (Chiang, 2012), and sales (Hung et al., 2017).

Beyond the fact that LV models can be formulated very easily, another significant advantage over other systems of nonlinear ODEs is the fact that the parameter values of LV models can be estimated with linear regression methods if time series data are available (Voit and Chou, 2010). As an intriguing alternative, the linearity also permits us to select the values of variables and slopes at n+1 time points and to obtain parameter inferences by solving a set of linear algebraic equations (see below). It is furthermore possible to estimate parameter values from sufficiently many steady-state abundance profiles of species that initially coexist under comparable conditions but ultimately survive in different proportions (Voit et al., 2021; Xiao et al., 2017).

1.2 Estimation of LV Parameters Based on Slopes of Time Courses

This section explains in some detail an approach to parameter estimation that uses the Algebraic Lotka-Volterra Inference (ALVI) method. For a detailed explanation of the ALVI method itself, see (Voit et al., 2021).

1.2.1 Smoothing

Even though one might consider the smoothing task of raw data as a conceptually separate issue from the actual parameter inference, the two are so closely intertwined in our analysis that it appears useful to discuss a few options. The goal of smoothing is two-fold. First, it is beneficial to reduce or even remove noise from the raw data, and second, this smoothing greatly aids the determination of slopes of the experimentally observed time courses (see later).

We explored a number of methods for smoothing time course data and keeping noise in check (Batista Júnior and Pires, 2014; Eilers, 2003; Vilela et al., 2007), cognizant of the fact that empirical raw data alone do not provide enough information of what is noise and what is relevant signal in the dynamics of the phenomenon under study. In this analysis, smoothing splines and local regression methods like LOESS (locally estimated scatterplot smoothing) and LOWESS (locally weighted scatterplot smoothing) turned out to be particularly useful. A detailed description of these methods can be found in (Cleveland, 1981).

In a nutshell, (regular) splines are piecewise polynomial functions that: (1) pass through all sample points; (2) are continuous; and (3) have first and second derivatives that are continuous at junction points between adjacent intervals. In a smoothing spline, the first condition is substituted by a least-

squares fit that is balanced with an additional criterion that penalizes splines with high second derivative values, which indicate local roughness (Cleveland, 1979; Garcia, 2010; Loader, 2012).

LOWESS and LOESS algorithms use locally weighted polynomial regression. LOWESS is used for univariate smoothing and consists of computing a series of local linear regressions, with each local regression restricted to a window of x-values. Smoothness is achieved by using overlapping windows and by gradually down-weighing points in each regression according to their distance from the anchor point of the window. LOESS was developed for fitting a smooth surface to multivariate data. It is a generalization of LOWESS in that locally weighted univariate regressions are replaced by locally weighted multiple regressions. While LOESS is more versatile, LOWESS is faster and sometimes succeeds when LOESS fails (Cleveland, 1979; Cleveland and Devlin, 1988; Smyth, 2020). Locally-weighted polynomial regression methods have 'span' and splines have 'degrees of freedom,' which are parameters that control the degree of smoothing.

If we select many points from the smoothing spline, we overcome the problem of data scarcity that is inherent in many datasets. In fact, sampling from the smoothing spline allows the subsequent parameter inference method to access a larger amount of information and thereby to mitigate noise amplification.

As mentioned in the Main Text, smoothing requires caution as it may obscure the true signal. A simple example is shown in Figure S11.

1.2.2 Slope Estimation

Independent of the options and intricacies of obtaining smoothed time courses of all variables, it is well known that the estimation of slopes from data is more strongly affected by noise than the data themselves (Knowles and Renka, 2014). Expressed differently, if the noise is left unchecked, its effect on the estimated values of the slopes tends be higher than its effect on the values of the variables. This observation mandates means of obtaining good slope estimates.

One of the simplest approaches is the *three-point method* for data at equally spaced time points, where the slope of a trajectory at time point t_k is taken as the average of the slopes at time points t_{k-1} and t_{k+1} (Burden et al., 1993; Voit and Almeida, 2003). More sophisticated methods were reviewed in (Batista Júnior and Pires, 2014; Cleveland and Grosse, 1991; Eilers and Marx, 1996; Vilela et al., 2007). For long, dense time series, moving average and collocation methods with or without roughness penalty (Ramsay et al., 2007) are often very effective. However, they tend to be unsuited for biological time series data because biological measurements are usually quite sparse and obtained over a relatively short time horizon.

An alternative that is usually superior is smoothing, as described in the previous section. The main result of smoothing with splines is a reduction or even removal of what is believed to be noise in the data. An important consequence is that the slope at each point can be computed directly from the smoothing spline, which after all is an explicit function. This step of slope determination offers two options: it allows us to estimate slopes exclusively for the measured data points or to sample the smoothing function for any number of other points, which yields a larger set of numerical values for variables and slopes (Voit and Almeida, 2004).

1.2.3 Conversion of ODEs into systems of algebraic equations

If data are available as time series, it is mathematically feasible and beneficial to estimate slopes (for instance, from smoothing splines) and to convert the inference problem from one based on ODEs into

one exclusively using algebraic functions (Varah, 1982; Voit and Almeida, 2004; Voit and Savageau, 1982a, 1982b), as it is reviewed below.

Suppose the growth and interaction parameters of an LV system are to be estimated from time series data of the dependent variables X_i. The smoothing of these data facilitates the estimation of slopes $S_m(X_i)$ of the trajectories of all variables at a set of time points t_m, $m = 1, \ldots, M$. These time points may or may not correspond to the measured data. In fact, the smoothing permits the computation of slopes at arbitrarily many time points within the observation interval. However the slopes are computed, they correspond to the derivatives of the spline of X_i at the given time points. Substituting numerical values of all variables and slopes from the smoothing splines into Eq. (1) of the Main Text yields a system of $n \times M$ linear algebraic equations containing all system parameters:

$$S_i(t_m) = a_i X_i(t_m) + \sum_{j=1}^{n} b_{ij} X_i(t_m) X_j(t_m), \quad i = 1, \ldots, n; \quad m = 1, \ldots, M \qquad [\text{ S2 }]$$

If environmental inputs $\gamma_{ik} X_i U_k$ are to be considered as well, they are added to the equations and substituted with numerical values, if known; if not, their rates γ_{ik} are estimated together with the parameters a_i and b_{ij}.

A caveat of this conversion of ODEs into a system of algebraic equations is a possible time warp (see end of Chapter 5 of (Voit, 2017)). The reason is that time is explicitly eliminated from the procedure. Nonetheless, this type of slope-based estimation usually provides good results, or at least good initial guesses for other optimization approaches, such as traditional gradient methods, as we demonstrated with the case in Figure 4c.

Suppose the dependent variables are not zero within the dataset obtained from smoothing. If so, we can divide both sides of the M equations for X_i in Eq. [S2] by the value of the dependent variable at the appropriate time point. This step is not mandatory but explicitly linearizes the equations. The case of variables with values of zero is typically not very interesting or can be handled by eliminating the variable or parts of the time series.

1.2.4 Parameter inference

Once all differentials are replaced with estimated slopes, the inference of parameter values for LV-models offers two options: because the system of algebraic equations is linear in the parameters, we may optimize all parameter values through simple multivariate linear regression (ALVI-LR), where we may use either all data points or iterate the regression with subsets of points, which naturally leads to an ensemble of well-fitting models.

An interesting alternative is to use just n+1 of the data points and slopes of each variable, if n is the number of variables, which results in a system of linear equations that can be solved with simple algebraic matrix inversion (ALVI-MI). Again, choosing different data points leads to many solutions. The best of these are retained and naturally lead to an ensemble of solutions. These can be further analyzed, for instance, with respect to unrealistic over- or undershoots, model robustness and identifiability. They can also be used to determine to what degree the LV format is adequate for the available data (Voit et al., 2021).

1.2.5 Example of Algebraic LV Inference (ALVI)

To illustrate the parameter estimation procedure with ALVI, we use the noisy LV dataset presented in in Table S1.2. First, we smooth the data with a spline or LOESS. For this illustration, we explored different options and, in the end, chose 5, 8, 11 and 5DF-splines for X_1, X_2, X_3 and X_4, respectively. We then computed the first derivatives of the splines to estimate the slopes at various time points. At this juncture, we may ignore the raw data and only use the spline values, or we could instead use the original data, especially if we think they characterize the studied phenomenon well. In our experience, using the spline usually produces better results in the case of noisy LV data.

As an example, consider the first differential equation and the first datapoint, at $t = 1$:

$$\frac{dX_1}{dt}(1) = a_1X_1(1) + b_{11}X_1(1)X_1(1) + b_{12}X_1(1)X_2(1) + b_{13}X_1(1)X_3(1) + b_{14}X_1(1)X_4(1)$$

We substitute numerical values for the slope and for all variables on the system equations,

Time	Slope_X_1	X_1	X_2	X_3	X_4
1	0.039	1.263	0.363	1.778	0.001

which yields

$$0.039 = a_1 \times (1.263) + b_{11} \times (1.263)^2 + b_{12} \times (1.263\ ) \times (0.363)$$
$$+b_{13} \times (1.263) \times (1.778) + b_{14} \times (1.263\ ) \times (0.001).$$

The same steps are performed for every equation and every chosen time point. The result is a system of linear equations with as many equations as chosen time points; each equation has n+1 = 5 unknown parameters, where n = 4 corresponds to the number of dependent variables.

Now we have two options: We may use linear regression (ALVI-LR) or matrix inversion (ALVI-MI). ALVI-LR uses every equation and every chosen time point and performs linear regression to produce estimates for the parameters. For the alternative of ALVI-MI, we choose some sample of data points that, when combined with the equations, generates a number of equations equal to the number of parameters to be estimated. If these equations are linearly independent, the system is solvable and the solution is unique, allowing us to obtain estimates for the parameters by simple matrix inversion. Examples of results can be found throughout the Main Text.

1.3 Multivariate AutoRegressive (MAR) models

Multivariate autoregressive (MAR) models are discrete recursive models. Their format is shown in Eq. (3) and (4) of the Main Text. (Holmes et al., 2020)

(Certain et al., 2018; Ives, 1995)The initial MAR models may be augmented with state variables that simulate the observation process, and these models are called Multivariate Autoregressive(1) State-Space Models (Certain et al., 2018; Holmes et al., 2012); we will not analyze these as it would distract

from our main focus. Moreover, for the comparisons in this study, we are not considering the influence of environmental variables, so the corresponding terms will be omitted henceforth.

It is considered an advantage in ecology if models explicitly take the influence of environmental factors into account (Certain et al., 2018; Hytti et al., 2006), which is the case for MAR. The availability of estimation software like MARSS (Holmes et al., 2020, 2012) has greatly increased the appeal of MAR models see Section 1.5).

1.4 Structural similarities between modeling formats

The two modeling formats appear to be rather different mathematically, with one consisting of systems of ODEs and the other one of discrete-recursive equations. Nonetheless, they can be compared in terms of practical considerations (see Main Text) and also with respect to their mathematical representations (below). These comparisons demonstrate that the two models can behave quite similarly if the community of populations operates relatively close to a stable steady state. By contrast, if their abundances vary widely, the two models often show strongly diverging results, as the linearity of the MAR model can deviate considerably from the nonlinearities of the LV model.

Purely considered on mathematical grounds, MAR is defined recursively in Eqs. (3) and (4) of the Main Text. By omitting environmental variables, we directly obtain

$$X_{i,t+1} = \alpha_i + \sum_{j=1}^{n} \beta_{ij} X_{j,t} + w_{i,t}; \quad i = 1,2,\dots,n; \quad w_{i,n} \sim N(0, \delta_i). \qquad [\text{ S3 }]$$

Suppose the dynamics of the MAR model operates near the steady state of the differential equations, so that $X_{i,t+1} - X_{i,t} \approx 0$ for all i. If so, we obtain

$$X_{i,t+1} - X_{i,t} \approx 0$$

$$\Leftrightarrow \alpha_i + \sum_{j=1}^{n} \beta_{ij} X_{j,t} + w_{i,t} - X_{i,t} \approx 0; \quad i = 1,2,\dots,n; \quad w_{i,n} \sim N(0, \delta_i). \qquad [\text{ S4 }]$$

The similarity between the LV model and [S3] and [S4] can be seen if we evoke Euler's discretization method for determining the numerical solution for the LV model. Preceding other solution methods by two centuries, Euler's method can be seen as a linear precursor of modern methods that include higher derivatives, such as the Runge-Kutta method. Its simplicity facilitates the comparisons between recursive models and ODEs.

Formulating the typical Euler step for the LV model transforms the ODE into a series of discrete steps of the type

$$X_{i,t+h} = X_{i,t} + h * \left.\frac{dX_i}{dt}\right|_{X_i = X_{i,t}} = X_{i,t} + h * X_{i,t}\left(a_i + \sum_{j=1}^{n} b_{ij} X_{j,t}\right), \quad i = 1,2,\dots,n, \qquad [\text{ S5 }]$$

Where h is the step size of Euler's approximation and the derivative dX_i/dt, which corresponds to the left-hand side of the differential equations in Eq. (1) of the Main Text, is evaluated at time t.

Simple transformation of [S5], close to the steady state, yields for $i = 1,2,\dots,n$:

$$X_{i,t+h} - X_{i,t} \approx 0$$

$$\Leftrightarrow X_{i,t} + h * X_{i,t}\left(a_i + \sum_{j=1}^{n} b_{ij}\, X_{j,t}\right) - X_{i,t} \approx 0$$

$$\Leftrightarrow a_i + \sum_{j=1}^{n} b_{ij}\, X_{j,t} \approx 0$$

$$\Leftrightarrow a_i + \textstyle\sum_{j=1}^{n} \tilde{b}_{ij}\, X_{j,t} - X_{i,t} \approx 0 \qquad [\text{ S6 }]$$

Thus, if we disregard the Gaussian noise, w_{ij}, in the MAR model, the two sets of near-steady-state equations, [S4] and [S6], are equivalent if $\tilde{b}_{ij}$ equals b_{ij} for all $i \neq j$ and $b_{ij} + 1$ for $i = j$. They are both linear, although the dynamic LV model itself is non-linear. Expressed differently, the MAR and LV models have the same steady state and their dynamics close to the steady state is typically similar. Expressed differently, as long as the nonlinearity of the LV system is close to linear or if its dynamics does not deviate much from the steady state, the LV and MAR models may be expected to yield similar results.

1.5 MARSS

MARSS is a software package for analyzing MAR models with or without log-transformation of the dependent variables (Holmes et al., 2012). Its use requires several steps.

1 – Specify key MARSS settings:

Parameters		Parameter format
**B** –interaction parameters	**B** = "unconstrained"	Matrix with potentially different elements
**U** –intrinsic growth parameters	**U** = "unequal"	Vector with potentially different elements
**Q -** correlations of deviations	**Q** = "diagonal and unequal"	Matrix where the main diagonal elements are real values and other elements are zero
**Z** –individual and interaction bias parameters of observations	**Z** = "identity"	Identity matrix
**A -** bias in observations	**A** = "zero"	All elements zero
**R -** correlation structure of observation errors	**R** = "zero"	All elements zero
x0 - Initial values of the time series	x0 =	Initial values of the time series

Z, A and **R** correspond to so-called "observation variables," which simulate details of the observation process of the system variables. For our comparisons, we assume the observation of the target variables is perfect (**Z** = "identity") with no bias (**A** = "zero"), and no sources of noise affecting the observation process (**R** = "zero"). For more details, see MARSS manual (Holmes et al., 2020, 2012).

2 – In the MARSS function, the data must be formatted with variables in rows and observations in columns. If the data points are not equally distributed in time, they must be augmented by “NA” to force the interval between any two consecutive data entries to be of the same length. This is necessary to ensure the correct time structure of the data for the estimator.

3 – With this regularization, MARSS finds estimates for **U**, **B** and **Q**, that correspond to α, β and δ in Eq. (4) of the Main Text.

If MARSS does not converge, it is advisable to increase the maximum number of iterations. This step usually solves the problem but is different from the suggestion offered by Holmes and colleagues, namely, that the model assumptions should be checked (see p. 57 in (Holmes et al., 2020)).

Our setup is exactly the same as that proposed by Holmes *et al.* (Holmes et al., 2020, 2012) for the Isle Royale dataset, which the authors used to exemplify the inference of species interaction parameters with and without covariates. Some of the illustration examples were modeled differently in the literature but for the purpose of comparisons with LV models, this model structure was used. For example, the dataset for ‘gray whales’ was modeled by Holmes and colleagues with $\boldsymbol{\beta}$, the species interaction matrix, set to zero, whereas **R**, the matrix that captures the noise from the observational process, was estimated from the data. Because we are interested in the interactions between species, we do not focus on observational noise, and Holmes’ original setup was replaced with the one discussed above.

2 Case study 1: Synthetic LV data

For a representative illustration of the parameter inference process, we use the four-variable LV system detailed in the Main Text.

The smoothing and slope estimation steps followed directly the procedures described in Section 1.2 of these Supplements. The first derivative of the smoothing function was used to determine estimates of the slopes.

To infer numerical values for the parameters of a given equation, we have the choice between linear regression (ALVI-LR) and matrix inversion (ALVI-MI). For ALVI-MI, we choose points from the sample and use the corresponding slope estimates to create a system of equations with the same number of equations and unknowns. As we have 4 variables and 20 parameters, we need 20 independent equations and thus observations at 5 time points. For each time point we obtain the value for each of the 4 dependent variables and use these to populate the equations.

As an illustration for the noisy dataset, we choose 10, 8, 11 and 15DF-splines and time points t = 3, 8, 10, 14 and 25 for the noisy dataset. For the replicate dataset, we use 8, 10, 10 and 9DF-splines, and the ALVI-MI solution was calculated with spline points at times 2, 3, 4, 6 and 7. The time point selection for the ALVI-MI solution can be automated using a random or exhaustive search among all possibilities.

The noisy dataset (Figures 1a, S1a, and S4a) is representative of a study where each time point sample corresponds to a single observation taken when it is possible or convenient. By contrast, the replicate dataset (Figures 1b, S1b, and S4b) simulates a series of experimental replicates where the observations

were conducted multiple times, but at fewer time points, which the researchers suspect would contain valuable information or which were dictated by experimental constraints.

As an illustration of how the smoothing techniques work, we use splines and LOESS with different degrees of smoothing. The results for variable X_1 are shown in Figure S3. Choosing the optimal degree of smoothing is not a trivial matter. Too much smoothing ignores important details in the variable dynamics, while too little smoothing unduly highlights the noise. In the programing language R, the function “loess.as” allows the calculation of the optimum value for the span, which controls the smoothing. The user still must decide the degree of the polynomials to be used and choose from two criteria for automatic smoothing parameter selection: a bias-corrected Akaike information criterion (AICC) or generalized cross-validation (GCV). This choice is not always leading to the optimal solution, but it may be used to challenge earlier assumptions and settings.

For comparison, we used the same LV system to produce datasets that only have observation noise. The results are presented in Figure S4, along with fits from the different methods. In this case MAR does not fare as well as LV.

In the example shown in Figure 1 we used noise with standard deviation of 0.005. This level of noise does not perturb the simulation very far from the original dynamics. We returned to the same example but used a process noise standard deviation of 0.03 which will deviate the trajectory of the simulation considerably, as can be seem in Figure S10 a.

This will diminish the ability of all tested methods to capture the noise free dynamic because we do not have good data to build upon. MAR should have an advantage here because it can estimate the noise distribution parameters as parameters of the model and, as can be seen in Table S1.7 and Figure S10, it outperformed ALVI in the noisy dataset. In the replicate ALVI still presented lower errors.

We also note that, during our experiments with this dataset, the replication dataset produced consistently better results than the noisy dataset (Figure S10 and Table S1.7). This suggests a method to capture the true dynamic in a population affected by process noise that strongly distorts the simulation time series. This works by collecting parallel time series, with the same timepoints, for the same population and averaging the collected timepoints. If the noise is random, the law of large numbers guarantees that the mean will converge to the true value. This method will be easily applied to bacteria population in a wet lab, but it may be difficult to record parallel time series for natural populations.

2.1 Application of ALVI to synthetic data

An alternative to using the algebraic parameter inference method with matrix inversion (ALVI-MI) is linear regression (ALVI-LR); fits for the *noisy dataset* are shown in Figure S5. As in ALVI-MI, the parameter values are close to the true values and the fit is acceptable. The dynamics of the two are very similar.

ALVI yields better results than MAR when the data only have observational noise (Figure S4 and Table S2.5). This result is due to MARSS have been created for ecological data which are always supposed to have process noise. Also, the expectation maximization algorithm used in MARSS has difficulties to assign zero values to the Q matrix, where process noise is taken into account (Holmes et al., 2020).

Results obtained with MARSS or with ALVI-LR are rather robust if the data are noisy, whereas solutions with ALVI-MI may be sensitive to small alterations in the data. As an example, consider the synthetic MAR data presented in Figure 2 of the Main Text. Consider now an alternative sample obtained by applying the same level of noise but created with a different seed. The alternative dataset is almost indistinguishable from the original dataset in Figure 2, but it is possible that using the same sample of data points causes ALVI to "explode" (Figure S9). The reason is that Lotka–Volterra models in some instances can be structurally unstable, *i.e.*, small modifications in the model settings might alter the predictions very substantially (Lindström, 2019). Of course, this outcome is easy to spot and suggests that if one calculates a new set of splines, it may be advisable to search for a new point sample as well. In fact, using a different point sample in the given case led to very good results (not shown). More generally, using different point samples often leads to similarly good ALVI-MI fits, but may produce quite different parameter values, which is a sign of almost-redundancy or sloppiness within the LV system (Gutenkunst et al., 2007; Srinath and Gunawan, 2010; Vilela et al., 2009). The conclusion is that, even if several inferred fits are similarly good, the associated parameter values for a noisy point sample may not be optimal for another noisy sample, which may not be surprising due to sloppiness. In fact, we showed in a different example with noise that the inferred parameter values yielded a better SSE than even the true values (Section 3.1 of the Main Text). This issue of parameter uncertainty may be considered a problem but can easily be turned into a positive feature: Different noisy datasets or subsamples of these datasets can be used to create natural ensembles of models that characterize the underlying data in a robust manner and may even yield additional insights into the variability of the model parameters.

3 Comparative summary of the performance of LV and MAR in the presented examples

We compared the sum of squared errors (SSE) in all experiments from different inference methods and summarize the results in Table 1 in the Main Text. Inspection of the results renders it evident that LV clearly performs better than MAR. In a few cases, the ALVI-LR solution gives a better SSE than ALVI-MI, but the difference between the two are not substantial. ALVI-LR appears to be superior when the data are noise-free.

We used a one-sided Wilcoxon rank test to see if the differences in performance are significant. The results and alternative hypotheses for these tests are presented in Table S7.

The data support the earlier results showing that the LV inference per matrix inversion produces smaller SSEs than the other methods considered. Also, in the last two tests, the data do not show evidence that data smoothing reduces the SSEs in MAR.

Comparing the results of ALVI-LR and ALVI-MI with respect to the absolute value of the difference between true and estimated parameters, we obtained mixed results (Table S8). Indeed, a one-sided Wilcoxon rank test with the alternative hypothesis that the absolute errors in parameter values associated with ALVI-LR were smaller than those associated with ALVI-MI did not yield a significant *p*-value 0.7695.

Comparing the noisy and replicate datasets in Tables 1 and S8, the replicate datasets mostly present smaller SSE values. However, using the one-sided Wilcoxon rank test for the values in Table 1 with the alternative hypothesis—that the SSE values for the replicate dataset are smaller than those for the noisy datasets—did not yield a significant *p*-value (0.1331). If we only consider the values in Table S8, the test produced a *p*-value of 0.25, suggesting not to reject the null hypothesis that replicate

datasets are equal or worse than time series data (noisy datasets) for parameter estimation in LV using ALVI.

4 Supplemental Figures

Figure S1

	Initial Conditions
X_1	1.2
X_2	0.3
X_3	2
X_4	0.001
	**True Parameter Values**
α_1	0.044
b_{11}	-0.08
b_{12}	0.02
b_{13}	0.08
b_{14}	0
σ_1	0.005
α_1	0.216
b_{11}	-0.04
b_{12}	-0.08
b_{13}	0.04
b_{14}	0
σ_2	0.005
α_2	0.116
b_{21}	-0.16
b_{22}	0.16
b_{23}	-0.08
b_{24}	0
σ_3	0.005
α_4	0.2
b_{41}	0
b_{42}	0
b_{43}	0
b_{44}	-0.1
σ_4	0.005

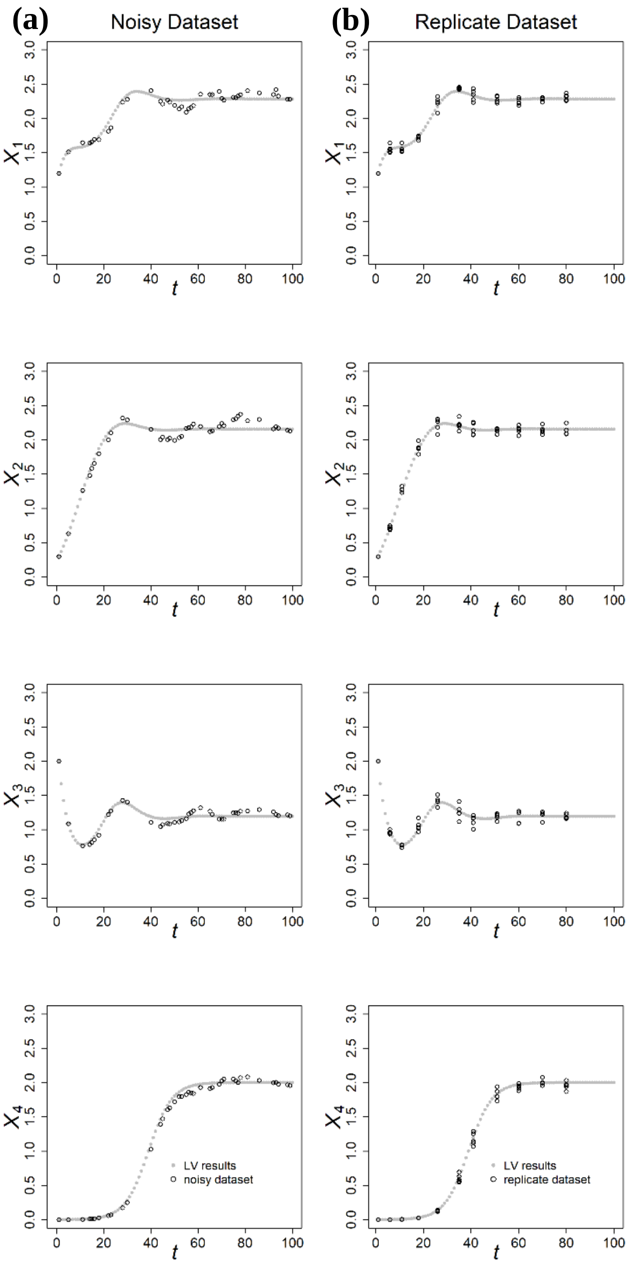

Figure S1: Synthetic time courses with process noise. The left panel contains initial conditions and parameter values for the synthetic LV example in Eq. 1 of the Main Text with four dependent variables. **Column a:** Noisy dataset – 40 points from the synthetic data were multiplied by random gamma noise with mode 1 and standard deviation equal to 0.01. **Column b:** Replicate dataset – 15 time points were chosen from five time series of synthetic data. The five time series were created by multiplying the value of the variable by a random gamma value with mode 1 and standard deviation of 0.005.

Figure S2

	Initial Conditions	ALVI-MI Estimates	ALVI-LR Estimates
X_1	1.2	1.2	1.2
X_2	0.3	0.3	0.3
X_3	2	2	2
X_4	0.001	0.001	0.001
	True Parameter values		
a_1	0.044	0.0440	0.0440
b_{11}	-0.08	-0.0800	-0.0800
b_{12}	0.02	0.0200	0.0200
b_{13}	0.08	0.0800	0.0800
b_{14}	0	0.0000	0.0000
a_1	0.216	0.2160	0.2160
b_{11}	-0.04	-0.0400	-0.0400
b_{12}	-0.08	-0.0800	-0.0800
b_{13}	0.04	0.0400	0.0400
b_{14}	0	0.0000	0.0000
a_2	0.116	0.1161	0.1161
b_{21}	-0.16	-0.1602	-0.1602
b_{22}	0.16	0.1601	0.1601
b_{23}	-0.08	-0.0799	-0.0799
b_{24}	0	0.0000	0.0000
a_4	0.2	0.2000	0.2000
b_{41}	0	-0.0003	-0.0003
b_{42}	0	0.0001	0.0001
b_{43}	0	0.0003	0.0003
b_{44}	-0.1	-0.0999	-0.0999

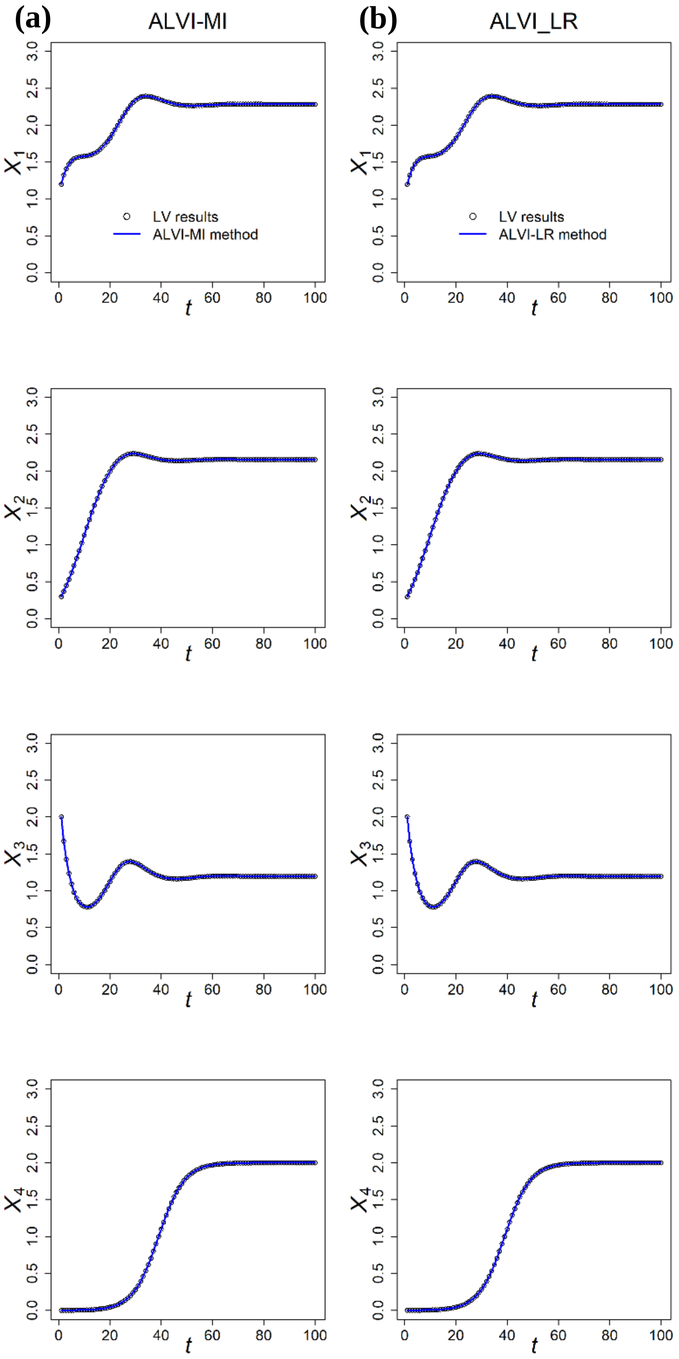

Figure S2: Estimates with alternative ALVI methods, using noise-free data. Column a: ALVI-MI and **Column b:** ALVI-LR with original synthetic LV data. The fits are of high quality (ALVI-MI SSE = 1.162229e-05 and ALVI-LR SSE = 3.289283e-07) and the parameter estimates are very close to the true parameters.

Figure S3

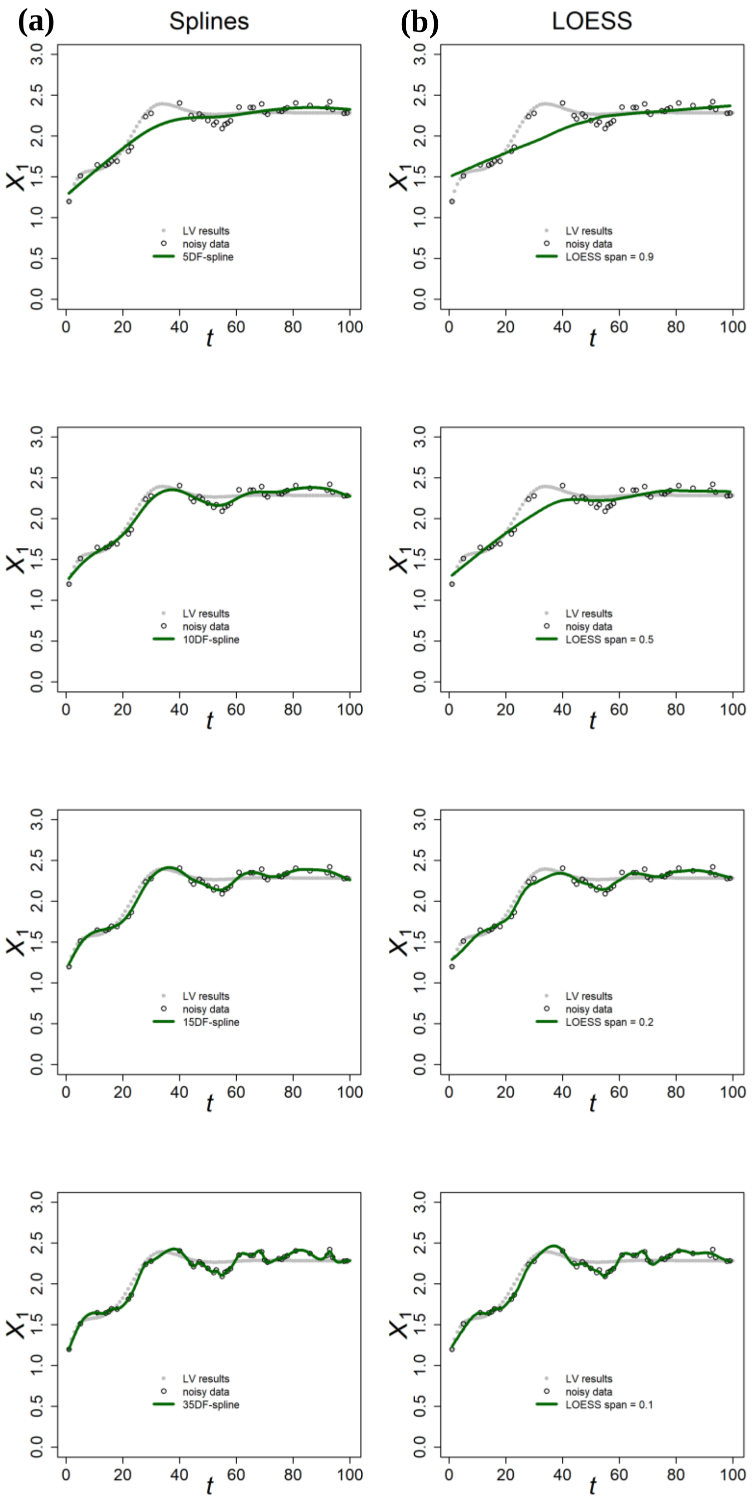

Figure S3: Smoothing of variable X_1 of Figure S1, subject to process noise. Column a: Splines with different degrees of freedom. **Column b:** LOESS with different span levels.

Figure S4

	Initial Cond.	Noisy Est.	Replicate Est.
X_1	1.2	1.26251	1.23614
X_2	0.3	0.36268	0.30334
X_3	2	1.77750	1.96120
X_4	0.001	0.00100	0.00100
	**Pars**	**Noisy Est.**	**Replicate Est.**
a_1	0.044	0.05719	0.04507
b_{11}	-0.08	-0.07001	-0.05512
b_{12}	0.02	-0.00061	0.00941
b_{13}	0.08	0.08336	0.05465
b_{14}	0	0.00338	-0.00213
a_2	0.216	0.25575	0.21364
b_{21}	-0.04	-0.10498	-0.02271
b_{22}	-0.08	-0.07085	-0.09048
b_{23}	0.04	0.09800	0.03119
b_{24}	0	0.01219	-0.00333
a_3	0.116	0.15452	0.15130
b_{31}	-0.16	-0.16217	-0.18840
b_{32}	0.16	0.16524	0.18628
b_{33}	-0.08	-0.12598	-0.10894
b_{34}	0	0.00315	0.00724
a_4	0.2	0.19984	0.19680
b_{41}	0	-0.00295	0.00823
b_{42}	0	-0.00444	-0.00506
b_{43}	0	0.00775	-0.00532
b_{44}	-0.1	-0.09704	-0.09971
Degrees of Freedom	10,8,11,15	8,10,10,9	
Point Samples	3,8,10,14,25	2,3,4,6,7	

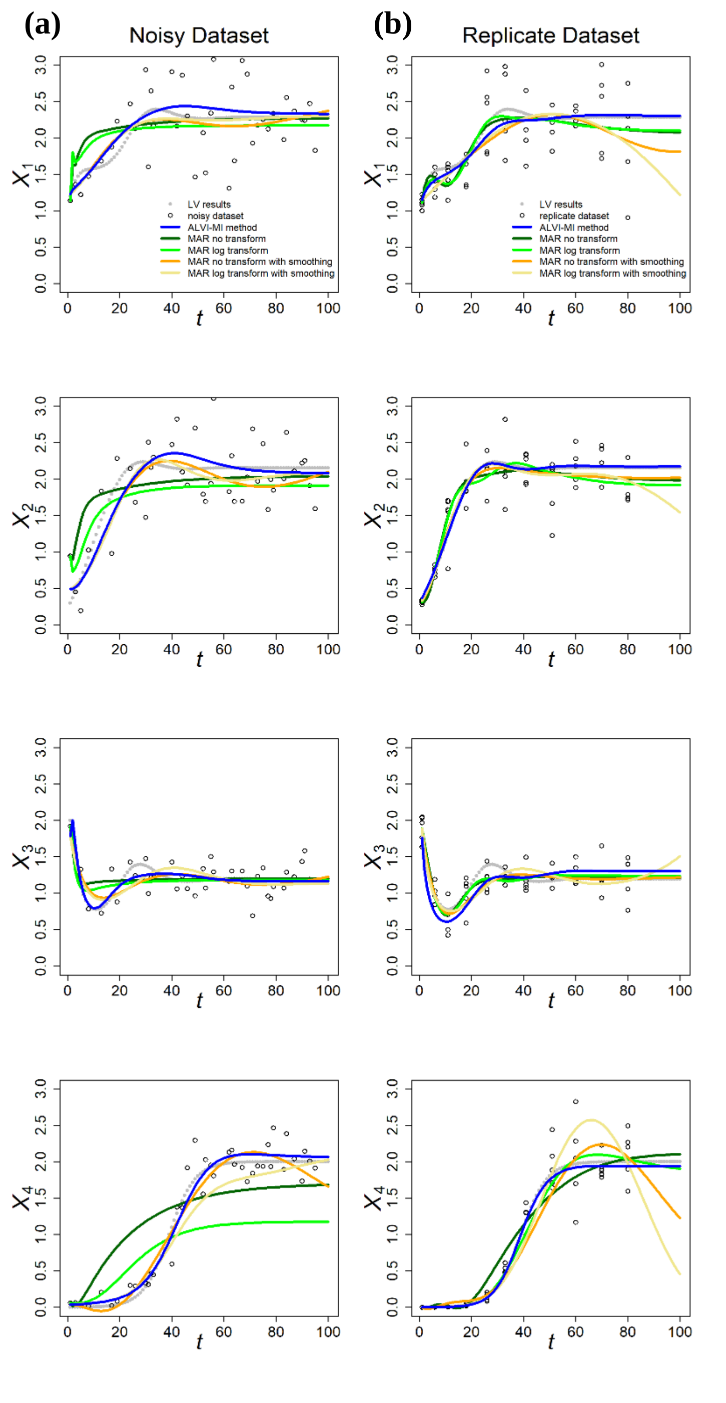

Figure S4: ALVI-MI and MARSS methods applied to noisy (a) and replicate (b) LV datasets with observational noise. Original synthetic data are shown as gray dots and data with added noise as black circles. ALVI results are presented in blue. True parameters and ALVI-MI estimates are presented in the Table. MAR estimates are presented in green, orange and yellow. Data and parameter estimates for MAR can be seen in Tables S2.1 to S2.4. SSEs for all fits are presented in Table S2.5.

Figure S5

		Initial Cond.	Noisy Est.
X_1	1.2	1.26251	1.23331
X_2	0.3	0.36268	0.30350
X_3	2	1.77750	1.96125
X_4	0.001	0.00100	0.00100

	Parameter	Noisy Est.	Replicate Est.
a_1	0.044	0.04307	0.04542
b_{11}	-0.08	-0.04771	-0.04521
b_{12}	0.02	0.01522	0.01475
b_{13}	0.08	0.03203	0.02897
b_{14}	0	-0.00300	-0.00452
a_2	0.216	0.23238	0.22659
b_{21}	-0.04	-0.06889	-0.04244
b_{22}	-0.08	-0.03794	-0.07417
b_{23}	0.04	0.00361	0.02407
b_{24}	0	0.00257	0.00062
a_3	0.116	0.17954	0.13408
b_{31}	-0.16	-0.19961	-0.19069
b_{32}	0.16	0.14998	0.17615
b_{33}	-0.08	-0.06055	-0.07134
b_{34}	0	0.01357	0.00579
a_4	0.2	0.18269	0.19742
b_{41}	0	0.01136	-0.00026
b_{42}	0	-0.00424	-0.00079
b_{43}	0	-0.00120	0.00135
b_{44}	-0.1	-0.09952	-0.09879
Degrees of Freedom		10,8,11,15	8,10,10,9

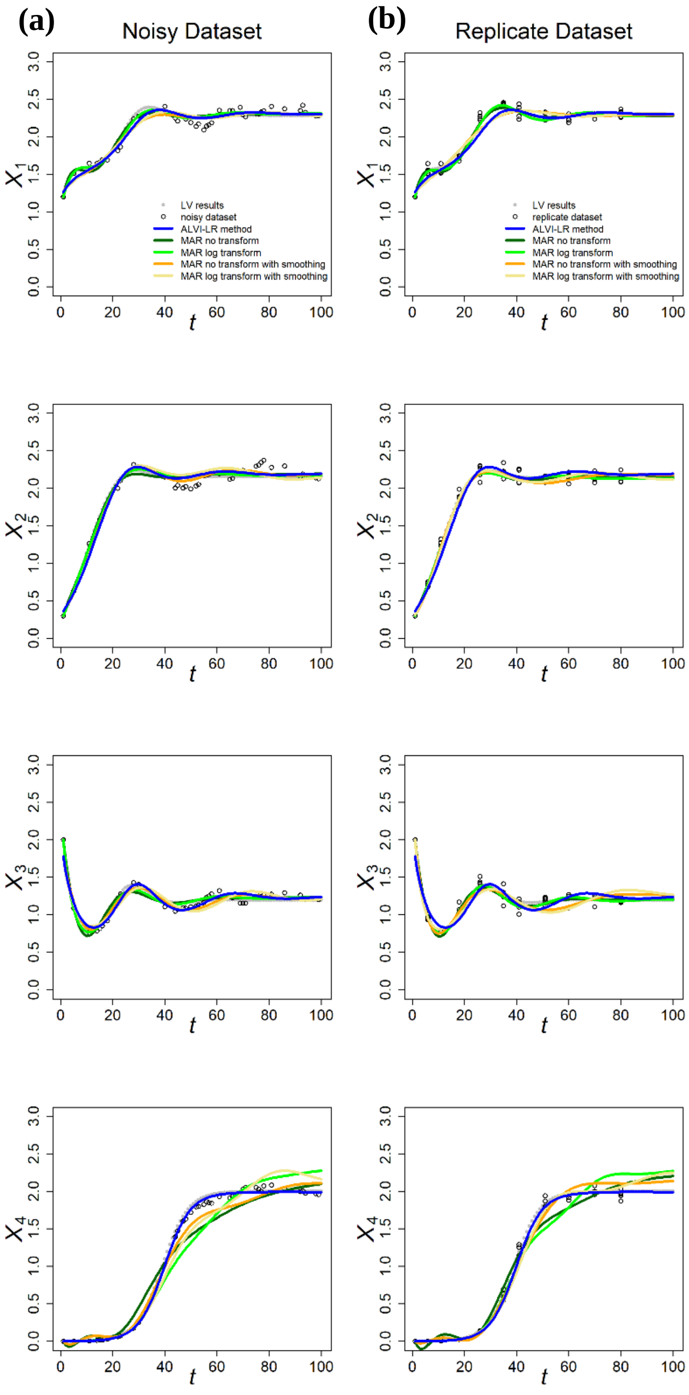

Figure S5: Results of ALVI-LR and MAR applied to the noisy and replicate datasets. Column a: Noisy dataset. Time courses of X_1, X_2, X_3 and X_4 were smoothed with 6, 11, 11 and 11DF-splines respectively. **Column b:** Replicate dataset. All variables were smoothed with 8DF-splines. The datasets are the same used in Figure 1 of the main text and in Tables S1.2 and S1.3 in the Supplements. MAR estimates are the same presented in Figure 1 and Tables S1.4 and S1.5. Noise level added is 20%.

Figure S6

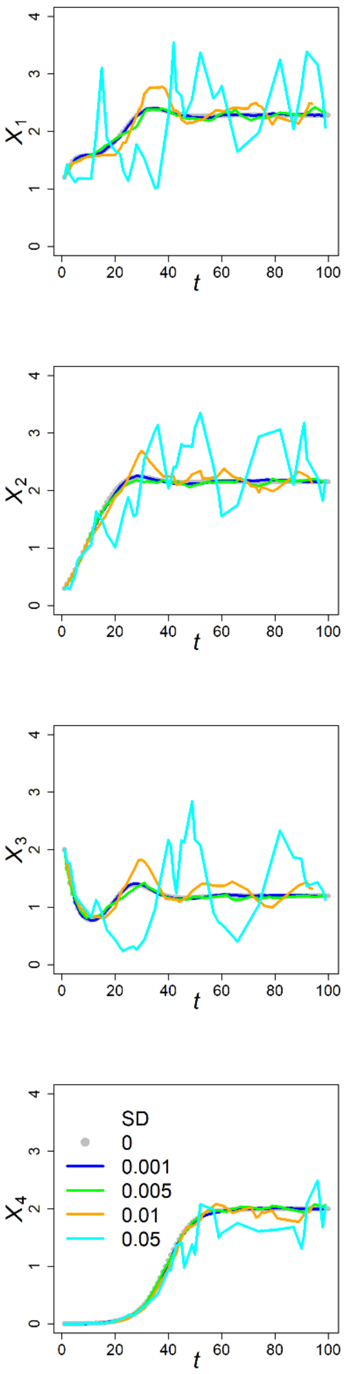

Figure S6 – Examples of different levels of process noise. Four datasets were generated by multiplying the discretized equations with a random normal noise with mean 1 and different standard deviations.

Figure S7

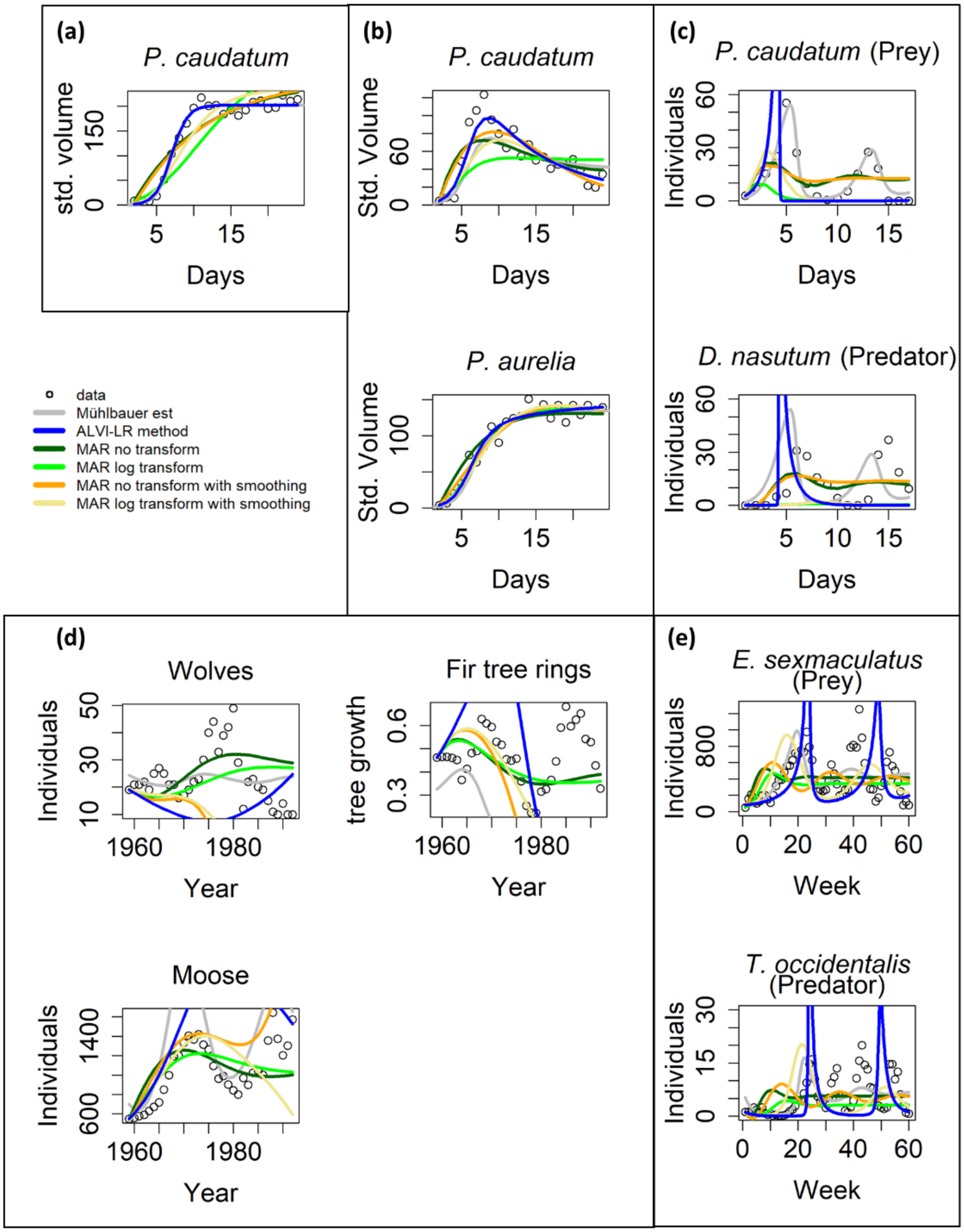

Figure S7 – Examples of experimental data analyzed with ALVI-LR and MAR. Black lines are estimates from Mühlbauer *et al.* (Mühlbauer et al., 2020). ALVI-LR estimates are represented as blue lines; corresponding parameter values can be seen in Table S4.2. MAR estimates are presented in green, orange and yellow. Parameter estimates are presented in Table S4.3. **a:** Standardized volume of *Paramecium caudatum* culture grown in monoculture (Gause, 1934). **b:** Standardized volume of *Paramecium caudatum* and *Paramecium aurelia* cultures grown together (Gause, 1934). **c:** Predator-prey interactions between *Didinium nasutum* and *Paramecium caudatum* grown in mixture (Gause, 1934). **d:** Multi-trophic dynamics for wolves, moose, and fir tree rings on Isle Royale from 1960 to 1994 (McLaren and Peterson, 1994). **e:** Predator-prey interactions between *Eotetranychus sexmaculatus* and *Typhlodromus occidentalis* in a spatially structured experiment (Huffaker et al., 1963).

Figure S8

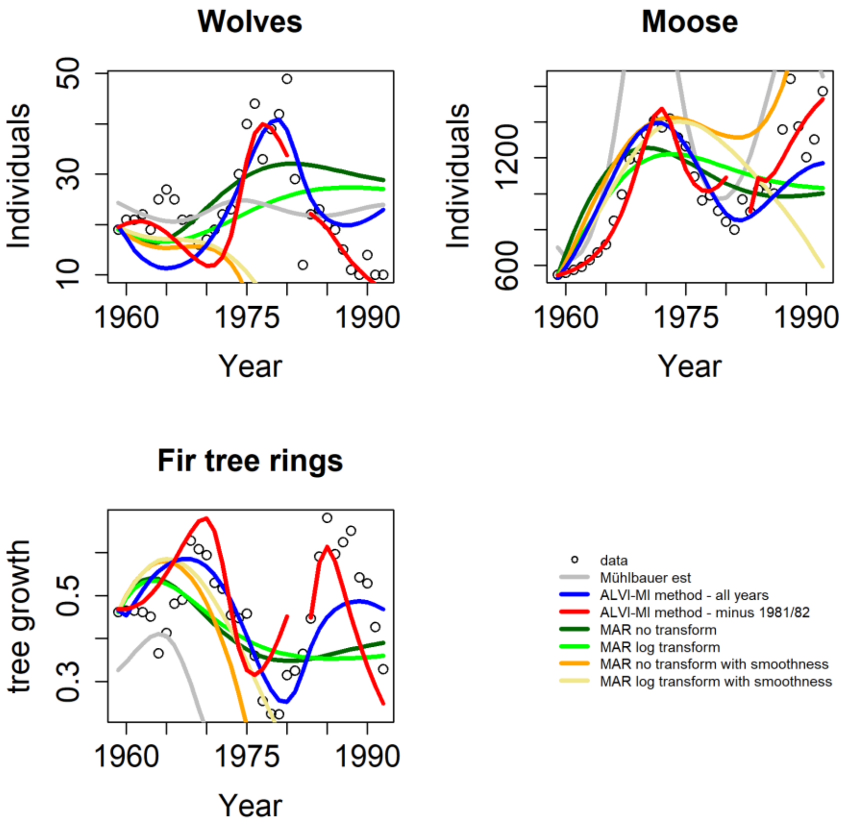

Figure S8 - Multi-trophic dynamics for wolves, moose, and fir trees on Isle Royale from 1960 to 1994, from McLaren & Peterson (McLaren and Peterson, 1994)**.** This panel is similar to Figure 2 d) but contains additional information. ALVI-MI estimates using all data are represented as blue lines. Red lines correspond to the estimates using ALVI-MI for two intervals, from 1959 to 1980 and form 1983 until the end of the series. This split was tested because around 1980 the wolves were exposed to a disease that drastically reduced their numbers, an event that dynamic models do not capture outside piecewise operation. MAR estimates are presented in green, orange and yellow. These estimates are the same as in Figure 4.

Figure S9

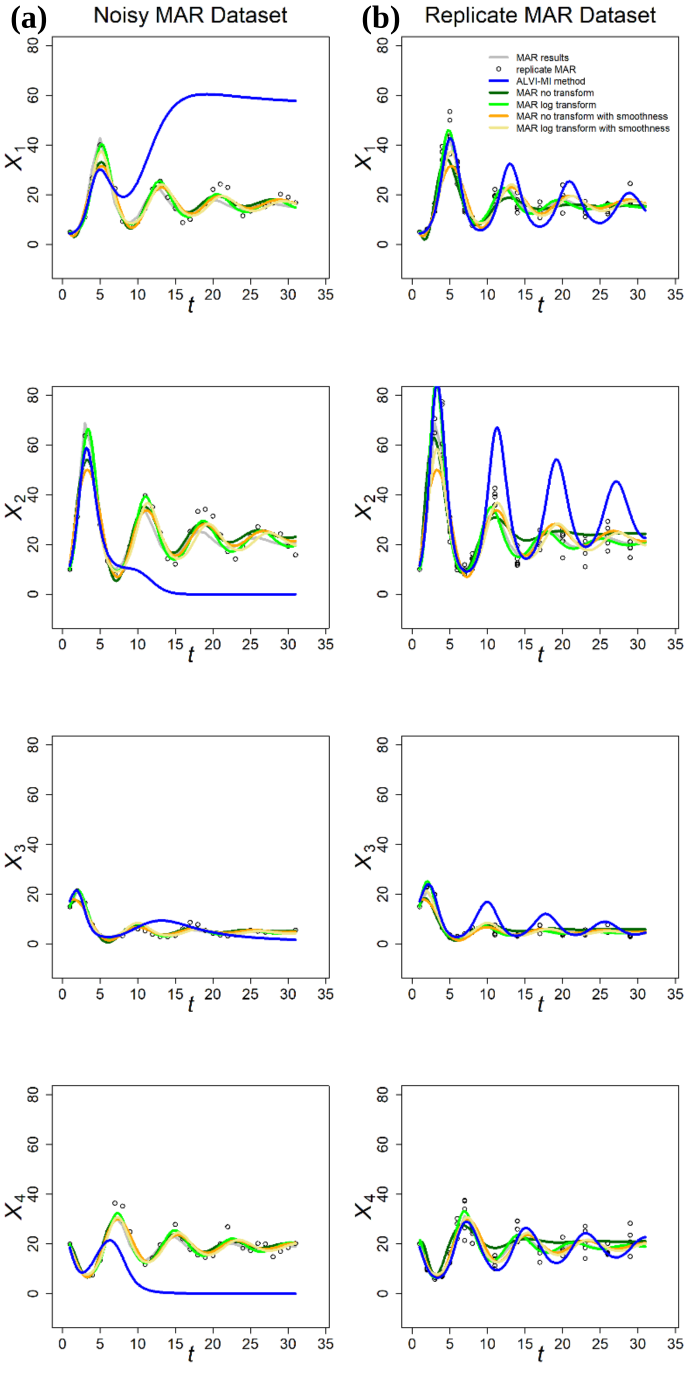

Figure S9: ALVI-MI and MAR applied to an alternative sample of the same data presented in Figure 2, but with slightly changed noise. Although the differences in noise are visually almost undetectable, very different results for the ALVI-MI fit are obtained if the same sample of spline points is used. However, if a new sample of spline points is determined, the fits are almost indistinguishable from the true trajectories (not shown). See Text for further explanations.

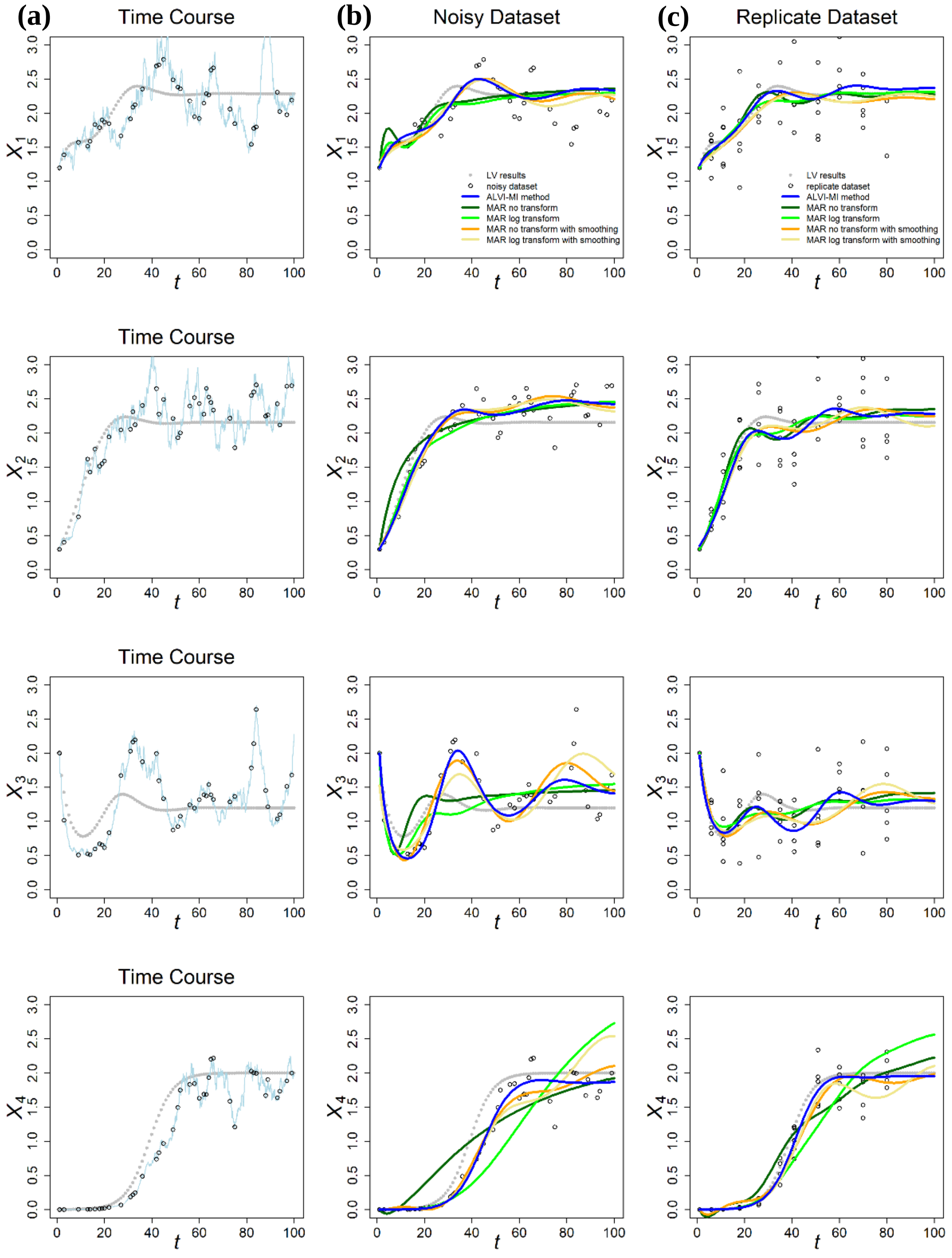

Figure S10: Time course used to originate the noisy dataset (a), ALVI-MI and MARSS methods applied to noisy (b) and replicate (c) LV datasets with process noise with increased standard variation. This Figure shows the same situation as Figure 1 in the main text with an increased standard variation for the process noise of 0.03. Original synthetic data are shown as gray dots and data with added noise as black circles. LV results are presented in blue. True parameters and LV estimates are presented in the Table. MAR estimates are presented in green, orange and yellow.

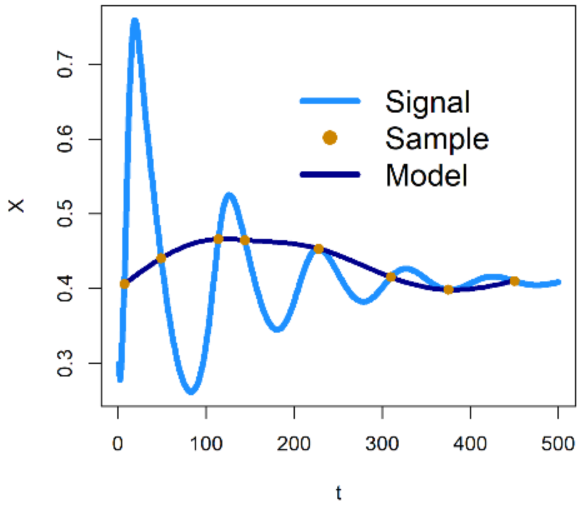

Figure S11: Smoothing example. Noise-free example demonstrating how smoothing can yield very misleading results.

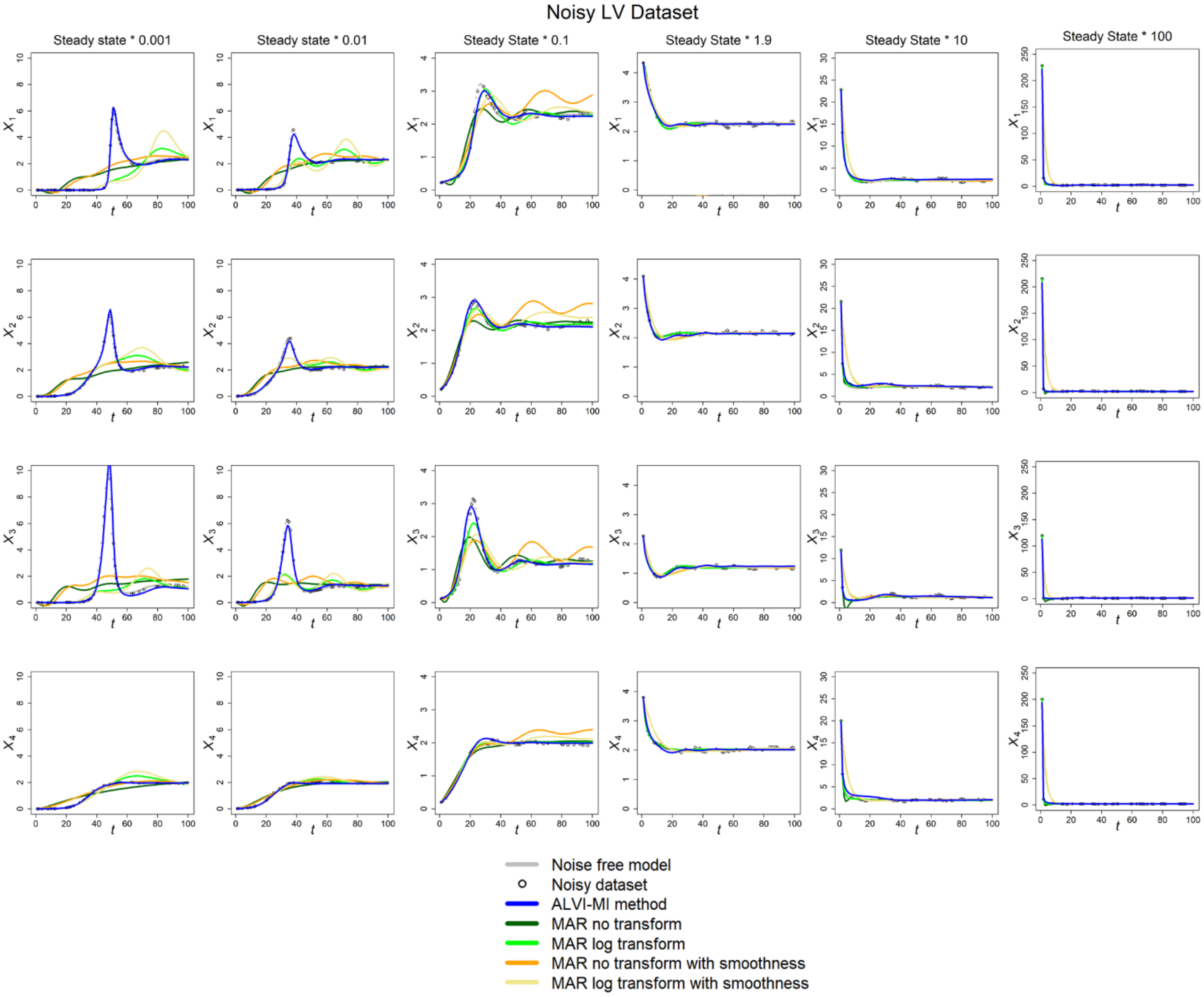

Figure S12: Artificial LV system with different initial conditions using random sampling. The different columns display the dynamics of the same system as used in Figure 1, but started with different values, which were chosen ratios of the system steady state. For each starting condition, forty random points were sampled and used to inform the estimation methods.

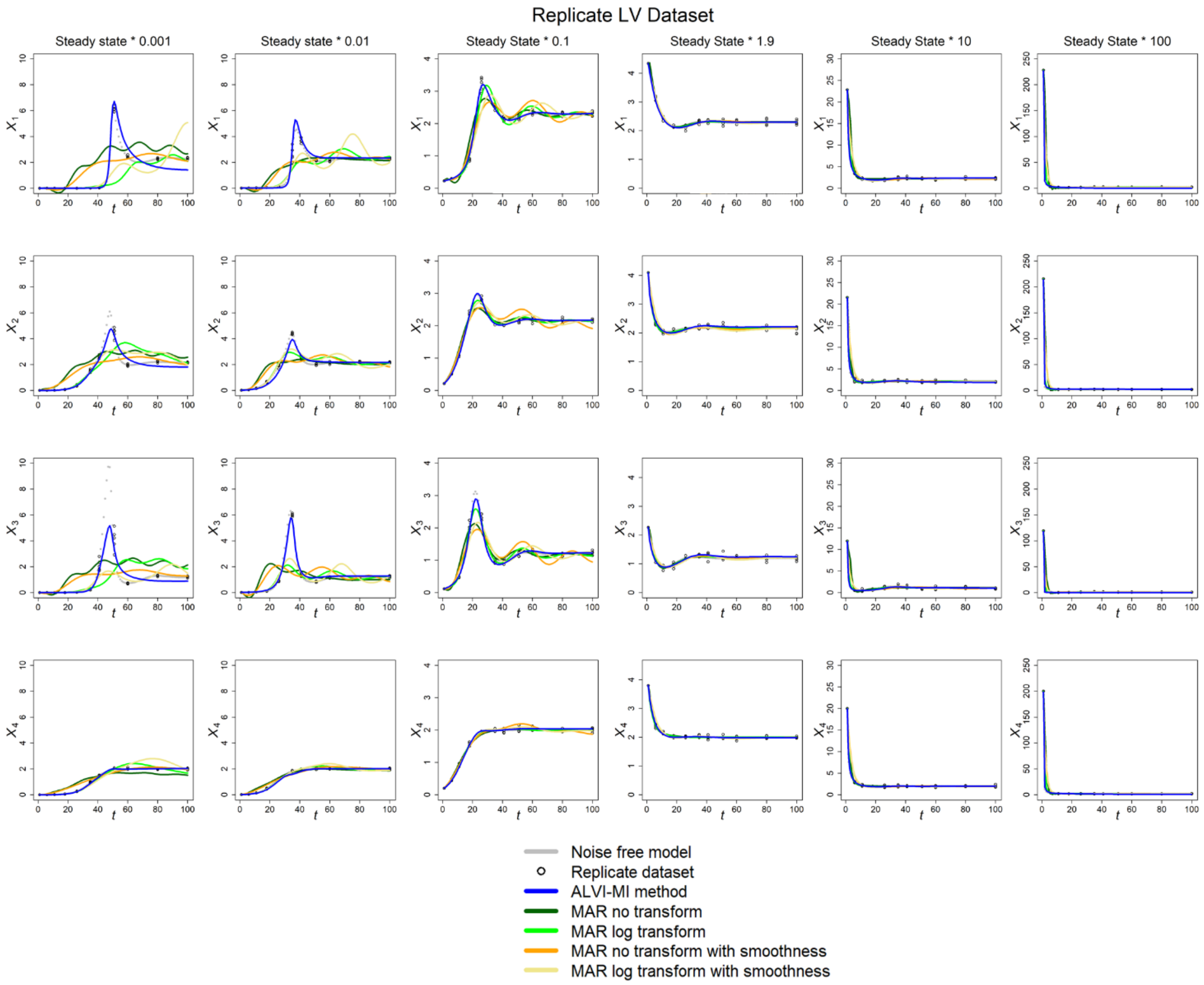

Figure S13: Artificial LV system with different initial conditions using replicate sampling. The different columns display the dynamics of the same system as used in Figure 1 but started with different values that were chosen as ratios of the system steady state. For each starting condition, the system was run five times and sampled at predetermined timepoints.

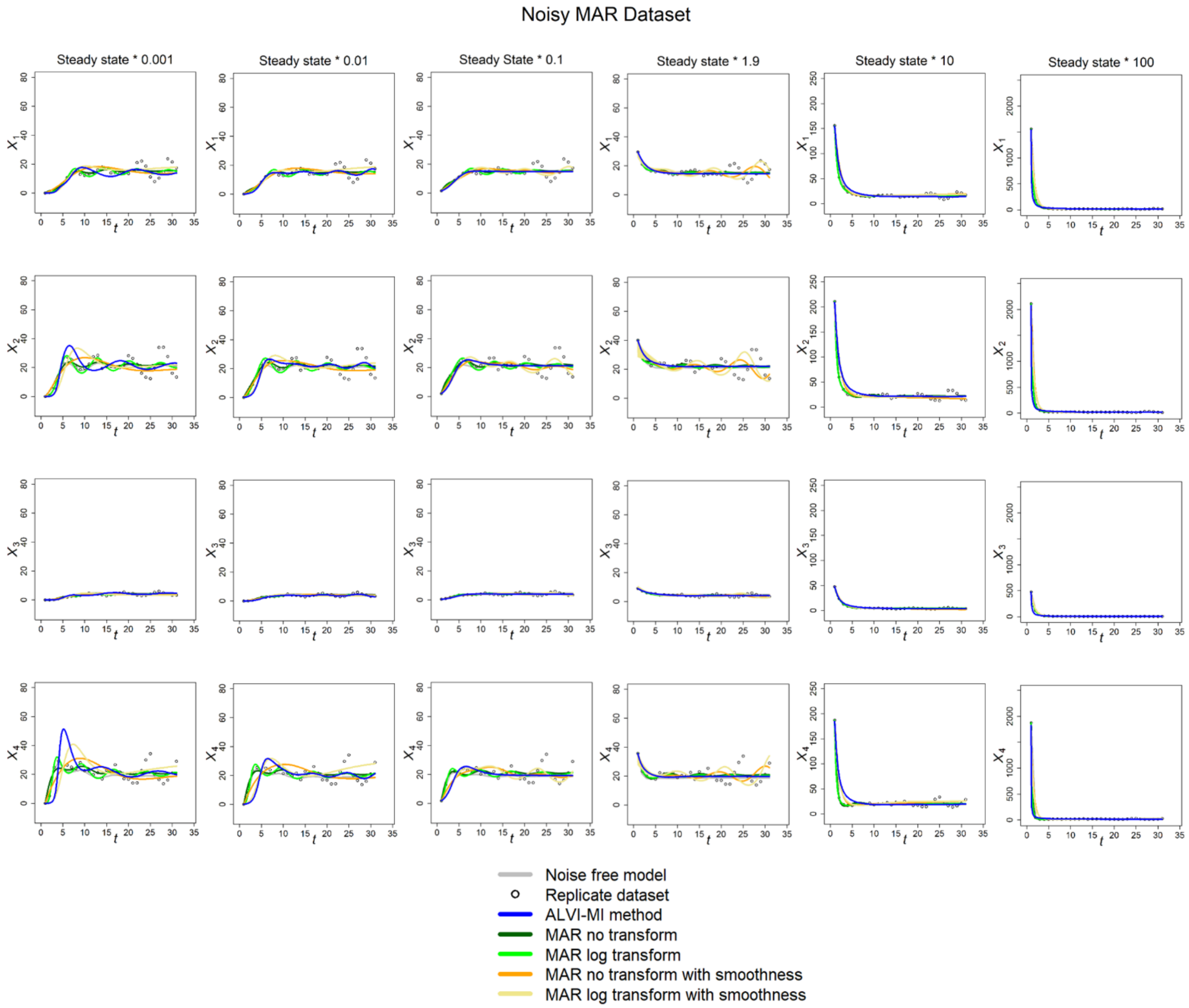

Figure S14: Artificial MAR system with different initial conditions, using random sampling. The different columns present the dynamics of the same system used in Figure 2 when started with different values that were chosen as ratios of the original system's steady state. For each starting condition, forty random points were sampled and used to inform the estimation methods.

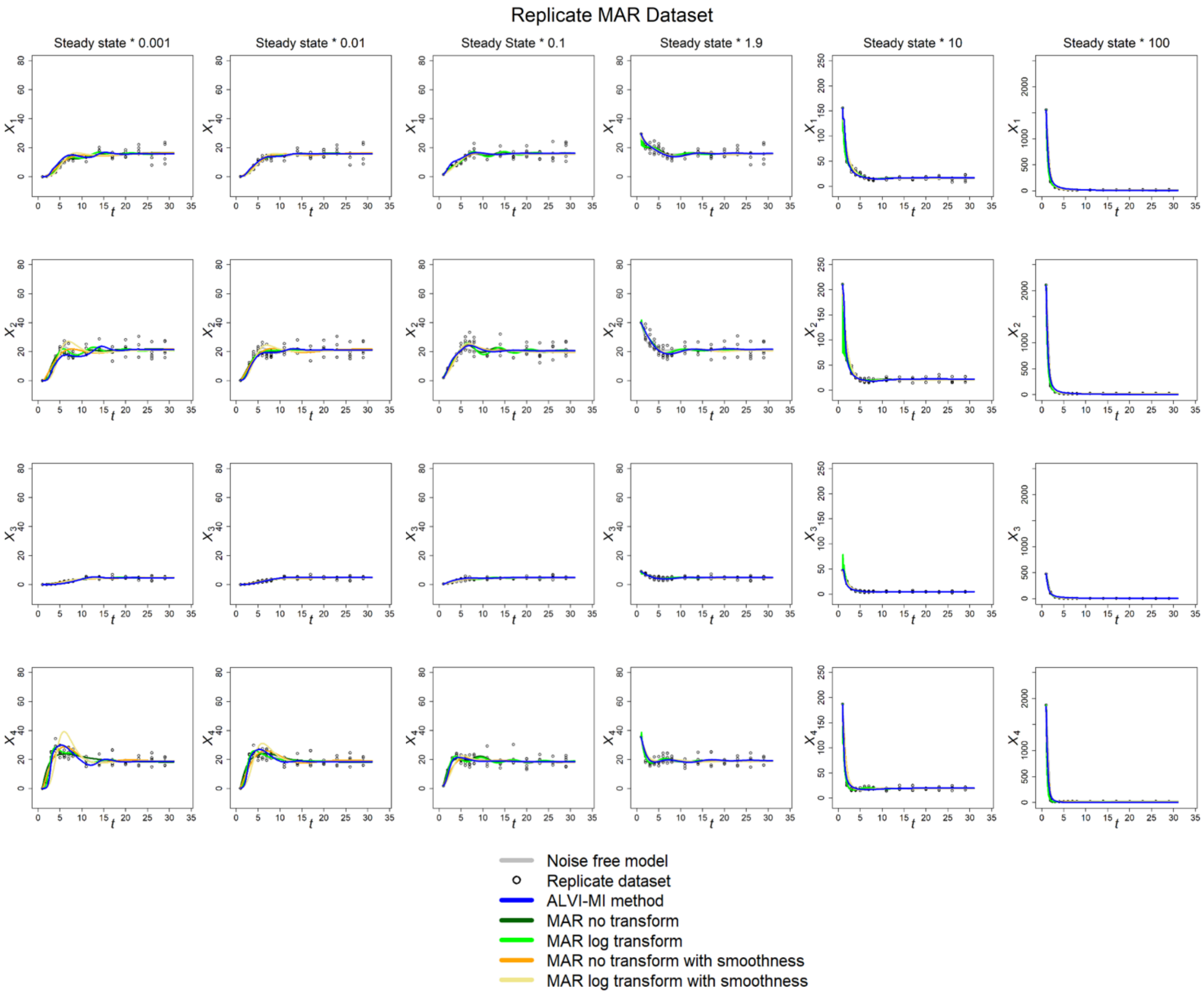

Figure S15: Artificial MAR system with different initial conditions, using replicate sampling. The different columns present the dynamics of the same system used in Figure 2 when started with different values that were chosen as ratios of the original system's steady state. For each starting condition, the system was run five times and sampled at predetermined timepoints.

5 Supplemental Tables

Table S1.1 – Synthetic LV data without noise. The data were generated with an LV system with four dependent variables with parameter values presented in Figure S1.

t	X_1	X_2	X_3	X_4
1	1.20000	0.30000	2.00000	0.00100
2	1.32083	0.37078	1.67253	0.00122
3	1.41112	0.44848	1.42395	0.00149
4	1.47488	0.53265	1.23448	0.00182
5	1.51746	0.62275	1.09051	0.00222
6	1.54431	0.71810	0.98219	0.00272
7	1.56033	0.81785	0.90227	0.00332
8	1.56959	0.92096	0.84535	0.00405
9	1.57534	1.02621	0.80746	0.00494
10	1.58006	1.13229	0.78565	0.00603
11	1.58566	1.23781	0.77778	0.00737
12	1.59356	1.34144	0.78227	0.00899
13	1.60486	1.44195	0.79788	0.01097
14	1.62038	1.53828	0.82360	0.01338
15	1.64076	1.62958	0.85846	0.01632
16	1.66648	1.71521	0.90140	0.01990
17	1.69783	1.79474	0.95118	0.02425
18	1.73496	1.86789	1.00625	0.02954
19	1.77779	1.93449	1.06475	0.03596
20	1.82599	1.99445	1.12450	0.04375
21	1.87894	2.04769	1.18311	0.05317
22	1.93567	2.09416	1.23810	0.06457
23	1.99490	2.13382	1.28715	0.07830
24	2.05502	2.16666	1.32828	0.09482
25	2.11427	2.19279	1.36011	0.11461
26	2.17079	2.21241	1.38190	0.13823
27	2.22286	2.22591	1.39369	0.16628
28	2.26899	2.23381	1.39615	0.19943
29	2.30811	2.23680	1.39047	0.23832
30	2.33961	2.23566	1.37821	0.28360
31	2.36336	2.23123	1.36102	0.33585
32	2.37966	2.22437	1.34057	0.39550
33	2.38913	2.21586	1.31834	0.46281
34	2.39266	2.20642	1.29562	0.53773
35	2.39123	2.19665	1.27343	0.61988
36	2.38587	2.18704	1.25253	0.70850
37	2.37758	2.17796	1.23345	0.80243
38	2.36727	2.16968	1.21655	0.90013
39	2.35575	2.16237	1.20201	0.99980
40	2.34368	2.15612	1.18987	1.09947
41	2.33162	2.15098	1.18009	1.19718
42	2.32001	2.14691	1.17257	1.29113
43	2.30917	2.14387	1.16713	1.37978
44	2.29935	2.14177	1.16358	1.46196
45	2.29070	2.14052	1.16170	1.53691
46	2.28329	2.14001	1.16126	1.60424
47	2.27717	2.14013	1.16203	1.66392
48	2.27231	2.14076	1.16377	1.71620
49	2.26866	2.14180	1.16626	1.76151
50	2.26613	2.14313	1.16930	1.80043
51	2.26462	2.14467	1.17270	1.83359
52	2.26400	2.14632	1.17627	1.86167
53	2.26415	2.14801	1.17987	1.88531
54	2.26494	2.14968	1.18337	1.90511
55	2.26623	2.15128	1.18668	1.92164
56	2.26790	2.15275	1.18970	1.93538
57	2.26982	2.15407	1.19237	1.94679
58	2.27189	2.15523	1.19467	1.95622
59	2.27400	2.15620	1.19658	1.96401
60	2.27609	2.15698	1.19809	1.97044
61	2.27808	2.15759	1.19921	1.97573

62	2.27991	2.15802	1.19998	1.98009
63	2.28156	2.15829	1.20042	1.98367
64	2.28299	2.15842	1.20058	1.98661
65	2.28418	2.15843	1.20049	1.98902
66	2.28515	2.15833	1.20021	1.99100
67	2.28588	2.15816	1.19977	1.99263
68	2.28640	2.15792	1.19922	1.99396
69	2.28673	2.15764	1.19859	1.99505
70	2.28688	2.15733	1.19793	1.99595
71	2.28688	2.15702	1.19726	1.99668
72	2.28675	2.15671	1.19660	1.99728
73	2.28653	2.15641	1.19598	1.99777
74	2.28623	2.15613	1.19541	1.99818
75	2.28588	2.15588	1.19491	1.99851
76	2.28551	2.15566	1.19448	1.99878
77	2.28512	2.15547	1.19411	1.99900
78	2.28473	2.15532	1.19383	1.99918
79	2.28436	2.15521	1.19361	1.99933
80	2.28402	2.15512	1.19346	1.99945
81	2.28371	2.15507	1.19337	1.99955
82	2.28344	2.15504	1.19333	1.99963
83	2.28322	2.15503	1.19334	1.99970
84	2.28303	2.15505	1.19338	1.99975
85	2.28289	2.15508	1.19346	1.99980
86	2.28279	2.15512	1.19355	1.99983
87	2.28272	2.15517	1.19367	1.99986
88	2.28269	2.15522	1.19378	1.99989
89	2.28269	2.15528	1.19391	1.99991
90	2.28270	2.15533	1.19403	1.99993
91	2.28274	2.15539	1.19414	1.99994
92	2.28279	2.15544	1.19425	1.99995
93	2.28286	2.15549	1.19434	1.99996
94	2.28292	2.15553	1.19442	1.99997
95	2.28299	2.15556	1.19449	1.99997
96	2.28306	2.15559	1.19455	1.99998
97	2.28313	2.15562	1.19459	1.99998
98	2.28320	2.15563	1.19462	1.99998
99	2.28325	2.15564	1.19464	1.99999
100	2.28330	2.15565	1.19465	1.99999

Table S1.2 – Noisy LV dataset with process noise. From the synthetic data in Table S1.1, forty values were selected with random gamma process noise with mode 1 and a standard deviation of 0.005.

t	X_1	X_2	X_3	X_4
1	1.20000	0.30000	2.00000	0.00100
5	1.51251	0.63358	1.08755	0.00219
11	1.64585	1.26200	0.76865	0.00697
14	1.64274	1.48105	0.78782	0.01288
15	1.66017	1.58414	0.81572	0.01529
16	1.69344	1.65454	0.85605	0.01860
18	1.69305	1.79765	0.92227	0.02729
22	1.81304	2.00073	1.22418	0.05985
23	1.86555	2.10382	1.27719	0.07099
28	2.23935	2.31639	1.42863	0.17424
30	2.27743	2.28900	1.40613	0.25200
40	2.40705	2.15132	1.10974	1.02776
44	2.25217	2.00371	1.04817	1.39308
45	2.21085	2.04131	1.07756	1.47266
47	2.27101	2.00634	1.09095	1.60541
48	2.24058	2.02473	1.08946	1.63323
50	2.19024	1.99448	1.10842	1.71833
52	2.13971	2.03247	1.11602	1.79507
53	2.17131	2.05273	1.13077	1.79976
55	2.09303	2.16683	1.16259	1.82728
56	2.14169	2.18090	1.23039	1.86148
57	2.15893	2.18827	1.24899	1.85011
58	2.18892	2.22929	1.27950	1.84045
61	2.35211	2.19343	1.32238	1.92892
65	2.35007	2.12164	1.27142	1.91159
66	2.34804	2.13106	1.22650	1.93078
69	2.39336	2.19446	1.15401	1.97738
70	2.29343	2.23809	1.15456	2.02717
71	2.26824	2.20654	1.15435	2.05222
75	2.31161	2.29433	1.24687	2.05090
76	2.30186	2.31163	1.25240	2.02968
77	2.33082	2.34493	1.24258	2.00202
78	2.34656	2.37479	1.26936	2.07087
81	2.40470	2.27890	1.27538	2.08506
86	2.37498	2.29640	1.29586	2.03397
92	2.34848	2.16038	1.26388	1.99799
93	2.42223	2.19219	1.22262	2.00513
94	2.32452	2.17029	1.20688	1.97529
98	2.27953	2.14108	1.21721	1.97030
99	2.28168	2.12702	1.20204	1.95857

Table S1.3 – Replicate LV dataset with process noise. 5 time series were created with random gamma process noise with mode 1 and a standard deviation of 0.005. 15 time points were selected, and their value recorded in the five time series.

t	X_1	X_2	X_3	X_4
1	1.20000	0.30000	2.00000	0.00100
1	1.20000	0.30000	2.00000	0.00100
1	1.20000	0.30000	2.00000	0.00100
1	1.20000	0.30000	2.00000	0.00100
1	1.20000	0.30000	2.00000	0.00100
6	1.50355	0.73064	0.93663	0.00264
6	1.55652	0.69938	0.96581	0.00282
6	1.51725	0.70634	0.95386	0.00269
6	1.53948	0.68626	0.94023	0.00267
6	1.64533	0.75280	1.00315	0.00268
11	1.55626	1.27193	0.78270	0.00695
11	1.52379	1.23059	0.77654	0.00745
11	1.53200	1.27776	0.74456	0.00742
11	1.51811	1.23210	0.78150	0.00773
11	1.64292	1.32126	0.78364	0.00726
18	1.67779	1.88860	1.02242	0.02728
18	1.71496	1.78942	0.97178	0.02929
18	1.71456	1.87836	1.03706	0.03082
18	1.74772	1.98872	1.17179	0.03071
18	1.73540	1.87148	1.07386	0.02678
26	2.26850	2.30238	1.51184	0.11832
26	2.08035	2.18324	1.40897	0.13217
26	2.24296	2.29280	1.41801	0.14215
26	2.32083	2.07833	1.32432	0.14052
26	2.22276	2.26929	1.43788	0.12060
35	2.42302	2.12743	1.11818	0.54949
35	2.45501	2.21424	1.23765	0.58778
35	2.45008	2.19947	1.29769	0.69399
35	2.43268	2.22595	1.24329	0.63315
35	2.43612	2.34112	1.41302	0.56372
41	2.23522	2.08018	1.00845	1.14479
41	2.35149	2.13545	1.10521	1.12536
41	2.27412	2.07173	1.16723	1.29210
41	2.38820	2.25877	1.17280	1.25602
41	2.43691	2.24197	1.21108	1.07008
51	2.31858	2.07923	1.18185	1.73076
51	2.22218	2.16262	1.23688	1.79832
51	2.32990	2.12956	1.11987	1.87016
51	2.24401	2.13366	1.22031	1.93983
51	2.26324	2.15439	1.20753	1.79423
60	2.30610	2.15613	1.24601	1.91992
60	2.27671	2.21367	1.24491	1.94921
60	2.19113	2.16943	1.09572	1.91549
60	2.22458	2.13073	1.26967	1.98787
60	2.23255	2.05976	1.09038	1.88321
70	2.29975	2.23259	1.24172	1.95636
70	2.24019	2.13489	1.26164	1.99328
70	2.28249	2.11836	1.22900	2.00258
70	2.30426	2.15297	1.26425	1.99650
70	2.30109	2.07601	1.10981	2.07773
80	2.26967	2.14344	1.22106	1.96005
80	2.36832	2.09322	1.18451	1.86882
80	2.33270	2.14371	1.17647	2.03363
80	2.32060	2.24480	1.24103	1.93923
80	2.25806	2.08439	1.16234	1.97468

Table S1.4 – MAR estimates for the noisy LV dataset with process noise in Figures 1 and S5.

	MAR without transformation	MAR with log transformation	MAR with smoothing	MAR with log transformation and smoothing
β_{11}	0.84413	0.85630	0.89175	0.91190
β_{21}	-0.12340	-0.09640	-0.22248	-0.14258
β_{31}	-0.18615	-0.38482	-0.25399	-0.58568
β_{41}	0.17772	0.27174	0.31566	0.45412
β_{12}	0.06341	0.02756	0.04618	0.02636
β_{22}	1.00207	0.91931	1.05986	0.96171
β_{32}	0.18648	0.17359	0.18256	0.18601
β_{42}	-0.04301	0.11562	-0.10247	0.09211
β_{13}	0.11908	0.08753	0.02948	0.02781
β_{23}	-0.02551	0.01969	-0.06283	-0.02235
β_{33}	0.85809	0.96271	0.89175	1.00641
β_{43}	-0.03045	0.04882	-0.03164	0.03135
β_{14}	-0.00301	-0.00101	-0.00149	-0.00176
β_{24}	0.00399	-0.00012	0.01615	0.00095
β_{34}	0.00745	0.00885	0.02280	0.01865
β_{44}	0.96062	0.93182	0.94077	0.92168
α_1	0.08044	0.08173	0.01027	0.00592
α_2	0.30362	0.13916	0.01778	0.01786
α_3	0.18074	0.18825	-0.00589	-0.00399
α_4	-0.19114	-0.26988	0.01995	0.07664
δ_1	0.00133	0.00023	0.00003	0.00000
δ_2	0.00092	0.00024	0.00005	0.00003
δ_3	0.00024	0.00015	0.00003	0.00006
δ_4	0.00104	0.00104	0.00028	0.00036

Table S1.5 – MAR estimates for the replicate LV dataset with process noise in Figures 1 and S5.

	MAR without transformation	MAR with log transformation	MAR with smoothing	MAR with log transformation and smoothing
β_{11}	0.85748	0.83902	0.92226	0.94965
β_{21}	-0.08820	-0.02322	-0.14563	-0.09104
β_{31}	-0.16769	-0.32656	-0.22421	-0.46501
β_{41}	0.21186	0.41978	0.31538	0.69449
β_{12}	0.05934	0.03292	0.03421	0.01789
β_{22}	0.99331	0.91148	1.02606	0.93669
β_{32}	0.18256	0.17803	0.20977	0.20265
β_{42}	-0.06143	0.08393	-0.11902	0.00759
β_{13}	0.12485	0.10438	0.00884	0.01254
β_{23}	-0.04732	-0.01476	-0.06818	-0.02801
β_{33}	0.86314	0.98782	0.83056	0.96753
β_{43}	-0.05509	0.01799	-0.02853	0.00199
β_{14}	-0.00478	-0.00052	-0.00747	-0.00326
β_{24}	-0.00760	-0.00367	0.00036	-0.00142
β_{34}	-0.00040	0.00356	0.01062	0.01049
β_{44}	0.96290	0.92774	0.95265	0.91845
α_1	0.05648	0.08922	0.01296	0.00749
α_2	0.28550	0.09089	0.02280	0.02393
α_3	0.15580	0.13414	-0.01004	-0.00642
α_4	-0.20024	-0.35593	0.02490	0.09494
δ_1	0.00013	0.00000	0.00001	0.00000
δ_2	0.00012	0.00004	0.00001	0.00000
δ_3	0.00021	0.00002	0.00008	0.00012
δ_4	0.00215	0.00084	0.00017	0.00002

Table S1.6 – Sum of squared errors (SSE) of data fits for noisy and replicate LV datasets with process noise with ALVI-LR (linear regression), ALVI-MI (matrix inversion) and four variants of the MAR methods.

	Noisy LV dataset					Replicate LV dataset				
	X_1	X_2	X_3	X_4	**Total**	X_1	X_2	X_3	X_4	**Total**
ALVI-LR	0.232	0.321	0.401	0.122	**1.076**	0.138	0.042	0.147	0.022	**0.349**
ALVI-MI	0.330	0.331	0.349	0.151	**1.160**	0.031	0.060	0.107	0.029	**0.226**
MAR	0.109	0.101	0.163	3.301	**3.674**	0.080	0.050	0.096	1.287	**1.513**
MAR log transform	0.074	0.080	0.109	5.071	**5.335**	0.053	0.080	0.046	1.215	**1.395**
MAR with smoothing	0.346	0.341	0.316	1.636	**2.638**	0.302	0.043	0.202	0.846	**1.394**
MAR log transform with smoothing	0.347	0.498	0.595	3.577	**5.017**	0.289	0.093	0.445	0.655	**1.481**

Table S1.7 – Sum of squared errors (SSE) of data fits for noisy and replicate LV datasets with process noise with increased standard deviation (0.03) for ALVI-LR (linear regression), ALVI-MI (matrix inversion) and four variants of the MAR methods.

	Noisy LV dataset					Replicate LV dataset				
	X_1	X_2	X_3	X_4	**Total**	X_1	X_2	X_3	X_4	**Total**
ALVI-LR	1.302	4.460	11.758	5.426	**22.946**	0.528	2.284	2.707	0.648	**6.167**
MAR	1.060	4.243	4.807	11.031	**21.141**	0.653	2.261	2.375	4.304	**9.593**
MAR log transform	1.060	4.357	6.257	27.499	**39.173**	0.761	1.201	1.614	11.182	**14.759**
MAR with smoothing	1.539	6.086	14.868	6.461	**28.955**	0.880	1.810	2.904	2.246	**7.839**
MAR log transform with smoothing	1.564	5.271	16.069	11.327	**34.231**	0.840	1.664	4.265	3.858	**10.627**

Table S2.1 - Noisy LV dataset with observational noise. From the synthetic data in Table S1.1, forty values were selected and random normal noise was added with mean 0 and a standard deviation equal to 20% of each variable mean.

t	X_1	X_2	X_3	X_4
1	1.14111	0.94716	1.92175	0.06256
3	1.36364	0.45349	1.45431	0.06645
5	1.22283	0.19582	1.33123	0.03641
8	1.47489	1.02716	0.78493	0.01857
13	1.68296	1.83783	0.72635	0.20645
17	1.87602	0.98039	1.33307	0.02590
19	2.23270	2.28656	0.88182	0.08578
24	2.46929	2.14703	1.42853	0.30301
26	2.12740	1.68427	1.24384	0.29370
30	2.93876	1.47752	1.47634	0.33315
31	1.60470	2.50979	0.99567	0.31345
32	2.64561	2.16238	1.21792	0.46671
33	2.16612	2.30080	1.25219	0.44936
40	2.90825	2.47699	1.42807	0.59721
42	2.16482	2.82494	1.06154	1.37928
44	2.86258	2.10103	1.23401	1.37031
46	2.30199	1.92131	1.06248	1.91622
49	1.46650	2.70250	0.96520	2.29495
52	2.07311	1.79577	1.33918	1.55672
53	1.52180	1.69359	1.07106	2.03041
55	2.34261	1.94310	1.50543	1.95215
56	3.07809	3.11285	1.29445	1.80656
62	1.30983	1.83120	1.16429	2.13265
63	2.70020	2.32062	1.30813	2.15788
64	1.68571	1.69570	1.19108	1.96368
67	3.06508	1.69727	1.30761	1.92002
69	2.87653	2.02694	1.10203	1.72796
71	1.92870	2.69214	0.69211	1.84299
73	2.17466	1.97223	1.23306	1.94046
75	2.25649	2.48677	1.35097	1.93681
77	2.12334	1.58311	0.96197	2.23364
78	3.38719	1.99818	0.93052	1.93376
79	2.33980	1.85041	1.09020	2.46553
83	1.97877	2.01006	1.07131	1.89393
84	2.55545	2.64187	1.29245	2.38731
87	2.36884	2.11299	1.22538	2.03970
90	2.25285	2.22309	1.43866	1.73292
91	2.24325	2.25534	1.58509	2.14186
93	2.47453	1.91424	1.19010	2.00689
95	1.82853	1.59450	1.18877	1.91223

Table S2.2 - Replicate LV dataset with observational noise. 11 points were selected from the synthetic data in Table S1.1. For each time point, five observations were created by multiplying the original value by a normal random variable with mean 1 and standard deviation 0.2.

t	X_1	X_2	X_3	X_4
1	1.079474	0.307892	1.968433	0.001177
1	1.228073	0.319118	1.767284	0.001143
1	1.001938	0.278408	2.035955	0.001019
1	1.151608	0.34439	2.049352	0.000994
1	1.106675	0.330651	1.634474	0.001462
6	1.409001	0.827832	1.033651	0.003136
6	1.292779	0.655128	0.840712	0.002841
6	1.186731	0.753583	0.964293	0.003671
6	1.501709	0.702128	0.846646	0.002595
6	1.600803	0.778034	1.191477	0.003243
11	1.553429	1.585188	0.501395	0.008283
11	1.420026	1.565142	0.721249	0.009309
11	1.599542	0.772725	0.708241	0.004804
11	1.642382	1.707548	0.424371	0.00881
11	1.142047	1.689577	0.992655	0.00613
18	1.644046	1.84217	0.929999	0.044789
18	1.780007	1.601518	1.134646	0.030728
18	1.710696	1.833336	1.096591	0.023249
18	1.331607	2.483739	0.591253	0.029612
18	1.357593	1.968956	1.2092	0.017282
26	2.560157	2.19029	1.010075	0.084837
26	2.478865	2.142542	1.441704	0.160821
26	2.920662	2.166496	1.227926	0.177711
26	1.783109	1.700208	1.23534	0.205836
26	1.809358	2.395388	1.056138	0.105769
33	2.230052	2.81995	1.194645	0.540826
33	1.692464	2.038453	1.113628	0.428625
33	2.981684	2.168246	1.363852	0.486374
33	2.095491	1.58247	1.231077	0.474691
33	2.875616	2.102594	1.238574	0.612312
41	1.970818	2.333379	1.04227	1.296557
41	1.611026	1.927812	1.114056	1.438404
41	2.112647	2.279129	1.081485	0.99357
41	2.652937	1.953001	1.498288	1.30327
41	2.261238	2.34695	1.170614	1.306394
51	2.079631	1.228253	1.209477	2.075646
51	1.819921	1.666988	1.070121	1.644326
51	2.454389	2.202211	1.41537	2.439964
51	2.256488	2.134285	1.231388	1.709964
51	2.213276	2.102253	1.234634	1.884561
60	2.165804	2.18245	1.155609	2.283614
60	2.279157	1.885294	1.137585	1.698353
60	2.36829	2.52211	1.349542	2.049815
60	2.234632	2.281866	1.184983	1.165772
60	2.439225	1.996245	1.501995	2.82506
70	1.719859	2.411846	1.227642	1.786889
70	2.570555	2.462909	1.1756	1.878108
70	1.790253	1.887747	1.142104	1.895824
70	2.723166	2.042576	1.65201	1.8243
70	3.007495	2.227207	0.93787	2.226255
80	2.296889	2.001374	1.396972	2.20474
80	2.749139	1.714839	1.059392	1.594539
80	0.9041	2.298373	1.489557	2.267917
80	1.676455	1.788501	0.766479	2.491924
80	2.131265	1.738182	1.403356	1.898047

Table S2.3 - MAR estimates for the noisy dataset with observational noise in Figure S4.

	MAR without transformation	MAR with log transformation	MAR with smoothing	MAR with log transformation and smoothing
β_{11}	-0.09089	-0.03360	1.06000	1.02000
β_{21}	0.27209	0.43560	0.13800	0.07940
β_{31}	-0.21012	-0.43570	0.07490	-0.10500
β_{41}	0.12619	0.42840	-0.33900	-0.22100
β_{12}	0.43295	0.24860	-0.05360	-0.01990
β_{22}	0.47619	0.62610	0.91000	0.94700
β_{32}	0.18150	0.17100	0.01730	0.12000
β_{42}	0.00788	0.23190	0.22900	0.19400
β_{13}	-0.20530	-0.01010	-0.01980	-0.01510
β_{23}	-0.28009	-0.33410	-0.07090	-0.07300
β_{33}	0.50995	0.57920	0.86700	0.90900
β_{43}	-0.00869	-0.02950	-0.03190	0.04240
β_{14}	0.07772	0.00960	-0.00954	-0.00439
β_{24}	0.06929	0.02720	-0.02780	-0.01270
β_{34}	0.00957	0.01530	-0.00911	-0.00807
β_{44}	0.93364	0.88740	0.98800	0.95600
α_1	1.70925	0.63980	0.01030	0.00614
α_2	0.67025	-0.04340	0.01430	0.01390
α_3	0.67931	0.29480	-0.00571	-0.00386
α_4	-0.17950	-0.45920	0.02010	0.04190
δ_1	0.22184	0.05270	0.00005	0.00001
δ_2	0.19571	0.07520	0.00014	0.00003
δ_3	0.01924	0.01480	0.00049	0.00028
δ_4	0.04708	0.07570	0.00005	0.00010

Table S2.4 - MAR estimates for the replicate dataset with observational noise in Figure S4.

	MAR without transformation	MAR with log transformation	MAR with smoothing	MAR with log transformation and smoothing
β_{11}	0.77800	0.76500	1.01000	1.07000
β_{21}	0.07760	0.21000	0.00367	0.07720
β_{31}	-0.08920	-0.31200	-0.01630	-0.10700
β_{41}	0.09140	0.13400	0.23200	0.38400
β_{12}	0.13500	0.11600	-0.00781	-0.00292
β_{22}	0.87500	0.80500	0.93600	0.88300
β_{32}	0.14900	0.22500	0.10400	0.14800
β_{42}	-0.00518	0.17400	-0.08900	0.06510
β_{13}	0.24800	0.22200	-0.00982	-0.00328
β_{23}	-0.20400	-0.18300	-0.10300	-0.05940
β_{33}	0.94400	1.13000	0.86500	0.98300
β_{43}	0.01090	0.17500	-0.02880	0.04240
β_{14}	-0.02600	-0.01100	-0.02170	-0.00991
β_{24}	-0.00159	-0.00250	-0.00530	-0.00583
β_{34}	-0.00022	-0.00299	0.00004	-0.00317
β_{44}	0.97200	0.93000	0.96400	0.93000
α_1	0.02400	0.01470	0.00925	0.00610
α_2	0.02610	0.03690	0.01980	0.02190
α_3	-0.02240	-0.01470	-0.00880	-0.00573
α_4	0.02160	0.10700	0.02500	0.09300
δ_1	0.00557	0.00089	0.00008	0.00001
δ_2	0.00243	0.00073	0.00037	0.00009
δ_3	0.00001	0.00004	0.00024	0.00043
δ_4	0.00559	0.00267	0.00029	0.00022

Table S2.5 – Sum of squared errors (SSE) of data fits for noise and replicate LV datasets with observational noise with ALVI-LR (linear regression), ALVI-MI (matrix inversion) and four variants of the MAR methods for the data presented in Figure S4.

	Shown in	ALVI-LR	ALVI-MI	MAR	MAR logTrans	MAR with smoothing	MAR with log and smoothing	Mühlbauer *et al.*	Test
Noisy LV dataset	Fig. 1a	9.009	**4.362**	37.31	53.31	7.364	8.260		*
Replicate LV dataset	Fig. 1b	11.41	**2.953**	6.247	5.439	13.87	42.83		*

Table S3.1 – Initial conditions, parameter values and estimates for a four-variable LV system that converges to a stable steady state, as presented in Figure 3a.

	Initial Condition	Estimate
X_1	1.2	1.20993
X_2	0.3	0.31991
X_3	2	1.82146
X_4	0.001	0.00104

	True Parameter Value	ALVI-MI
a_1	0.044	0.04398
b_{11}	-0.08	-0.07995
b_{12}	0.02	0.01997
b_{13}	0.08	0.07999
b_{14}	0	-0.00001
a_2	0.216	0.21599
b_{21}	-0.04	-0.04000
b_{22}	-0.08	-0.08001
b_{23}	0.04	0.04002
b_{24}	0	0.00000
a_3	0.116	0.11600
b_{31}	-0.16	-0.15993
b_{32}	0.16	0.15998
b_{33}	-0.08	-0.08007
b_{34}	0	-0.00001
a_4	0.2	0.20002
b_{41}	0	-0.00001
b_{42}	0	0.00000
b_{43}	0	0.00000
b_{44}	-0.1	-0.10000

	MAR	MAR with log transformation
β_{11}	0.89300	0.88800
β_{21}	-0.07190	0.08510
β_{31}	-0.17500	-0.43000
β_{41}	0.19200	0.42800
β_{12}	0.04840	0.03110
β_{22}	0.99000	0.91100
β_{32}	0.16800	0.18000
β_{42}	-0.05440	0.11000
β_{13}	0.06600	0.06700
β_{23}	-0.07540	-0.06800
β_{33}	0.91100	1.03000
β_{43}	-0.00639	0.03510
β_{14}	-0.00719	-0.00229
β_{24}	-0.00739	-0.00763
β_{34}	0.00126	0.00751
β_{44}	0.96300	0.92400
α_1	0.07320	0.05740
α_2	0.28900	0.01420
α_3	0.14200	0.20700
α_4	-0.22800	-0.38500
δ_1	0.00014	0.00006
δ_2	0.00004	0.00031
δ_3	0.00064	0.00020
δ_4	0.00032	0.00032

Table S3.2 – Initial conditions, parameter values and estimates for a four-variable LV system exhibiting damped oscillations as presented in Figure 3b.

	Initial Condition	Estimate
X_1	0.3	0.3
X_2	0.3	0.3
X_3	0.4	0.4
X_4	0.6	0.6

	True Parameter Value	ALVI-MI
a_1	0.3	0.30245
b_{11}	-0.3	-0.30328
b_{12}	-0.27	-0.27098
b_{13}	-0.6	-0.60467
b_{14}	-0.045	-0.04708
a_2	0.4	0.40299
b_{21}	0.2	0.19592
b_{22}	-0.4	-0.40118
b_{23}	-0.4	-0.40578
b_{24}	-0.6	-0.60250
a_3	0.7	0.71519
b_{31}	-2.38	-2.39901
b_{32}	0.35	0.34344
b_{33}	-2.8	-2.82806
b_{34}	0.35	0.33650
a_4	0.6	0.60791
b_{41}	-0.96	-0.97003
b_{42}	-0.24	-0.24338
b_{43}	-0.96	-0.97466
b_{44}	-0.6	-0.60704

	MAR	MAR with log transformation
β_{11}	1.05000	0.98800
β_{21}	0.14700	0.01610
β_{31}	-0.25300	-0.07880
β_{41}	-0.23100	-0.05360
β_{12}	-0.08350	-0.11600
β_{22}	0.88800	0.92200
β_{32}	0.04880	-0.03410
β_{42}	-0.00228	-0.07260
β_{13}	0.03660	-0.04410
β_{23}	-0.07340	-0.06540
β_{33}	0.71200	0.89700
β_{43}	-0.20500	-0.02050
β_{14}	-0.10400	-0.01220
β_{24}	-0.12800	-0.03300
β_{34}	0.03690	-0.14100
β_{44}	0.96100	0.88100
α_1	0.04710	-0.22200
α_2	0.08150	-0.17700
α_3	0.06750	-0.56600
α_4	0.08660	-0.38800
δ_1	0.00000	0.00004
δ_2	0.00002	0.00016
δ_3	0.00015	0.00271
δ_4	0.00015	0.00065

Table S3.3 – Initial conditions, parameter values and estimates for a four-variable LV system initially displaying erratic oscillations, but then converging to a limit cycle as presented in Figure 3c.

	Initial Condition	Estimate
X_1	0.3	0.3
X_2	0.3	0.3
X_3	0.4	0.4
X_4	0.6	0.6

	True Parameter Value	ALVI-MI
a_1	1	0.98743
b_{11}	-1	-0.99101
b_{12}	-1.09	-1.08083
b_{13}	-1.52	-1.51341
b_{14}	0	0.01346
a_2	0.72	0.72230
b_{21}	0	-0.00274
b_{22}	-0.72	-0.72223
b_{23}	-0.3168	-0.31966
b_{24}	-0.9792	-0.97942
a_3	1.53	1.53466
b_{31}	-3.672	-3.67283
b_{32}	0	-0.00071
b_{33}	-1.53	-1.52615
b_{34}	-0.7191	-0.73207
a_4	1.27	1.27361
b_{41}	-1.5367	-1.53884
b_{42}	-0.6477	-0.64952
b_{43}	-0.4445	-0.44519
b_{44}	-1.27	-1.27581

	MAR	MAR with log transformation
β_{11}	0.87600	0.94673
β_{21}	0.26700	0.19269
β_{31}	-0.11100	-0.77772
β_{41}	-0.17600	-0.18648
β_{12}	-0.13600	-0.11408
β_{22}	0.90900	0.88847
β_{32}	0.03980	0.59990
β_{42}	0.06220	0.11789
β_{13}	-0.44100	0.00528
β_{23}	0.07280	0.01411
β_{33}	0.86500	0.93625
β_{43}	-0.03720	-0.00153
β_{14}	0.36500	0.00392
β_{24}	-0.19100	-0.17211
β_{34}	-0.02410	0.33444
β_{44}	0.93800	0.97758
α_1	0.02540	-0.12418
α_2	0.01980	-0.00507
α_3	0.03310	-0.28038
α_4	0.04680	-0.16530
δ_1	0.00001	0.00057
δ_2	0.00001	0.00012
δ_3	0.00013	0.00112
δ_4	0.00005	0.00022

Table S3.4 – Initial conditions, parameter values and estimates for a four-variable LV system displaying sustained oscillations as presented in Figure 3d.

	Initial Condition	Estimate
X_1	0.3	0.3
X_2	0.3	0.3
X_3	0.4	0.4
X_4	0.6	0.6

	True Parameter Value	ALVI-MI
a_1	0.3	0.27831
b_{11}	-0.3	-0.30250
b_{12}	-0.27	-0.25033
b_{13}	-0.6	-0.59705
b_{14}	-0.045	-0.00606
a_2	0.4	0.36675
b_{21}	0.2	0.19193
b_{22}	-0.4	-0.36840
b_{23}	-0.4	-0.40045
b_{24}	-0.6	-0.53672
a_3	0.7	0.64222
b_{31}	-2.38	-2.37532
b_{32}	0.35	0.39856
b_{33}	-2.45	-2.42900
b_{34}	0.35	0.44414
a_4	0.6	0.55927
b_{41}	-0.96	-0.96318
b_{42}	-0.24	-0.20356
b_{43}	-0.96	-0.95279
b_{44}	-0.3	-0.22817

	MAR	MAR with log transformation
β_{11}	0.96100	1.02036
β_{21}	0.17900	0.10195
β_{31}	-0.15100	-0.09094
β_{41}	-0.20000	-0.04845
β_{12}	-0.07430	-0.05021
β_{22}	0.94200	0.98452
β_{32}	-0.05210	-0.02708
β_{42}	-0.01790	-0.03530
β_{13}	-0.09270	0.00210
β_{23}	0.00417	-0.00943
β_{33}	0.82100	1.05860
β_{43}	-0.15000	0.02181
β_{14}	-0.00981	0.02438
β_{24}	-0.10400	-0.00099
β_{34}	-0.12500	-0.32180
β_{44}	0.96300	0.89997
α_1	0.05890	0.00507
α_2	0.01900	0.11510
α_3	0.13300	-0.41484
α_4	0.09470	-0.17804
δ_1	0.00003	0.00018
δ_2	0.00010	0.00101
δ_3	0.00014	0.00935
δ_4	0.00008	0.00056

Table S3.5 – Initial conditions, parameter values and estimates for a four-variable LV system displaying deterministic chaos (chaos 1) as presented in Figure 3e.

	Initial Condition	Estimate
X_1	0.3	0.3
X_2	0.3	0.3
X_3	0.4	0.4
X_4	0.6	0.6

	True Parameter Value	ALVI-MI
a_1	1	1.01561
b_{11}	-1	-1.01294
b_{12}	-1.09	-1.10110
b_{13}	-1.52	-1.52409
b_{14}	0	-0.01741
a_2	0.72	0.72904
b_{21}	0	-0.00777
b_{22}	-0.72	-0.72674
b_{23}	-0.3168	-0.32100
b_{24}	-0.9792	-0.98775
a_3	1.53	1.48293
b_{31}	-3.5649	-3.52631
b_{32}	0	0.03573
b_{33}	-1.53	-1.50898
b_{34}	-0.7191	-0.67335
a_4	1.27	1.25074
b_{41}	-1.5367	-1.52127
b_{42}	-0.6477	-0.63310
b_{43}	-0.4445	-0.43667
b_{44}	-1.27	-1.25061

	MAR	MAR with log transformation
β_{11}	0.85600	1.00059
β_{21}	0.26400	0.20355
β_{31}	-0.16900	-0.79454
β_{41}	-0.21800	-0.23777
β_{12}	-0.14300	-0.10901
β_{22}	0.88800	0.88053
β_{32}	0.01640	0.59018
β_{42}	0.02020	0.08015
β_{13}	-0.47500	-0.03240
β_{23}	0.10500	0.03330
β_{33}	1.07000	0.76513
β_{43}	0.06420	-0.03995
β_{14}	0.38200	0.17765
β_{24}	-0.24000	-0.23158
β_{34}	-0.21500	0.90744
β_{44}	0.80900	1.08027
α_1	0.03510	0.01644
α_2	0.04320	-0.01131
α_3	0.11000	-0.11896
α_4	0.11500	-0.24402
δ_1	0.00002	0.00114
δ_2	0.00002	0.00015
δ_3	0.00022	0.00191
δ_4	0.00006	0.00024

Table S3.6 – Initial conditions, parameter values and estimates for a four-variable LV system displaying deterministic chaos (chaos 2) as presented in Figure 3f.

	Initial Condition	Estimate
X_1	0.3	0.3
X_2	0.3	0.3
X_3	0.4	0.4
X_4	0.6	0.6

	True Parameter Value	ALVI-MI
a_1	0.3	0.29277
b_{11}	-0.3	-0.25379
b_{12}	-0.27	-0.28051
b_{13}	-0.6	-0.55795
b_{14}	-0.045	-0.07045
a_2	0.4	0.39048
b_{21}	0.2	0.26661
b_{22}	-0.4	-0.41601
b_{23}	-0.4	-0.33988
b_{24}	-0.6	-0.63818
a_3	0.8	0.77659
b_{31}	-2.38	-2.23922
b_{32}	0.35	0.31928
b_{33}	-2.45	-2.32117
b_{34}	0.35	0.27470
a_4	0.6	0.58578
b_{41}	-0.96	-0.86962
b_{42}	-0.24	-0.26048
b_{43}	-0.96000	-0.87774
b_{44}	-0.30000	-0.34969

	MAR	MAR with log transformation
β_{11}	1.05000	0.97700
β_{21}	0.00560	0.02030
β_{31}	-0.31600	-0.08880
β_{41}	-0.23000	-0.06400
β_{12}	-0.09830	-0.01780
β_{22}	0.99000	1.00000
β_{32}	0.08310	-0.02620
β_{42}	-0.01940	-0.01610
β_{13}	-0.00541	-0.06900
β_{23}	-0.12100	-0.07290
β_{33}	0.70200	0.87400
β_{43}	-0.18000	-0.05050
β_{14}	-0.08040	0.05960
β_{24}	0.00941	-0.02020
β_{34}	0.07400	-0.14500
β_{44}	0.97300	0.94100
α_1	0.05270	-0.11500
α_2	0.02820	-0.09870
α_3	0.07760	-0.53700
α_4	0.10200	-0.28000
δ_1	0.00000	0.00006
δ_2	0.00002	0.00023
δ_3	0.00009	0.00115
δ_4	0.00007	0.00025

Table S4.1 – ALVI-MI estimates for five experimental datasets from (Mühlbauer et al., 2020)**.** Data came from experiments described in (Gause, 1934), (McLaren and Peterson, 1994) and (Huffaker, 1958). See R package gauseR (Mühlbauer et al., 2020) for datasets "gause_1934_science_f02_03", "gause_1934_book_f32", "mclaren_1994_f03" and "huffaker_1963" for details on observations. Parameter estimates from Mühlbauer *et al.* can also be found in Table S8 in their paper.

Example 1 - *Paramecium caudatum* in monoculture. The slopes were estimated from an 8DF-spline from data without log transformation and ALVI-MI using a subsample of spline points at the 3rd and 12th days.

	Mühlbauer *et al.*	Estimate	Absolute Difference
a_1	1.259	0.92289	0.33611
b_{11}	-0.005	-0.00456	0.00044

Example 2 - *Paramecium caudatum* and *Paramecium aurelia* in a mixed population competition study. ALVI-MI was estimated from 10DF and 7DF-splines for *P. caudatum* and *P. aurelia*, respectively. Spline points were taken at days 4, 8 and 11.

	Mühlbauer *et al.*	Estimate	Absolute Difference
a_1	1.259	0.98677	0.27223
b_{11}	-0.005	-0.00409	0.00091
b_{12}	1.259	-0.00649	1.26549
a_2	-0.005	0.79868	0.80368
b_{21}	1.259	-0.00136	1.26036
b_{22}	-0.005	-0.00536	0.00036

Example 3 - Predator-prey interactions between *Didinium nasutum* and *Paramecium caudatum*. ALVI-MI estimates were calculated using 14DF and 10DF-splines, respectively, using a subsample of the 122nd , 140th, 168th points of the second spline.

	Mühlbauer *et al.*	Estimate	Absolute Difference
a_1	1.099	1.70706	0.60806
b_{11}	-0.013	-0.03887	0.02587
b_{12}	-0.078	-0.11360	0.03560
a_2	-0.89	-1.27639	0.38639
b_{21}	0.084	0.14275	0.05875
b_{22}	-0.002	0.01565	0.01765

Example 4 - Multi-trophic dynamics for wolves, moose, and fir trees. ALVI-MI estimates were calculated using log-abundances and 8DF-splines. Spline points were chosen as a subsample corresponding to the years 1973, 1978, 1979 and 1982.

	Mühlbauer *et al.*	Estimate	Absolute Difference
a_1	0.01	-1.901823	1.91182
b_{11}	-0.003	0.028812	0.03181
b_{12}	0.00004	0.000003	0.00004
b_{13}	0	2.754448	2.75445
a_2	2.021	0.331244	1.68976
b_{21}	-0.088	-0.006836	0.08116
b_{22}	0	-0.000107	0.00011
b_{23}	0.002	-0.090569	0.09257
a_3	0.238	2.779411	2.54141
b_{31}	0	-0.051545	0.05154
b_{32}	-0.0002	0.000494	0.00069
b_{33}	-0.139	-4.693609	4.55461

Example 5 - Predator-prey interactions between *E. sexmaculatus* and *T. occidentalis.* ALVI-MI estimates were calculated using 15DF and 20DF-splines, respectively. The splines were constructed using log-abundances of the dependent variables, using a subsample of spline points corresponding to the 17th, 48th and 55th datapoints.

	Mühlbauer *et al.*	Estimate	Absolute Difference
a_1	0.187	0.11148	0.07552
b_{11}	0	0.00003	0.00003
b_{12}	-0.028	-0.02960	0.00160
a_2	-0.377	-0.80007	0.42307
b_{21}	0.0012	0.00251	0.00131
b_{22}	-0.024	-0.03144	0.00744

Table S4.2 – ALVI-LR estimates for five experimental datasets from (Mühlbauer et al., 2020)**.** Data came from (Gause, 1934), (McLaren and Peterson, 1994) and (Huffaker et al., 1963) experiments. See R package gauseR (Mühlbauer et al., 2020) datasets "gause_1934_science_f02_03", "gause_1934_book_f32", "mclaren_1994_f03" and "huffaker_1963" for details on observations. Parameter estimates from Mühlbauer *et al.* can also be found in Table S8 of their paper.

Example 1 - *Paramecium caudatum* in monoculture, analyzed with 8DF-spline

	Mühlbauer *et al.*	ALVI-LR Estimate	Absolute Difference
a_1	1.259	0.93948	0.31952
b_{11}	-0.005	-0.00465	0.00035

Example 2 - *Paramecium caudatum* and *Paramecium aurelia* in mixed population, analyzed with 10DF and 7DF-splines

	Mühlbauer *et al.*	ALVI-LR Estimate	Absolute Difference
a_1	1.259	0.85524	0.40376
b_{11}	-0.005	-0.00289	0.00211
b_{12}	-0.008	-0.00580	0.00220
a_2	1.026	0.84423	0.18177
b_{21}	-0.002	-0.00187	0.00013
b_{22}	-0.007	-0.00553	0.00147

Example 3 - Predator-prey interactions between *Didinium nasutum* and *Paramecium caudatum*, analyzed with 14DF and 10DF-splines

	Mühlbauer *et al.*	ALVI-LR Estimate	Absolute Difference
a_1	1.099	0.45652	0.64248
b_{11}	-0.013	0.02117	0.03417
b_{12}	-0.078	-0.11495	0.03695
a_2	-0.89	-0.98922	0.09922
b_{21}	0.084	0.16549	0.08149
b_{22}	-0.002	-0.01146	0.00946

Example 4 - Multi-trophic dynamics for wolves, moose, and fir trees, analyzed with 28DF, 24DF and 28DF-splines

	Mühlbauer *et al.*	ALVI-LR Estimate	Absolute Difference
a_1	0.01	-0.06509	0.07509
b_{11}	-0.003	0.00164	0.00464
b_{12}	0.00004	0.00007	0.00003
b_{13}	0	-0.13411	0.13411
a_2	2.021	0.20754	1.81346
b_{21}	-0.088	-0.00483	0.08317
b_{22}	0	-0.00009	0.00009
b_{23}	0.002	0.06010	0.05810
a_3	0.238	-0.08580	0.32380
b_{31}	0	0.00343	0.00343
b_{32}	-0.0002	-0.00020	0.00000
b_{33}	-0.139	0.43352	0.57252

Example 5 - Predator-prey interactions between *E. sexmaculatus* and *T. occidentalis*, analyzed with 15DF and 20DF-splines

	Mühlbauer *et al.*	ALVI-LR Estimate	Absolute Difference
a_1	0.344	0.03525	0.30875
b_{11}	0	0.00038	0.00038
b_{12}	-0.059	-0.03619	0.02281
a_2	-0.236	-0.44687	0.21087
b_{21}	0.0005	0.00159	0.00109
b_{22}	0	-0.03540	0.03540

Table S4.3 – MAR estimates for five experimental datasets from (Mühlbauer et al., 2020)**.** Data came from (Gause, 1934), (McLaren and Peterson, 1994) and (Huffaker et al., 1963) experiments. See R package gauseR (Mühlbauer et al., 2020) for datasets “gause_1934_science_f02_03”, “gause_1934_book_f32”, “mclaren_1994_f03” and “huffaker_1963” for details on observations. Parameter estimates from Mühlbauer *et al.* can also be found in Table S8 of their paper.

Example 1 - *Paramecium caudatum* in monoculture.

	MAR	MAR log transformation	MAR with data smoothing	MAR log transformation with smoothing
β_1	0.90000	0.85550	0.90400	0.74718
α_1	9.93000	0.15590	10.22600	0.21106
δ_1	287.57000	0.06920	126.15300	0.00525

Example 2 - *Paramecium caudatum* and *Paramecium aurelia* in coculture

	MAR	MAR log transformation	MAR with data smoothing	MAR log transformation with smoothing
β_{11}	0.81800	0.73590	0.91500	0.98309
β_{21}	0.07170	0.03240	0.11600	0.03625
β_{12}	-0.16030	-0.07020	-0.16900	-0.26372
β_{22}	0.82140	0.71450	0.87700	0.73153
α_1	27.54890	1.38260	0.91000	0.07810
α_2	20.30740	1.28150	5.82700	0.15430
δ_1	262.75870	0.17190	113.99000	0.03530
δ_2	253.59620	0.03470	11.12100	0.00202

Example 3 - Predator-prey interactions between *Didinium nasutum* and *Paramecium caudatum*

	MAR	MAR log transformation	MAR with data smoothing	MAR log transformation with smoothing
β_{11}	0.52400	0.80250	0.37800	0.86900
β_{21}	0.57700	0.10510	0.52100	0.18700
β_{12}	-0.57200	-0.21950	-0.45100	-0.25400
β_{22}	0.63300	0.59490	0.69100	0.76500
α_1	12.93300	-1.11100	0.40300	-0.75400
α_2	-2.76100	0.06450	0.23900	0.70000
δ_1	94.22700	10.97630	140.72600	7.15700
δ_2	27.95400	23.00840	83.62600	9.82300

Example 4 - Multi-trophic dynamics for wolves, moose, and fir trees

	MAR	MAR log transformation	MAR with data smoothing	MAR log transformation with smoothing
β_{11}	0.62900	0.69390	1.13000	1.17360
β_{21}	-4.15000	-0.04540	-3.48000	-0.04426
β_{31}	-0.00083	-0.02210	0.00137	0.07236
β_{12}	0.00517	0.07570	0.00148	0.01665
β_{22}	0.84800	0.86270	0.90300	0.89080
β_{32}	-0.00007	-0.12840	-0.00009	-0.13234
β_{13}	-27.30000	-0.34650	10.70000	0.22128
β_{23}	96.60000	0.08020	245.00000	0.13054
β_{33}	0.89000	0.89160	1.11000	1.06497
α_1	15.90000	0.12600	-0.35700	-0.02613
α_2	241.00000	1.18230	27.80000	0.02986
α_3	0.14200	0.85670	-0.00485	-0.01065
δ_1	32.30000	0.06210	3.88000	0.00553
δ_2	15000.00000	0.01080	1290.00000	0.00127
δ_3	0.00382	0.02160	0.00098	0.00591

Example 5 - Predator-prey interactions between *E. sexmaculatus* and *T. occidentalis*

	MAR	MAR log transformation	MAR with data smoothing	MAR log transformation with smoothing
β_{11}	1.01500	0.88120	1.11000	1.05163
β_{21}	0.00900	0.53850	0.00718	0.47337
β_{12}	-17.79700	-0.09960	-16.00000	-0.10626
β_{22}	0.56200	0.75520	0.73200	0.86330
α_1	94.24600	0.80680	0.47700	-0.00088
α_2	-1.28500	-2.86420	-0.02580	-0.01771
δ_1	15440.27300	0.13470	1360.00000	0.02078
δ_2	5.55100	0.33790	1.93000	0.08104

Table S5.1 – Synthetic MAR data without noise

MAR Results

t	X_1	X_2	X_3	X_4
1	5	10	15	20
2	5.62418	35.2103	21.4125	10.3696
3	13.077	68.6603	16.2175	6.98986
4	30.8946	57.2944	7.75681	8.13234
5	42.7475	27.4855	3.71568	13.9927
6	31.3421	13.2593	2.63367	24.3619
7	16.5916	9.92497	3.02139	31.2622
8	9.868	12.4469	4.56419	27.4996
9	8.72248	20.3998	6.5998	19.5587
10	11.2768	30.657	7.33895	14.4998
11	16.7965	33.724	6.20514	13.314
12	21.9882	27.4413	4.66674	15.2345
13	22.1248	19.9474	3.79027	19.1227
14	18.0405	16.0646	3.69825	22.5386
15	14.0932	15.9557	4.22409	22.9275
16	12.3388	18.7495	5.03802	20.5772
17	12.7869	22.8183	5.59201	17.8653
18	14.8002	25.3774	5.51118	16.4484
19	17.1129	24.6804	4.99159	16.6605
20	18.1251	21.9731	4.48974	18.0462
21	17.3171	19.5701	4.27918	19.6396
22	15.6847	18.6862	4.39198	20.3941
23	14.456	19.3693	4.70288	19.9587
24	14.1822	20.9554	4.99681	18.8896
25	14.7787	22.3651	5.08888	17.9987
26	15.7517	22.7146	4.95911	17.7463
27	16.4482	21.9861	4.74141	18.1246
28	16.4701	20.8982	4.58774	18.7896
29	15.9468	20.1878	4.56995	19.2878
30	15.3325	20.1628	4.6678	19.3417
31	15.0101	20.6857	4.80099	19.0122

Table S5.2 –Synthetic MAR data with process noise (noisy MAR)

Noisy MAR data

t	X_1	X_2	X_3	X_4
1	5	10	15	20
2	5.348711	35.67645	21.24414	11.33115
3	12.68871	73.68564	15.69769	7.602699
4	28.4417	58.22924	7.677451	8.36752
5	40.38627	31.11175	3.80337	13.74682
6	31.35761	15.43932	2.37942	29.43823
7	16.35921	11.50274	2.862113	36.3036
8	9.354332	12.98424	4.058828	29.35258
9	7.685839	21.35519	5.988792	24.13497
10	10.19144	32.84542	6.76258	14.9041
11	16.91705	37.57379	6.463126	14.60513
12	22.32645	34.09477	4.028325	16.32554
13	23.42601	24.50092	3.053426	22.88628
14	19.95015	13.80835	2.855883	21.25741
15	14.53545	15.54018	2.687748	28.09769
16	10.56748	19.46347	4.100027	23.3091
17	11.6392	23.78792	4.736091	24.28919
18	13.60684	24.85652	5.581968	18.72783
19	15.55572	25.64391	5.618706	15.15855
20	16.01914	28.48243	3.98025	16.41035
21	17.42047	23.53516	3.913988	15.10063
22	20.59512	19.4383	3.094358	15.69554
23	20.04429	14.90893	3.033626	24.03874
24	16.92779	13.64164	3.412567	29.75478
25	10.70504	13.47052	4.30823	32.5117
26	8.284209	21.29853	5.626362	18.91741
27	11.17606	35.06573	5.989669	16.06294
28	15.30398	33.2859	4.745014	13.83807
29	24.07927	26.15628	3.85889	16.68449
30	20.98646	15.34366	3.163762	21.75982
31	16.75627	13.3735	3.46612	29.3738

Table S5.3 – Synthetic MAR data with process noise and replicates (replicate MAR)

t	X_1	X_2	X_3	X_4
1	5	10	15	20
1	5	10	15	20
1	5	10	15	20
1	5	10	15	20
1	5	10	15	20
2	6.824361	35.16599	18.89286	11.20497
2	7.140826	37.90816	19.56782	11.1861
2	6.419448	43.02967	24.34981	10.49169
2	6.05405	41.2587	23.90904	9.399141
2	5.210658	36.29504	22.36168	10.34802
3	15.28585	52.04399	15.13403	7.238021
3	16.98281	65.05571	13.10156	7.05455
3	16.57298	85.22612	18.48274	7.349583
3	16.19917	63.11921	18.64078	7.702582
3	11.00963	58.04277	18.08912	7.002301
4	26.611	39.69185	7.772539	7.884168
4	34.47372	39.76995	6.506274	10.41084
4	41.175	50.93835	7.71743	10.0942
4	31.16793	48.70791	8.994543	8.972057
4	28.31321	50.56873	9.315256	6.666504
5	27.51418	19.49706	4.270684	14.97806
5	37.22747	18.55792	3.621166	20.63883
5	46.13679	21.27444	3.731755	14.89004
5	34.42344	30.07884	4.993	15.49867
5	40.20807	27.69478	3.68488	13.15327
6	19.3429	13.61173	3.958714	22.04277
6	17.05215	11.34199	3.278152	30.66941
6	33.02304	9.387348	3.430976	22.62625
6	27.13532	17.18788	3.897519	22.08733
6	26.45495	14.35704	2.647763	25.19314
7	15.76911	14.08855	4.4589	28.75703
7	10.05276	13.22159	4.477918	27.6154
7	16.57564	10.77569	4.304613	30.14191
7	19.26835	14.48505	4.079162	24.61664
7	16.12083	9.838952	3.340428	32.4906
8	11.18645	15.10148	6.138031	22.90268
8	7.935696	26.17905	6.136483	19.06441
8	9.725832	16.558	7.075358	24.15163
8	13.71308	12.90949	4.408927	25.66316
8	8.855132	10.79515	5.2759	34.28205
11	18.35118	40.96935	7.867435	12.23562
11	24.0257	27.39741	3.152885	15.61119
11	22.36637	32.77024	6.421379	12.83701
11	15.58502	37.86238	4.60214	11.52865
11	20.88114	35.67158	5.660139	12.95133
14	14.05995	12.13665	3.591629	20.26163
14	11.19216	14.35631	4.181295	22.72751
14	12.04293	18.32562	4.884143	21.96059
14	18.03127	12.6617	2.720828	25.28884
14	13.23772	15.31324	4.582637	20.50827
17	14.61293	23.61389	5.714322	18.331
17	17.62079	30.33241	6.897818	15.35878
17	18.80657	31.36387	5.839329	13.26646
17	10.34518	21.73182	5.972099	18.41633
17	15.03356	23.89785	4.270884	15.44892

20	21.07394	19.92159	4.706945	17.26361
20	20.76206	23.40946	4.342067	16.35985
20	15.42562	14.47828	4.306908	21.14931
20	20.95617	21.4038	3.978127	15.6198
20	13.75127	16.88018	3.678261	22.68312
23	13.41462	17.15951	5.90813	19.95043
23	14.64681	20.98447	4.511556	18.69095
23	15.28076	19.79288	5.263899	16.61078
23	12.595	18.04093	4.308493	22.52222
23	16.08145	29.17789	5.73419	13.7156
26	17.12985	27.17032	5.198294	14.47482
26	13.20432	27.43056	5.320148	17.11185
26	18.08855	19.68478	3.087013	15.64236
26	18.31181	24.89349	5.215812	16.24242
26	18.62125	20.08891	3.991524	23.46726
29	18.3059	19.77749	4.947163	16.09594
29	17.88635	21.30849	4.690069	20.27669
29	11.76912	13.69399	3.731459	24.47256
29	19.4553	15.7721	4.754019	19.41696
29	16.04477	20.65494	5.300268	17.14123

Table S5.4 – Initial conditions and ALVI-MI parameters estimates for the synthetic MAR data

	Noise MAR	Replicate MAR
Spline	15DF-spline	11DF-spline
Point subsample	*t* = 2, 4, 7, 22, 27	*t* = 1, 3, 11, 13, 15
	Initial condition	Initial condition
X_1	4.421	4.609
X_2	11.830	11.745
X_3	17.262	16.820
X_4	18.619	18.670
Parameter	Estimate	Estimate
a_1	0.873	1.930
b_{11}	-0.012	-0.022
b_{12}	0.007	0.001
b_{13}	-0.001	-0.016
b_{14}	-0.040	-0.082
a_2	0.539	1.112
b_{21}	-0.044	-0.035
b_{22}	-0.001	-0.016
b_{23}	0.037	0.041
b_{24}	0.000	-0.023
a_3	0.242	-0.623
b_{31}	-0.019	-0.014
b_{32}	-0.010	-0.006
b_{33}	0.000	0.021
b_{34}	0.013	0.047
a_4	-0.412	-0.809
b_{41}	0.040	0.030
b_{42}	-0.005	0.007
b_{43}	-0.006	-0.013
b_{44}	-0.004	0.013
Estimates vs Data SSE	1221.9	385.3

Table S5.5 – Initial conditions and ALVI-LR parameters estimates for the synthetic MAR data

	Noise MAR	Replicate MAR
Spline	25,14,18,20, DF-spline	15DF-spline
	Initial condition	Initial condition
X_1	4.874	5
X_2	12.366	10
X_3	16.270	15
X_4	19.662	20
Parameter	Estimate	Estimate
a_1	0.622	0.412
b_{11}	-0.014	-0.016
b_{12}	0.015	0.016
b_{13}	-0.009	0.000
b_{14}	-0.034	-0.027
a_2	0.383	0.756
b_{21}	-0.030	-0.034
b_{22}	-0.005	-0.017
b_{23}	0.043	0.060
b_{24}	0.001	-0.007
a_3	0.239	0.321
b_{31}	-0.020	-0.019
b_{32}	-0.011	-0.014
b_{33}	0.003	0.013
b_{34}	0.015	0.012
a_4	-0.531	-0.580
b_{41}	0.033	0.031
b_{42}	0.000	0.003
b_{43}	-0.011	-0.018
b_{44}	0.002	0.005
Estimates vs Data SSE	1360.4	754.2

Table S6.1 – Parameter values and initial conditions estimated for the 'grey whales' dataset (Gerber et al., 1999)

Results generated with ALVI-MI with 3DF-spline and a data sample composed of spline values in 1959 and 1966.

Parameter	True value	Estimate
**ALVI-MI**		
a_1		0.0948
b_{11}		-3.79E-06
$X_1(0)$	2894	3663.9550
**MAR**		
α_1		1260
β_{11}		0.943
δ_1		7240000
$X_1(0)$	2894	
**MAR with log transformation**		
α_1		1.0368
β_{11}		0.9512
δ_1		0.0327
$X_1(0)$	2894	
**MAR with smoothing**		
α_1		597
β_{11}		0.9930
δ_1		199000
$X_1(0)$	2894	
**MAR with log transformation and smoothing**		
α_1		0.4902
β_{11}		0.9535
δ_1		0.0014
$X_1(0)$	2894	

Table S6.2 – Parameters and initial conditions estimated for the 'Wolves and Moose' dataset (Vucetich, 2021)

Results of ALVI-MI with 15DF-splines and a data sample composed of spline values in 1991, 1994 and 1997.

ALVI-MI

	Initial condition	Estimate
X_1	20	21.6545
X_2	538	560.5340

Parameter	Estimate
a_1	-0.2732
b_{11}	0.0073
b_{12}	0.0001
a_2	0.8431
b_{21}	-0.0380
b_{22}	-0.0001

MAR with log transformation

	Initial condition	Estimate
X1	20	22.0000
X2	538	564.0000

Parameter	Estimate
β11	0.7670
β21	-0.1788
β12	0.0783
β22	0.8277
δ1	0.4485
δ2	0.1758

Table S7 - Results of one-sided Wilcoxon rank test.

Null hypothesis H_0:	Alternative hypothesis H_1:	*p*-value
SSE values of ALVI-MI are equal or higher than corresponding MAR values	SSE values of ALVI-MI are less than corresponding MAR values	**0.0024**
SSE values of ALVI-MI are equal or higher than corresponding MAR values for log transformed variables	SSE values of ALVI-MI are less than corresponding MAR values for log transformed variables	**0.0034**
SSE values of ALVI-MI are equal or higher than corresponding MAR values for smoothed data	SSE values of ALVI-MI are less than corresponding MAR values for smoothed data	0.0508
SSE values of ALVI-MI are equal or higher than corresponding MAR values for log transformed variables and smoothed data	SSE values of ALVI-MI are less than corresponding MAR values for log transformed variables and smoothed data	**0.0024**
SSE values of the ALVI-MI are equal or higher than SSE values obtained with the ALVI-LR	SSE values of ALVI-MI are less than SSE values obtained with the ALVI-LR	**0.0010**
SSE values of MAR with log transform are equal or higher than MAR values	SSE values of MAR with log transform are less than MAR values	0.3501
SSE values of MAR with data smoothing are equal or higher than MAR values without	SSE values of MAR with data smoothing are less than MAR values without	0.6812
SSE values of MAR with log transformation and data smoothing are equal or higher than those for MAR with log transformation values	SSE values of MAR with log transformation and data smoothing are less than those for MAR with log transformation values	0.6177

Table S8 – Sum of absolute differences between true and estimated parameters. Bold font indicates the lower difference in each case.

	ALVI-LR	ALVI-MI	MAR	MAR log Trans	MAR with smoothing	MAR with log and smoothing
Noisy LV data	0.0143	**0.0139**				
Replicate LV data	0.0070	**0.0056**				
Noisy MAR data			**4.2682**	9.3141	7.3977	11.4039
Replicate MAR data			**10.6323**	12.6328	11.0209	11.2835
SynthData1	**0.0002**	0.0004				
SynthData2	0.2083	**0.1553**				
SynthData3	1.0698	**0.0982**				
SynthData4	**0.1918**	0.6080				
SynthData5	0.8240	**0.3621**				
SynthData6	**0.1879**	0.9779				

Table S9.1 – SSEs for the LV datasets. The model presented in Figure 1 was initiated with different values that were chosen as ratios of the system steady state. The Table shows the SSEs of the noise-free data and the fits from the different methods when the data were obtained by **(a)** random sampling and **(b)** when each timepoint was sampled five times.

SSEs

Noisy LV dataset

	STST * 0.001	STST * 0.01	STST * 0.1	STST * 1.9	STST * 10	STST * 100
ALVI-MI method	38.792	4.98419	4.149	1.098	43.056	38.489
MAR no transform	587.982	238.8986	21.554	0.47	37.478	218.094
MAR log transform	621.077	136.7711	5.189	0.358	11.017	50.584
MAR no transform with smoothing	504.071	240.2219	58.076	4.032	776.266	66825.211
MAR log transform with smoothing	752.466	186.2173	22.637	4.33	718.701	60118.749

Replicate LV dataset

	STST * 0.001	STST * 0.01	STST * 0.1	STST * 1.9	STST * 10	STST * 100
ALVI-MI method	143.767	14.14818	1.137	0.852	16.599	252.005
MAR no transform	605.644	237.7721	10.323	0.534	386.576	72524.754
MAR log transform	694.657	146.5051	3.829	0.175	16.362	6430.865
MAR no transform with smoothing	566.840	238.8223	20.658	1.372	312.168	34112.580
MAR log transform with smoothing	537.914	151.4522	13.394	1.562	270.309	27135.802

Table S9.2 - Steady-State SSEs for the LV datasets. The model presented in Figure 1 was initiated with different values that were chosen as ratios of the system steady state. The objective here was to evaluate how well the different methods estimate the steady state of the model. The Table shows the sum of SSEs between the last five datapoints of noise free data and the fits from the different methods when the data were obtained by **(a)** random sampling and **(b)** when each timepoint was sampled five times.

Steady State SSEs

Noisy LV dataset

	STST * 0.001	STST * 0.01	STST * 0.1	STST * 1.9	STST * 10	STST * 100
ALVI-MI method	0.107	0.107	0.026	0.017	0.266	0.535
MAR no transform	2.661	0.202	0.095	0.01	0.231	0.218
MAR log transform	0.651	0.112	0.01	0.012	0.174	0.171
MAR no transform with smoothing	0.983	0.04	5.643	0.015	0.242	0.23
MAR log transform with smoothing	1.212	0.408	0.384	0.01	0.296	0.793

Replicate LV dataset

	STST * 0.001	STST * 0.01	STST * 0.1	STST * 1.9	STST * 10	STST * 100
ALVI-MI method	4.628	0.06	0.016	0.029	0.562	0.069
MAR no transform	6.377	0.47	0.007	0.011	0.072	14.18
MAR log transform	2.93	0.184	0.02	0.005	0.083	0.106
MAR no transform with smoothing	0.27	0.846	0.51	0.003	0.253	0.084
MAR log transform with smoothing	36.118	1.092	0.041	0.003	0.119	0.323

Table S9.3: Number of parameter estimates with signs opposite to the true parameters in the artificial LV system. The model presented in Figure 1 was initiated with different values that were chosen as ratios of the system steady state. To infer the accuracy of the parameter estimation methods, we counted how many estimates have the opposite sigs in comparison with the original parameter. The Table shows the number of sign changes in the estimates relative to the original parameters from the different methods when the data were obtained by **(a)** random sampling and **(b)** when each timepoint was sampled five times.

Signal flips

Noisy LV dataset

	STST * 0.001	STST * 0.01	STST * 0.1	STST * 1.9	STST * 10	STST * 100
ALVI-MI method	0	0	3	3	4	7
MAR no transform	7	8	7	5	6	4
MAR log transform	6	7	7	5	5	5
MAR no transform with smoothing	6	6	6	10	9	11
MAR log transform with smoothing	5	6	5	10	9	9

Replicate LV dataset

	STST * 0.001	STST * 0.01	STST * 0.1	STST * 1.9	STST * 10	STST * 100
ALVI-MI method	0	2	0	3	4	4
MAR no transform	6	7	7	7	5	7
MAR log transform	7	7	6	4	7	6
MAR no transform with smoothing	6	6	6	10	12	12
MAR log transform with smoothing	6	6	6	9	10	10

Table S10.1 – SSEs for the MAR datasets. The model presented in Figure 1 was initiated with different values that were chosen as ratios of the system steady state. The Table shows the SSEs of the noise-free data and the fits from the different methods when the data were obtained by **(a)** random sampling and **(b)** when each timepoint was sampled five times.

SSEs

Noisy LV dataset

	STST * 0.001	STST * 0.01	STST * 0.1	STST * 1.9	STST * 10	STST * 100
ALVI-MI method	1892.33	1251.964	426.909	224.814	6550.551	24108.298
MAR no transform	350.043	253.891	221.893	234.885	249.726	719.055
MAR log transform	291.211	252.903	233.242	137.237	156.572	259.335
MAR no transform with smoothing	1391.574	929.278	511.268	797.641	6022.958	198393.566
MAR log transform with smoothing	3350.012	1937.803	1033.486	1890.82	5828.088	154629.128

Replicate LV dataset

	STST * 0.001	STST * 0.01	STST * 0.1	STST * 1.9	STST * 10	STST * 100
ALVI-MI method	368.937	163.717	80.936	137.877	359.973	15637.372
MAR no transform	307.720	131.085	68.772	101.993	243.564	1777.944
MAR log transform	59.903	75.688	90.422	88.308	292.391	1258.105
MAR no transform with smoothing	346.174	247.237	148.283	132.142	2474.397	104256.158
MAR log transform with smoothing	1245.179	559.108	80.936	137.877	2259.931	95591.528

Table S10.2 - Steady State SSEs for the MAR datasets. The model presented in Figure 2 was initiated with different values that were chosen as ratios of the system steady state. The objective here was to evaluate how well the different methods estimate the steady state of the model. The Table shows the SSEs between the last five datapoints of noise-free data and the fits from the different methods when the data were obtained by **(a)** random sampling and **(b)** when each timepoint was sampled five times.

Steady State SSEs

Noisy LV dataset

	STST * 0.001	STST * 0.01	STST * 0.1	STST * 1.9	STST * 10	STST * 100
ALVI-MI method	393.039	45.222	7.144	16.774	22.97	7.312
MAR no transform	26.146	30.431	34.502	31.172	25.454	22.523
MAR log transform	28.682	25.184	22.228	18.028	17.369	19.097
MAR no transform with smoothing	64.236	42.837	16.971	383.562	389.408	58.176
MAR log transform with smoothing	237.034	453.91	152.844	730.076	261.093	404.454

Replicate LV dataset

	STST * 0.001	STST * 0.01	STST * 0.1	STST * 1.9	STST * 10	STST * 100
ALVI-MI method	0.87	0.806	3.464	4.606	21.093	2742.131
MAR no transform	6.324	2.153	5.7	0.966	1.238	16.241
MAR log transform	1.303	1.085	3.115	0.537	0.481	1.279
MAR no transform with smoothing	4.922	6.002	13.272	1.01	0.774	6.148
MAR log transform with smoothing	1.227	1.197	10.11	0.576	1.518	1.318

Table S10.3: Number of parameter estimates with sign opposite to the true parameter in the artificial MAR system. The model presented in Figure 2 was initiated with different values that were chosen as ratios of the system steady state. To infer the accuracy of the parameter estimation methods, we counted how many estimates have the opposite sign compared with the original parameter. The Table shows the number of signal changes in the estimates relative to the original parameters from the different methods when the data were obtained by **(a)** random sampling and **(b)** when each timepoint was sampled five times.

Signal flips

Noisy LV dataset

	STST * 0.001	STST * 0.01	STST * 0.1	STST * 1.9	STST * 10	STST * 100
ALVI-MI method	8	8	7	5	9	10
MAR no transform	1	1	1	1	1	11
MAR log transform	1	1	1	3	1	1
MAR no transform with smoothing	1	1	1	0	5	4
MAR log transform with smoothing	4	4	0	10	8	6

Replicate LV dataset

	STST * 0.001	STST * 0.01	STST * 0.1	STST * 1.9	STST * 10	STST * 100
ALVI-MI method	9	7	9	6	5	8
MAR no transform	1	1	1	7	7	8
MAR log transform	1	7	1	7	8	6
MAR no transform with smoothing	7	8	2	9	10	10
MAR log transform with smoothing	3	4	2	8	10	6

Table S11: Degrees of freedom used in the initial condition study.

LV artificial data

	noisy dataset				replicate dataset			
STST * 0.001	40	40	40	8	10	10	10	8
STST * 0.01	30	30	30	8	10	10	10	8
STST * 0.1	8	8	8	8	8	8	8	8
STST * 1.9	8	8	8	8	8	8	8	8
STST * 10	8	8	8	8	8	8	8	8
STST * 100	40	40	40	40	11	11	11	11

MAR artificial data

	noisy dataset				replicate dataset			
STST * 0.001	8	8	8	8	8	8	8	8
STST * 0.01	8	8	8	8	8	8	8	8
STST * 0.1	8	8	8	8	8	8	8	8
STST * 1.9	8	8	8	8	8	8	8	8
STST * 10	8	8	8	8	8	8	8	8
STST * 100	8	8	8	8	8	8	8	8

6 References

Batista Júnior, A.B., Pires, P.S.M., 2014. An Approach to Outlier Detection and Smoothing Applied to a Trajectography Radar Data. Journal of Aerospace Technology and Management 6, 237–248. https://doi.org/10.5028/jatm.v6i3.325

Burden, R.L., Faires, J.D., Burden, A.M., 1993. Numerical Analysis, Fifth edit. ed. PWS Publishing Co., Boston, MA.

Certain, G., Barraquand, F., Gårdmark, A., 2018. How do MAR(1) models cope with hidden nonlinearities in ecological dynamics? Methods Ecol Evol 9, 1975–1995. https://doi.org/10.1111/2041-210X.13021

Chiang, S.-Y., 2012. An application of Lotka–Volterra model to Taiwan's transition from 200mm to 300mm silicon wafers. Technol Forecast Soc Change 79, 383–392. https://doi.org/10.1016/j.techfore.2011.05.007

Cleveland, W.S., 1981. LOWESS: A program for smoothing scatterplots by robust locally weighted regression. Am Stat 35, 54. https://doi.org/10.2307/2683591

Cleveland, W.S., 1979. Robust Locally Weighted Regression and Smoothing Scatterplots. J Am Stat Assoc 74, 829–836. https://doi.org/10.1080/01621459.1979.10481038

Cleveland, W.S., Devlin, S.J., 1988. Locally Weighted Regression: An Approach to Regression Analysis by Local Fitting. J Am Stat Assoc 83, 596–610. https://doi.org/10.1080/01621459.1988.10478639

Cleveland, W.S., Grosse, E., 1991. Computational methods for local regression. Stat Comput 1, 47–62. https://doi.org/10.1007/BF01890836

Dam, P., Fonseca, L.L., Konstantinidis, K.T., Voit, E.O., 2016. Dynamic models of the complex microbial metapopulation of lake mendota. NPJ Syst Biol Appl 2, 1–7. https://doi.org/10.1038/npjsba.2016.7

Dam, P., Rodriguez-R, L.M., Luo, C., Hatt, J., Tsementzi, D., Konstantinidis, K.T., Voit, E.O., 2020. Model-based comparisons of the abundance dynamics of dacterial communities in two lakes. Sci Rep 10, 1–12. https://doi.org/10.1038/s41598-020-58769-y

Eilers, P.H.C., 2003. A Perfect Smoother. Anal Chem 75, 3631–3636. https://doi.org/10.1021/ac034173t

Eilers, P.H.C., Marx, B.D., 1996. Flexible smoothing with B -splines and penalties. Statistical Science 11, 89–121. https://doi.org/10.1214/ss/1038425655

Fort, H., 2020. Introduction, in: Ecological Modelling and Ecophysics. IOP Publishing. https://doi.org/10.1088/978-0-7503-2432-8ch0

Gandolfo, G., 2008. Giuseppe Palomba and the Lotka-Volterra equations. Rendiconti Lincei 19, 347–357. https://doi.org/10.1007/s12210-008-0023-7

Garcia, D., 2010. Robust smoothing of gridded data in one and higher dimensions with missing values. Comput Stat Data Anal 54, 1167–1178. https://doi.org/10.1016/j.csda.2009.09.020

Gause, G.F., 1934. Experiemental analysis of Vito Volterra's mathematical theory of the struggle for existence. Science (1979) 79, 16–17. https://doi.org/10.1126/science.79.2036.16-a

Gerber, L.R., Demaster, D.P., Kareiva, P.M., 1999. Gray whales and the value of monitoring data in implementing the U.S. endangered species act. Conservation Biology 13, 1215–1219. https://doi.org/10.1046/j.1523-1739.1999.98466.x

Gutenkunst, R.N., Waterfall, J.J., Casey, F.P., Brown, K.S., Myers, C.R., Sethna, J.P., 2007. Universally Sloppy Parameter Sensitivities in Systems Biology Models. PLoS Comput Biol 3, 1871–1878. https://doi.org/10.1371/journal.pcbi.0030189

Haas, C.N., 1981. Application of predator-prey models to disinfection. Journal of the Water Pollution Control Federation 53, 378–386. https://doi.org/10.2307/25041087

Hacinliyan, A.S., Kusbeyzi, I., Aybar, O.O., 2010. Approximate solutions of Maxwell Bloch equations and possible Lotka Volterra type behavior. Nonlinear Dyn 62, 17–26. https://doi.org/10.1007/s11071-010-9695-5

Holmes, E.E., Ward, E.J., Scheuerell, M.D., 2020. Analysis of multivariate timeseries using the MARSS package.

Holmes, E.E., Ward, E.J., Wills, K., 2012. MARSS: multivariate autoregressive state-space models for analyzing time-series data. R J 4, 11. https://doi.org/10.32614/RJ-2012-002

Huffaker, C.B., 1958. Experimental studies on predation: Dispersion factors and predator-prey oscillations. Hilgardia 27, 343–383. https://doi.org/10.3733/hilg.v27n14p343

Hung, H.-C., Chiu, Y.-C., Huang, H.-C., Wu, M.-C., 2017. An enhanced application of Lotka–Volterra model to forecast the sales of two competing retail formats. Comput Ind Eng 109, 325–334. https://doi.org/10.1016/j.cie.2017.05.022

Hytti, H., Takalo, R., Ihalainen, H., 2006. Tutorial on Multivariate Autoregressive Modelling. J Clin Monit Comput 20, 101–108. https://doi.org/10.1007/s10877-006-9013-4

Ives, A.R., 1995. Predicting the response of populations to environmental change. Ecology 76, 926–941. https://doi.org/10.2307/1939357

Knowles, I., Renka, R.J., 2014. Methods for Numerical Differentiation of Noisy Data. Electronic Journal of Differential Equations 21, 235–246.

Lindström, T., 2019. Mathematics and Recurrent Population Outbreaks, in: Handbook of the Mathematics of the Arts and Sciences. Springer International Publishing, Cham, pp. 1–16. https://doi.org/10.1007/978-3-319-70658-0_33-1

Loader, C., 2012. Smoothing: Local Regression Techniques, in: Handbook of Computational Statistics. Springer Berlin Heidelberg, Berlin, Heidelberg, pp. 571–596. https://doi.org/10.1007/978-3-642-21551-3_20

McLaren, B.E., Peterson, R.O., 1994. Wolves, moose, and tree Rings on Isle Royale. Science (1979) 266, 1555–1558. https://doi.org/10.1126/science.266.5190.1555

Mühlbauer, L.K., Schulze, M., Harpole, W.S., Clark, A.T., 2020. gauseR: Simple methods for fitting Lotka-Volterra models describing Gause's "Struggle for Existence." Ecol Evol 10, 13275–13283. https://doi.org/10.1002/ece3.6926

Nambu, M., 1986. Plasma-maser effects in plasma astrophysics. Space Sci Rev 44, 357–391. https://doi.org/10.1007/BF00200820

Peschel, M., Mende, W., 1986. The Predator-Prey Model: Do we Live in a Volterra World? Akademie-Verlag, Berlin.

Ramsay, J.O., Hooker, G., Campbell, D., Cao, J., 2007. Parameter estimation for differential equations: a generalized smoothing approach. J R Stat Soc Series B Stat Methodol 69, 741–796. https://doi.org/10.1111/j.1467-9868.2007.00610.x

Savageau, M.A., Voit, E.O., 1987. Recasting nonlinear differential equations as S-systems: a canonical nonlinear form. Math Biosci 87, 83–115. https://doi.org/10.1016/0025-5564(87)90035-6

Smyth, G., 2020. Difference between LOESS and LOWESS. [WWW Document]. URL https://stats.stackexchange.com/questions/161069/difference-between-loess-and-lowess (accessed 9.2.21).

Srinath, S., Gunawan, R., 2010. Parameter identifiability of power-law biochemical system models. J Biotechnol 149, 132–140. https://doi.org/10.1016/j.jbiotec.2010.02.019

Stein, R.R., Bucci, V., Toussaint, N.C., Buffie, C.G., Rätsch, G., Pamer, E.G., Sander, C., Xavier, J.B., 2013. Ecological modeling from time-series inference: insight into dynamics and stability of intestinal microbiota. PLoS Comput Biol 9, 1–11. https://doi.org/10.1371/journal.pcbi.1003388

Vano, J.A., Wildenberg, J.C., Anderson, M.B., Noel, J.K., Sprott, J.C., 2006. Chaos in low-dimensional Lotka–Volterra models of competition. Nonlinearity 19, 2391–2404. https://doi.org/10.1088/0951-7715/19/10/006

Varah, J.M., 1982. A spline least squares method for numerical parameter estimation in differential equations. SIAM Journal on Scientific and Statistical Computing 3, 28–46. https://doi.org/10.1137/0903003

Vilela, M., Borges, C.C.H., Vinga, S., Vasconcelos, A.T.R., Santos, H., Voit, E.O., Almeida, J.S., 2007. Automated smoother for the numerical decoupling of dynamics models. BMC Bioinformatics 8, 1–8. https://doi.org/10.1186/1471-2105-8-305

Vilela, M., Vinga, S., Maia, M.A.G.M., Voit, E.O., Almeida, J.S., 2009. Identification of neutral biochemical network models from time series data. BMC Syst Biol 3, 1–13. https://doi.org/10.1186/1752-0509-3-47

Vogels, M., Zoeckler, R., Stasiw, D.M., Cerny, L.C., 1975. P. F. Verhulst's "notice sur la loi que la populations suit dans son accroissement" from correspondence mathematique et physique. Ghent, vol. X, 1838. J Biol Phys 3, 183–192. https://doi.org/10.1007/BF02309004

Voit, E.O., 2017. A First Course in Systems Biology, Second edi. ed. Garland Science. https://doi.org/10.1201/9780203702260

Voit, E.O., 2013. Biochemical systems theory: a review. ISRN Biomath 2013, 1–53. https://doi.org/10.1155/2013/897658

Voit, E.O., 2000. Canonical Modeling: Review of Concepts with Emphasis on Environmental Health. Environ Health Perspect 108, 895–909. https://doi.org/10.1289/ehp.00108s5895

Voit, E.O., Almeida, J., 2004. Decoupling dynamical systems for pathway identification from metabolic profiles. Bioinformatics 20, 1670–1681. https://doi.org/10.1093/bioinformatics/bth140

Voit, E.O., Almeida, J., 2003. Dynamic Profiling and Canonical Modeling, in: Metabolic Profiling: Its Role in Biomarker Discovery and Gene Function Analysis. Springer US, Boston, MA, pp. 257–276. https://doi.org/10.1007/978-1-4615-0333-0_14

Voit, E.O., Chou, I.-C., 2010. Parameter estimation in canonical biological systems models. Int J Syst Synth Biol 1, 1–19.

Voit, E.O., Davis, J.D., Olivença, D.V., 2021. Inference and validation of the structure of Lotka-Volterra models. bioRxiv. https://doi.org/10.1101/2021.08.14.456346

Voit, E.O., Olivença, D.V., 2022. Discrete Biochemical Systems Theory. Front Mol Biosci 9, 1–14. https://doi.org/10.3389/fmolb.2022.874669

Voit, E.O., Savageau, M.A., 1986. Equivalence between S-systems and Volterra systems. Math Biosci 78, 47–55. https://doi.org/10.1016/0025-5564(86)90030-1

Voit, E.O., Savageau, M.A., 1982a. Power-law approach to modeling biological systems; II. Application to ethanol production. J. Ferment. Technol. 60, 229–232.

Voit, E.O., Savageau, M.A., 1982b. Power-law approach to modeling biological systems; III. Methods of analysis. J. Ferment. Technol. 60, 233–241.

Vucetich, J.A., 2021. Wolves and moose of Isle Royale [WWW Document]. URL https://isleroyalewolf.org/

Xiao, Y., Angulo, M.T., Friedman, J., Waldor, M.K., Weiss, S.T., Liu, Y.-Y., 2017. Mapping the ecological networks of microbial communities. Nat Commun 8, 1–12. https://doi.org/10.1038/s41467-017-02090-2

Zhou, Y., Chen, B., 2006. Analysis of multi-ISPs game based on lotka-volterra model., in: CIMCA 2006: International Conference on Computational Intelligence for Modelling, Control and Automation, Jointly with IAWTIC 2006: International Conference on Intelligent Agents Web Technologies. IEEE Computer Society. https://doi.org/10.1109/CIMCA.2006.44
